# miR-142 deficit in T cells during blast crisis promotes chronic myeloid leukemia immune escape

**DOI:** 10.1038/s41467-025-56383-y

**Published:** 2025-02-01

**Authors:** Fang Chen, Dandan Zhao, Yongfang Xu, Yi Zhang, Min-Hsuan Chen, Khyatiben V. Pathak, Nate Hansen, Brooke Lovell, Yong Liang, Katrina Estrella, Wei-Le Wang, Lucy Ghoda, Russell Rockne, Xiwei Wu, Haris Ali, Jianhua Yu, Michael A. Caligiuri, Stephen J. Forman, Jeff M. Trent, Ya-Huei Kuo, Ling Li, Piotr Swiderski, Jianying Zhang, Marcin Kortylewski, Le Xuan Truong Nguyen, Patrick Pirrotte, Mark Boldin, Guido Marcucci, Bin Zhang

**Affiliations:** 1 Department of Hematological Malignancies Translational Science, Gehr Family Center for Leukemia Research, City of Hope Medical Center and Beckman Research Institute, Duarte, CA USA; 2 Department of Hematology, The First Affiliated Hospital, College of Medicine, Zhejiang University, Hangzhou, Zhejiang PR China; 3 Integrative Genomics Core, City of Hope Beckman Research Institute, Duarte, CA USA; 4 Department of Computational and Quantitative Medicine, City of Hope Beckman Research Institute, Duarte, CA USA; 5 Cancer & Cell Biology Division, Translational Genomics Research Institute, Phoenix, AZ USA; 6 Integrated Mass Spectrometry Shared Resource, City of Hope, Duarte, CA USA; 7 DNA/RNA Peptide Shared Resources, City of Hope Beckman Research Institute, Duarte, CA USA; 8 Department of Systems Biology, City of Hope Beckman Research Institute, Duarte, CA USA; 9 Department of Hematology & Hematopoietic Cell Transplantation, City of Hope National Medical Center, Duarte, CA USA; 10 Department of Immuno-Oncology, City of Hope Beckman Research Institute, Duarte, CA USA

**Keywords:** Chronic myeloid leukaemia, miRNAs, Cancer immunotherapy, Cancer stem cells

## Abstract

We reported that an acquired miR-142 deficit transforms chronic phase (CP) chronic myeloid leukemia (CML) leukemic stem cells (LSCs) into blast crisis (BC) LSCs. Given the role of miR-142 in the development and activity of the immune system, we postulated that this deficit also promotes LSC immune escape. Herein, we report on IL-6-driven miR-142 deficit occurring in T cells during BC transformation. In CML murine models, miR-142 deficit impairs thymic differentiation of lymphoid-primed multipotent progenitors (LMPP) into T cells and prevents T cells’ metabolic reprogramming, thereby leading to loss of T cells and leukemia immune escape. Correcting miR-142 deficit with a miR-142 mimic compound (M-miR-142), alone or in combination with immune checkpoint antibodies, restores T cell number and immune activity, leading to LSC elimination and prolonged survival of BC CML murine and patient-derived xenograft models. These observations may open new therapeutic opportunities for BC CML and other myeloid malignancies.

## Abstract (web summary)

Acquired miR-142 deficit in leukemic stem cells has been associated with chronic myeloid leukemia blast crisis. Here the authors show that acquired miR-142 deficit in T cells further enables leukemic stem cells to escape immune surveillance and supports disease growth.

## Introduction

Chronic myeloid leukemia (CML) is a myeloproliferative disorder characterized by the Philadelphia chromosome, a translocation of chromosomes 9q34 and 22q11, that creates the fusion oncogene *BCR::ABL1* encoding a constitutively activated tyrosine kinase (TK) mutant^1^. *BCR::ABL1* transforms normal hematopoietic stem cells (HSCs) into leukemia stem cells (LSCs), primitive leukemic cells capable of indefinite self-renewal and of initiating and maintaining the disease. Even though CML patients achieve disease remission with TK inhibitors (TKIs), they are often committed to a life-long treatment since LSCs may persist and potentially drive disease evolution from chronic phase (CP) to blast crisis (BC). Unfortunately, once patients have progressed to BC, allogeneic hematopoietic stem cell transplantation (alloHSCT) is the only potentially curative approach. The mechanisms of BC transformation are multifaceted and not fully elucidated.

MicroRNAs (miRNAs or miR) are short non-coding RNA molecules that downregulate target messenger (m) RNAs and in turn, the encoded proteins. Deregulated miRNAs in cancer, including leukemia, contribute to disease initiation and growth. *MIR142* is located at chromosome band 17q22 and is initially transcribed into a primary (pri)-miR-142 that eventually matures into miR-142-3p and miR-142-5p (hereafter collectively referred to as miR-142)^2^. MiR-142 reportedly regulates hematopoiesis^3^ and differentiation and activation of T lymphocytes, natural killer cells (NK) and dendritic cells (DC)^4–9^. Loss of the *mir142* gene in the mouse results in decreased hematopoietic output, and reduction of T, B and NK cells^4^. In humans, miR-142 mutations and/or downregulation have been found in lymphomas, acute lymphocytic leukemia (ALL) and acute myeloid leukemia (AML)^10–14^.

While miR-142 mutations have not commonly been found in CML and other myeloproliferative neoplasms (MPN)^15^, we recently reported that acquired miR-142 deficit induces BC transformation by promoting mitochondrial fusion and in turn enhancing levels of oxidative phosphorylation (OxPhos) in CP CML LSCs^16^. Treatment with a novel synthetic miR-142 mimic oligonucleotide (ODN; M-miR-142) corrected the miR-142 deficit, induced mitochondrial fission (i.e., fragmentation), and decreased OxPhos, thereby rescuing the BC phenotype^16^.

While studying the transforming role of miR-142 deficit, we observed reduced miR-142 levels and activity of mature T cells from BC CML patients compared with those from CP CML patients. Consistent with these results, we also discovered that, compared with the *Mir142*^*+/+*^*BCR-ABL* mouse [a CP CML model^17^], T cells in the *Mir142*^*−/−*^*BCR-ABL* mouse [a BC CML model^16^] had higher rate of spontaneous apoptosis and were significantly decreased. In this work, we report that acquired miR-142 deficit in T cells during CML BC transformation causes loss of T cells and LSC immune escape and promotes disease growth. Correcting miR-142 deficit with M-miR-142, alone or in combination with immune checkpoint antibody and/or TKI, restores T cell number and immune activity and prolongs survival of BC CML murine and patient-derived xenograft models.

## Results

### miR-142 deficit redirects LMPPs toward myeloid lineage and impairs T lymphoid differentiation

While studying the role of miR-142 deficit in CML evolution^16^, we observed that miR-142 was significantly decreased not only in CD34^+^CD38^-^ blasts^16^ but also in T cells from BC CML patients compared with CP CML patients (Supplementary Fig. 1a). No difference in miR-142 levels was instead observed between T cells from CP CML patients and those from healthy donors (Supplementary Fig. 1a). T cells in the peripheral blood (PB) mononuclear cell (MNC) samples procured from BC CML patients were also significantly lower than those from CP CML patients (Supplementary Fig. 1b). Accordingly, using genetically engineered mouse models (GEMMs), we observed that the BC *Mir142*^*−/−*^*BCR-ABL* mouse presented with significant T lymphopenia compared with the CP *Mir142*^*+/+*^*BCR-ABL* mouse (Supplementary Fig. 1c, d). Percentages and absolute numbers of CD4+ and CD8+ T cell subpopulations in PB, bone marrow (BM) and spleen of *Mir142*^*−/−*^*BCR-ABL* versus *Mir142*^*+/+*^*BCR-ABL* mice are shown in Supplementary Figs. 1e,f and 2. Thus, we hypothesized that an acquired miR-142 deficit induces loss of T cells and in turn decreased antileukemic immune activity during BC transformation.

**Fig. 1:**
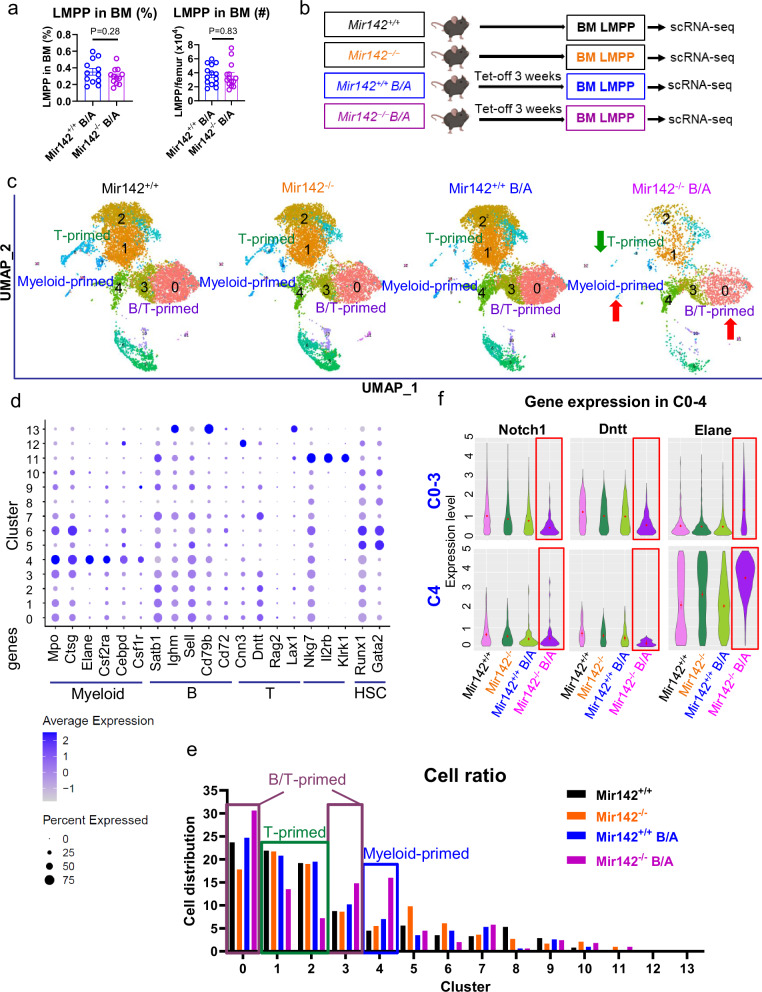
Single cell RNA-seq analysis of LMPPs derived from *Mir142*^*+/+*^*, Mir142*^*−/−*^*, Mir142*^*+/+*^*BCR-ABL*, and *Mir142*^*−/−*^*BCR-ABL* mice. **a** Frequency and number of LMPPs derived from BM of *Mir142*^*+/+*^*BCR-ABL* mice and *Mir142*^*−/−*^*BCR-ABL* mice (BCR-ABL were induced by tet-off for 3 weeks; *n* = 12 mice per group). **b**–**f** Schematic design and results. BM LMPP cells were sorted from *Mir142*^*+/+*^*, Mir142*^*−/−*^*, Mir142*^*+/+*^*BCR-ABL*, and *Mir142*^*−/−*^*BCR-ABL* mice and subjected to scRNA-seq (**b**). Fourteen clusters (C) including T-primed, Myeloid-primed, and B/T-primed clusters were identified (**c**) and expression levels of hematopoietic gene transcription factors and cluster differentiation (CD) antigens in different clusters are shown (**d**). Distribution of each cluster in LMPPs (**e**) and expression levels of *Notch1*, *Dntt*, and *Elane* genes in lymphoid-primed C0-3 and myeloid-primed C4 (**f**) are shown. *B/A* *BCR-ABL,* LMPP lymphoid-primed multipotent progenitors, tet tetracycline. For **a** comparison between groups was performed by two-tailed, unpaired *t*-test. Results shown represent mean ± SEM. Source data are provided as a Source Data file.

Lymphoid-primed multipotent progenitors [LMPPs, Lineage(Lin)^-^Sca-1^+^c-Kit^+^(LSK) CD150^-^FLT3^+^] derive from hematopoietic stem cells [HSCs, LSK CD150^+^CD48^-^ in mouse and Lin^-^CD34^+^CD38^-^CD90^+^ in human] and undergo thymic homing and differentiation to produce mature T cell progeny. Thus, to understand at which stage of hematopoiesis miR-142 deficit impairs T cell production, we first measured the frequency and number of BM LMPPs in leukemic *Mir142*^*−/−*^*BCR-ABL* mice and compared these results with the *Mir142*^*+/+*^*BCR-ABL* controls. BM LMPPs from the *Mir142*^*−/−*^*BCR-ABL* mice did not change in frequency and number compared with those from the *Mir142*^*+/+*^*BCR-ABL* mice (Fig. 1a), suggesting that miR-142 deficit did not decrease production of LMPPs, but rather impacted on their downstream differentiation.

Other than into T cells, LMPPs can differentiate into B, NK, dendritic and myeloid cells^18^. Thus, at baseline, it is expected that subsets of LMPPs dynamically express lineage-specific gene programs that eventually lead to distinct differentiation paths. To this end, we performed single cell (sc) RNA-seq analysis of LMPPs harvested from the BM of *Mir142*^*+/+*^*BCR-ABL* (CP CML; *n* = 3) and *Mir142*^*−/−*^*BCR-ABL* (BC CML; *n* = 3) mice (Fig. 1b). As controls, we included LMPPs from normal (non-leukemic) *Mir142*^+/+^ (wt, *n* = 5) and *Mir142*^−/−^ (miR-142 KO, *n* = 3) mice. Using the Louvain clustering algorithm to identify groups of cells with similar transcriptomes, we obtained 13 distinct clusters (C; Fig. 1c). We annotated these clusters into distinct lineage-primed subsets (Fig. 1c, d). Detailed annotation procedure is described in Methods. C1 and C2 had high levels of the T cell marker *Dntt* (Tdt) and low levels of the myeloid marker *Elane* and of the B cell marker *Cd79b*; these cells were therefore annotated as T-primed LMPP. C0 and C3 had high levels of *Dntt* and *Cd79b*, and low levels of *Elane* and therefore were annotated as B/T-primed. C4 had high levels of myeloid gene markers (i.e., *Elane*, *Csf1r*, *Csf2ra*, *Cebpd*, *Fes*) and low levels of *Dntt* and therefore was annotated as Myeloid-primed (Fig. 1c, d). Compared with *Mir142*^*+/+*^*, Mir142*^*−/−*^, or *Mir142*^*+/+*^*BCR-ABL* LMPPs, *Mir142*^*−/−*^*BCR-ABL* LMPPs presented with decreased C1 and C2 (T-primed) and increased C0, C3 (B/T-primed) and C4 (myeloid-primed). C0-C4 from *Mir142*^*−/−*^*BCR-ABL* LMPPs also presented with reduced expression of T cell gene markers (*Notch1* and *Dntt*) and increased expression of myeloid gene markers (*Elane*; Fig. 1e, f), suggesting that the concurrent presence of miR-142 deficit and BCR-ABL promoted the expansion of the myeloid-primed LMPP subset. Of note, consistent with reports that miR-142 deficit enhances oxidative metabolism^5,16^, we also observed enrichment of the hallmark gene sets involved in OxPhos in the scRNA-seq analysis comparing *Mir142*^*−/−*^*BCR-ABL* LMPPs with *Mir142*^*+/+*^*BCR-ABL* LMPPs (Supplementary Fig. 3). To assess the relevance of these findings to human disease, we then selected LMPPs (Lin^−^CD34^+^CD38^−^CD45RA^+^CD90^−^; Supplementary Fig. 4a) from three BC CML patients and three CP CML patients and performed scRNA-seq. Based on gene expression of reported myeloid, B, T and HSC markers^19,20^, C3, C4 and C11 (primarily expressing *CNN3* and *KLRG1*) were considered to be T-primed, C5 (expressing *MPO*, *LYZ*, *CSF1R*, *CTSG*, *AZU1*, *CEBPA*) myeloid-primed and C7 (expressing both B and T markers) B/T-primed LMPPs (Supplementary Figs. 4b and 5a). C8, although expressed *KLRG1*, did not express *CNN3* but expressed HSC markers (*HOPX*, *CRHBP*, *HLF* and *PCDH9*) and was deemed to represent more undifferentiated cells. Thus, LMPPs from BC CML patients also showed reduced T-primed subtypes (C3, C4 and C11) compared to LMPPs from CP CML patients (Supplementary Figs. 4b and 5b).

**Fig. 2:**
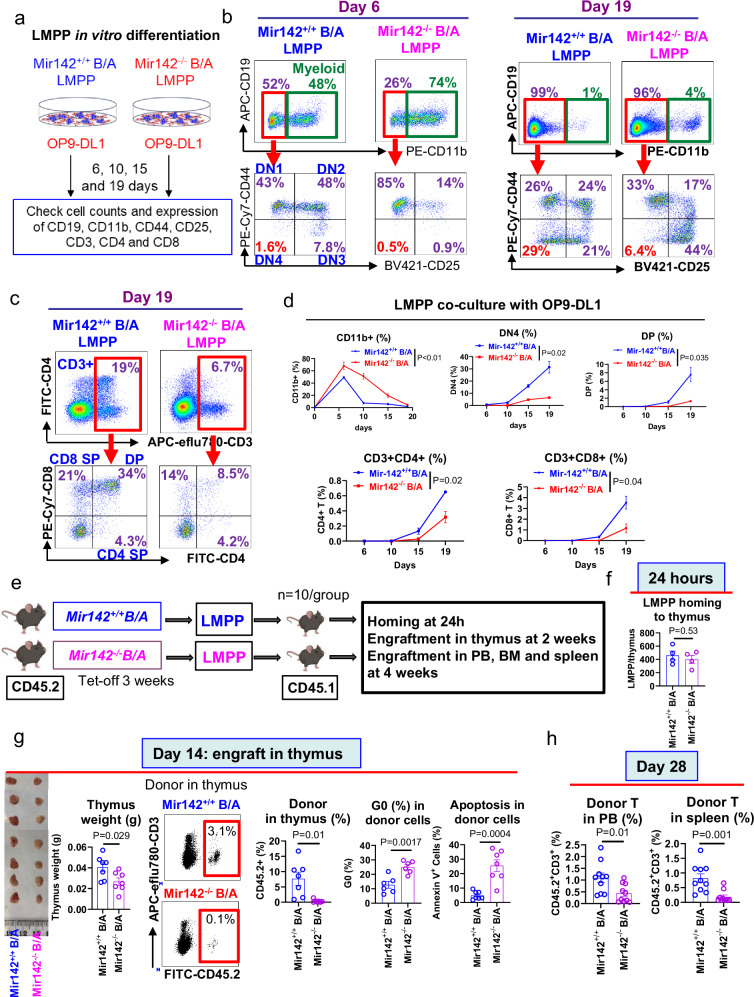
MiR-142 deficit redirects LMPPs toward myeloid lineage and impairs T lymphoid differentiation. **a**–**d**. Schematic design and results. BM LMPPs from *Mir142*^*+/+*^*BCR-ABL* and *Mir142*^*−/−*^*BCR-ABL* mice (BCR-ABL were induced by tet-off for 3 weeks) were co-cultured in vitro with OP9-DL1 cells for 19 days and B, Myeloid and T cell differentiation was analyzed on day 6, 10, 15 and 19 days (**a**). Representative plots of B (CD19^+^) and myeloid (CD11b^+^) lineage differentiation and T cell differentiation, i.e., CD4 and CD8 DN cells, on day 6 and day 19 (**b**), and CD4 and CD8 DP and SP cells on day 19 (**c**), and combined results showing percentages of CD11b^+^ myeloid cells, DN4, DP, and CD3^+^CD4^+^ and CD3^+^CD8^+^ mature T cells in LMPP-derived cells after co-culture with OP9-DL1 for 6, 10, 15 and 19 days (**d**), analyzed by flow cytometry. The experiments were repeated three times with similar results. **e**–**h** Experimental design and results. CD45.2 *Mir142*^*+/+*^*BCR-ABL* or *Mir142*^*−/−*^*BCR-ABL* LMPPs (30,000/mouse for homing experiment, 5,000/mouse for engraftment assessment) were transplanted into congenic CD45.1 normal wt recipients (**e**; *n* = 10 mice per group; mouse images created in BioRender. Chen, F. (2025) https://BioRender.com/e61c469). Numbers of LMPPs homing to thymus were measured at 24 h post injection by flow cytometry (**f**; *n* = 4 mice per group). On day 14 after LMPP transplantation, thymus was collected from the recipient mice and donor cell engraftment (*n* = 7 mice per group), cell cycling (*n* = 6 mice per group), and apoptosis (*n* = 7 mice per group) were measured by flow cytometry (**g**). On day 28, percentages of donor LMPP-derived T cells (CD45.2^+^CD3^+^) in PB and spleen were measured by flow cytometry (**h**; *n* = 10 mice per group). *B/A* *BCR-ABL,* LMPP lymphoid-primed multipotent progenitors, wt wild-type, BM bone marrow, PB peripheral blood, DN double negative, DP double positive, SP single positive. For **d** and **f**–**h**, comparison between groups was performed by two-tailed, unpaired *t*-test. Results shown represent mean ± SEM. Source data are provided as a Source Data file.

To test if miR-142 deficit impaired LMPP T-lymphoid differentiation, we then co-cultured LMPPs from *Mir142*^*−/−*^*BCR-ABL* and *Mir142*^*+/+*^*BCR-ABL* mice with OP9-DL1 cells (Fig. 2a). OP9-DL1 is a BM-derived stromal cell line that ectopically expresses the Notch ligand, Delta-like 1 and promotes in vitro T-lymphocyte differentiation. On day 6 of co-culture, we observed 74% cells with myeloid markers in *Mir142*^*−/−*^*BCR-ABL* LMPP-derived cells versus (vs) 48% in *Mir142*^*+/+*^*BCR-ABL* LMPPs-derived cells (Fig. 2b, d), supporting our initial observation that miR-142 deficit initially redirected LMPP toward myeloid differentiation. On day 19, the number of mature T lymphocytes derived from *Mir142*^*−/−*^*BCR-ABL* LMPPs was significantly reduced compared with that derived from *Mir142*^*+/+*^*BCR-ABL* LMPPs (Fig. 2c, d). T cell thymic differentiation normally occurs through the following maturation steps: CD4-CD8- (double negative, DN), CD4+ CD8+ (double positive, DP) and CD4+ or CD8+ (single positive, SP) T-lymphocytes. Gating on non-myeloid cells from *Mir142*^*−/−*^*BCR-ABL* LMPPs, we observed a significant delay in lymphoid differentiation from DN1 (CD25-CD44+) to DN2 (CD25+ CD44+), DN3 (CD25+ CD44-) and DN4 (CD25-CD44-) both at day 6 and 19 (Fig. 2b, d) compared with cells from *Mir142*^*+/+*^*BCR-ABL* LMPPs. A significant reduction of DP and SP cells deriving from *Mir142*^*−/−*^*BCR-ABL* LMPPs was also observed on day 19 (Fig. 2c, d). These results suggest that miR-142 deficit impaired LMPP T cell differentiation.

To confirm these observations in vivo, we assessed LMPP thymic homing and differentiation by transplanting BM CD45.2 *Mir142*^*−/−*^*BCR-ABL* and *Mir142*^*+/+*^*BCR-ABL* LMPPs into normal wt CD45.1 recipients (Fig. 2e). While we observed no significant changes in thymic homing of *Mir142*^*−/−*^*BCR-ABL* LMPP at 24 h (Fig. 2f), on day 14 we noticed that compared with *Mir142*^*+/+*^*BCR-ABL* LMPP recipients, the *Mir142*^*−/−*^*BCR-ABL* LMPP recipients had smaller thymi and fewer thymic donor-derived (CD45.2^+^) cells, which were arrested at G0 and prone to spontaneous apoptosis (Fig. 2g). Of note, analysis of the thymic CD45.2^+^ donor cells revealed increased frequency of DN1 and DN2 (the early stages of T cell development) and decreased numbers of DP, CD4^+^ and CD8^+^ SP cells in the *Mir142*^*−/−*^*BCR-ABL* LMPP recipients compared with the *Mir142*^*+/+*^*BCR-ABL* LMPP recipients (Supplementary Fig. 6a, upper, left panel). By day 28, the donor-derived circulating and splenic T cells (CD45.2^+^CD3^+^), including both CD4^+^ T and CD8^+^ T cells, were reduced in the *Mir142*^*−/−*^*BCR-ABL* LMPP recipients compared with the *Mir142*^*+/+*^*BCR-ABL* LMPP recipients (Fig. 2h and Supplementary Fig. 6a, upper, right panel). Of note, in the *Mir142*^*−/−*^*BCR-ABL* LMPP recipients, αβ T cells, rather than γδT cells, were significantly reduced compared to the counterparts in the *Mir142*^*+/+*^*BCR-ABL* LMPP recipients (Supplementary Fig. 6a, lower panel). Similar changes were also observed when CD45.2 LMPPs from *Mir142*^*−/−*^ *or Mir142*^*+/+*^ mice were transplanted into CD45.1 normal wt recipients (Supplementary Fig. 6b), suggesting that they were caused by the miR-142 deficit itself, rather than BCR-ABL expression.

### miR-142 deficit impairs in vitro antileukemic T cell activity

**Fig. 3:**
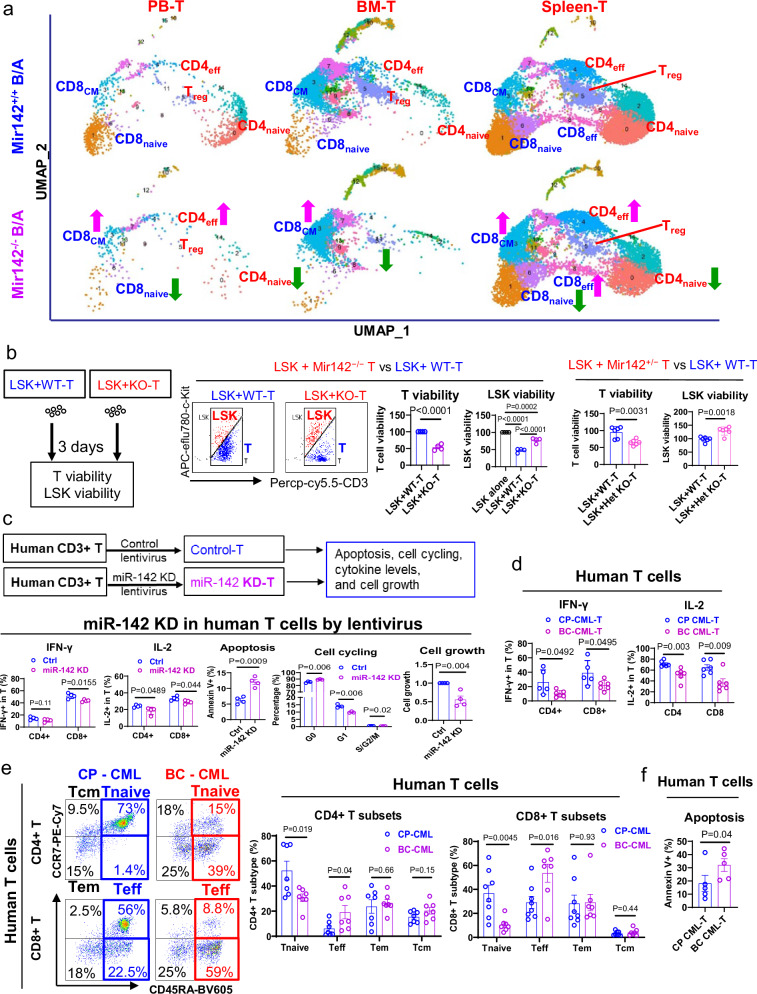
MiR-142 deficit impairs T cell in vitro antileukemic activity. **a** PB, BM and spleen T cells from *Mir142*^*+/+*^*BCR-ABL* and *Mir142*^*−/−*^*BCR-ABL* mice (BCR-ABL were induced by tet-off for 3 weeks) were subjected to scRNA-seq. Fifteen clusters were identified and annotated into distinct T cell subsets based on expression levels of naïve, memory, effector, and regulatory T gene markers. **b** Schematic design of experiment and results. LSKs from *Mir142*^*−/−*^*BCR-ABL* mice were co-cultured with *Mir142*^*+/+*^ T (WT-T) or *Mir142*^*−/−*^ T (homozygous KO; *n* = 4 samples per group; middle panel), or with WT-T or *Mir142*^*+/−*^ T (heterozygous KO; *n* = 6 samples per group; right panel) cells for 3 days, then viability of T and LSK cells was determined. **c** Cytokine levels of IFN-γ (*n* = 5 samples per group) and IL-2 (*n* = 4 per group), apoptosis (*n* = 4 per group), cell cycling (*n* = 3 per group), and cell growth (*n* = 4 per group) of human T cells from healthy donors with or without miR-142 KD were shown. **d** Levels of IFN-γ (*n* = 5 samples for CP CML and *n* = 6 for BC CML) and IL-2 (*n* = 6 samples per group) production in T cell subpopulations from CP CML or BC CML patients were shown. **e** Representative plots (left) and combined results (right) of CD4^+^ (*n* = 7 samples per group) and CD8^+^ (*n* = 8 for CP CML; *n* = 7 for BC CML) subpopulations in T cells from patients with BC CML or CP CML. **f** Spontaneous apoptosis of T cells from CP CML or BC CML patients (*n* = 5 samples per group). *B/A* *BCR-ABL,* PB peripheral blood, BM bone marrow, tet tetracycline, scRNA-seq single cell RNA sequencing, CM central memory, EM effector memory, Treg regulatory T, KD knock down, CML chronic myeloid leukemia, CP chronic phase, BC blast crisis. For **b**–**f**, comparison between two groups was performed by two-tailed, unpaired *t*-test. For **b**, comparisons among multi-groups were performed by one-way ANOVA and *P* values were corrected for multiple comparisons using Holm–Šídák method. Results shown represent mean ± SEM. Source data are provided as a Source Data file.

Next, to assess the subpopulation distribution and activity of the fewer mature T cells that emerged from the impaired LMPP differentiation in the *Mir142*^*−/−*^*BCR-ABL* mouse, peripheral blood (PB) mononuclear cells (MNCs) and BM and splenic T cells (CD3^+^) were isolated from *Mir142*^*−/−*^*BCR-ABL, Mir142*^*+/+*^*BCR-ABL, Mir142*^*−/−*^ *and Mir142*^*+/+*^ mice and subjected to scRNA-seq. Using the Louvain clustering algorithm, we identified 16 distinct clusters (Fig. 3a) in T cells from PB, BM and spleen and annotated them into distinct T cell subsets based on their expression levels of naïve, memory, effector and regulatory T gene markers and cluster differentiation (CD) antigens (Supplementary Table 1). The annotation procedure is detailed in Methods. Compared with the T cells from *Mir142*^*+/+*^*BCR-ABL* mouse, the T cells from *Mir142*^*−/−*^*BCR-ABL* mouse presented with decreases in naïve T and regulatory T (Treg) cells and enrichment in effector T (Teff) and CD8+ central memory T (Tcm) cells (Fig. 3a, Supplementary Fig. 7a) which also seemingly expressed higher levels of exhaustion markers^21,22^ (Supplementary Fig. 7b). These results have been confirmed by immunophenotypic analysis (Supplementary Fig. 7c, d). T cells from the *Mir142*^*−/−*^*BCR-ABL* mouse seemingly produced less cytokines and cytotoxicity associated proteins (i.e., IFNγ, IL2, Granzyme B, and CD107a), arrested in G0, and had increased rate of spontaneous apoptosis compared with those from the *Mir142*^*+/+*^*BCR-ABL* mouse (Supplementary Fig. 8a–c). Of note, T cells from the *Mir142*^*+/−*^*BCR-ABL* mouse also produced less cytokines (e.g., IFNγ and IL2) and had increased spontaneous apoptosis rate compared with those from the *Mir142*^*+/+*^*BCR-ABL* mouse (Supplementary Fig. 8d). A significantly higher percentage of *Mir142*^*−/−*^*BCR-ABL* LSKs remained viable after co-culture with either *Mir142*^*−/−*^ T cells or *Mir142*^*+/−*^ T cells compared with *Mir142*^*−/−*^*BCR-ABL* LSKs co-cultured with *Mir142*^*+/+*^ T cells (Fig. 3b), suggesting decreased antileukemic activity of T cells even with only partial miR-142 deficit.

Of note, decreased cytokine production (i.e., IFNγ and IL-2) and increased spontaneous apoptosis and cell cycle arrest were also observed in human T cells from healthy donors transduced with a miRZip anti-miR-142 lentiviral vector compared with those transduced with a miRZip null-vector (Fig. 3c), and in human T cells from healthy donors treated with miR-142 inhibitor (2 μM anti-miR-142-3p/5p) compared with control cells treated with scramble RNA (SCR) (Supplementary Fig. 9a–d). To this end, T cells from BC CML patients had, in addition to lower miR-142 levels (Supplementary Fig. 1a), reduced cytokine production (i.e., IFNγ and IL-2; Fig. 3d), reduced frequencies of naïve T cells (CD3^+^ CD45RA^+^ CCR7^+^), increased frequencies of effector T cells (CD3^+^ CD45RA^+^ CCR7^−^) (Fig. 3e), and increased rates of spontaneous apoptosis [mainly in effector and effector memory (CD3^+^ CD45RA^−^CCR7^−^) T subtypes; Fig. 3f; Supplementary Fig. 9e], compared with T cells isolated from CP CML patients.

### miR-142 deficit impairs T cells’ metabolic switch in BC CML

We have previously reported that miR-142 deficit enhances mitochondrial fusion and OxPhos^16^. Dynamic metabolic changes in levels of OxPhos and glycolysis play a key role in T cell activation^23,24^. MiR-142 has been previously reported to be involved in regulating T cell responses^6^. We observed that in addition to gene sets involved in apoptosis, Teff cells from the *Mir142*^*−/−*^*BCR-ABL* mouse showed enrichment in the expressed genes involved in OxPhos compared with Teff cells from *Mir142*^*+/+*^*BCR-ABL* mouse (Supplementary Fig. 10). Thus, to gain further mechanistic insight, we conducted an unbiased metabolomic profiling of freshly isolated (resting) and activated (cultured in the presence of activating anti-CD3/CD28 antibody-coated beads for 24 h) T cells from either *Mir142*^*−/−*^*BCR-ABL* or *Mir142*^*+/+*^*BCR-ABL* mice.

We identified a total of 216 non-redundant endogenous metabolites that were differentially abundant in resting and activated T cells from *Mir142*^*−/−*^*BCR-ABL* and *Mir142*^*+/+*^*BCR-ABL* mice (Supplementary Fig. 11a; Supplementary Data 1). Most acyl carnitines, fatty acids and derivatives, and lipids were lower, and nucleotides and their metabolites, organic acids, sugars and their metabolites were higher in *Mir142*^*−/−*^*BCR-ABL* T cells compared to *Mir142*^*+/+*^*BCR-ABL* T cells (Supplementary Data 1). Enhanced OxPhos activity in resting *Mir142*^*−/−*^*BCR-ABL* T cells versus resting *Mir142*^*+/+*^*BCR-ABL* T cells was supported by changes of metabolites involved in energy pathways, i.e., increased citrate/pyruvate ratio and higher levels of fumarate, citrate, and oxaloacetate (Supplementary Fig. 11b; Supplementary Data 2 and 3). Upon activation, while OxPhos and glycolysis metabolites increased in *Mir142*^*+/+*^*BCR-ABL* T cells relative to their resting controls, they did not increase in activated *Mir142*^*−/−*^*BCR-ABL* T cells vs resting *Mir142*^*−/−*^*BCR-ABL* T cells (Supplementary Fig. 11b, c; Supplementary Data 2 and 3), suggesting an impaired metabolic reprogramming during T cells’ activation in the *Mir142*^*−/−*^*BCR-ABL* BC mouse.

To confirm these observations, we performed Agilent Seahorse functional assays. At baseline, we observed a significant increase in OxPhos [reported as oxygen consumption rate (OCR)] and not in glycolysis [reported as extracellular acidification rate (ECAR)] upon FCCP stimulation in *Mir142*^*−/−*^*BCR-ABL* T cells compared with *Mir142*^*+/+*^*BCR-ABL* T cells (Supplementary Fig. 11d). Upon activation with anti-CD3/CD28 antibodies, OxPhos failed to increase in FCCP-stimulated *Mir142*^*−/−*^*BCR-ABL* T cells, while increased in *Mir142*^*+/+*^*BCR-ABL* T cells; glycolysis increased upon FCCP stimulation but at a significantly lower degree in *Mir142*^*−/−*^*BCR-ABL* T cells than in *Mir142*^*+/+*^*BCR-ABL* T cells (Supplementary Fig. 11d). Thus, when taken altogether, these results suggest that miR-142 deficit impaired the metabolism shift that supports T cell activation and expansion.

### Lack of miR-142 significantly decreases in vivo T cells’ antileukemic activity

**Fig. 4:**
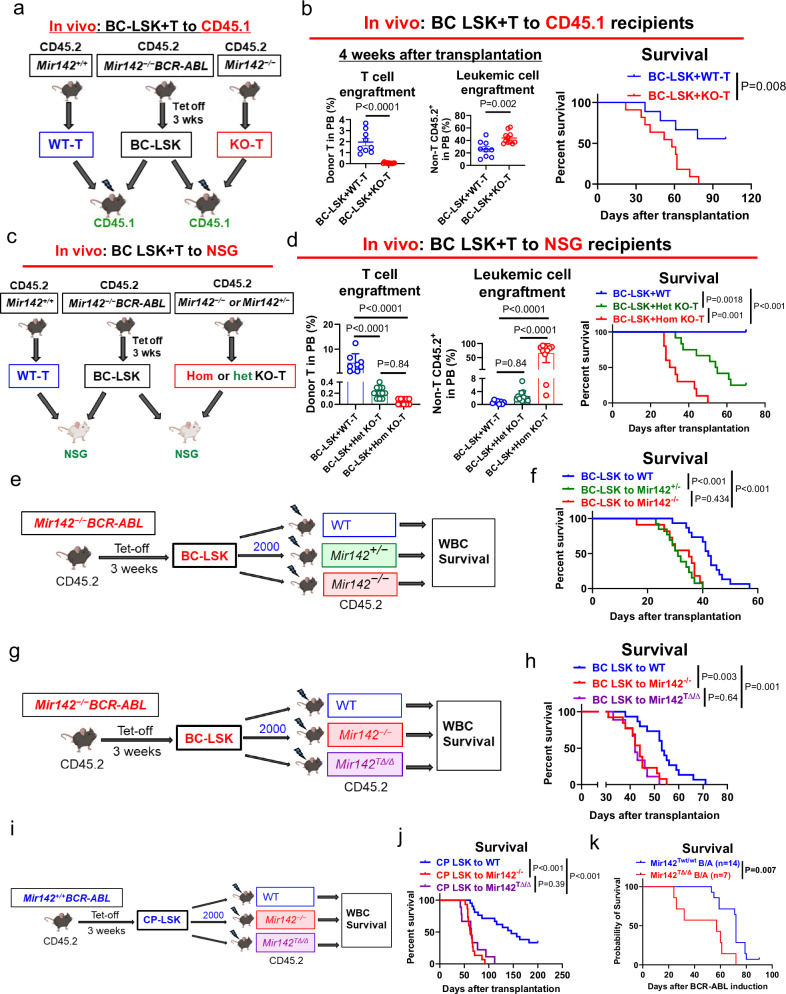
MiR-142 deficit decreases T cell in vivo antileukemic activity. **a**, **b** Experimental design and results. CD45.2 *Mir142*^*−/−*^*BCR-ABL* LSK (BC-LSK) and *Mir142*^*+/+*^ T (WT-T) or *Mir142*^*−/−*^ T (KO-T) cells were co-transplanted into CD45.1 congenic recipients (lethally irradiated to eradicate host T cells; **a**). Donor T cell (CD45.2^+^ CD3^+^) and leukemic cell (% of CD45.2^+^ minus % of CD45.2^+^ CD3^+^) engraftment in PB at 4 weeks after transplantation and survival of the recipients were analyzed (*n* = 9 mice for BC-LSK + WT-T group; *n* = 11 for BC-LSK+ KO-T group; **b**). **c**, **d**. Experimental design and results. CD45.2 *Mir142*^*−/−*^*BCR-ABL* LSKs were co-transplanted with *Mir142*^*+/+*^ or *Mir142*^*+/−*^ or *Mir142*^*−/−*^ T cells into NSG mice (CD45.1, lacking T cells; **c**). Donor T cell and leukemic cell engraftment in PB at 4 weeks after transplantation and survival of the recipients were analyzed (**d**; *n* = 8 mice for BC-LSK+ WT-T group; *n* = 10 for BC-LSK+hom KO-T group; *n* = 12 for BC-LSK+het KO-T group). Experimental design and results. *Mir142*^*−/−*^*BCR-ABL* LSKs were transplanted into *Mir142*^*+/+*^ (*n* = 15), *Mir142*^*+/−*^(*n* = 13), or *Mir142*^*−/−*^(*n* = 11) recipients (**e**) and survival of the recipients was analyzed (**f**). Experimental design and results. *Mir142*^*−/−*^*BCR-ABL* LSKs were transplanted into *Mir142*^*+/+*^ (*n* = 15), *Mir142*^*−/−*^(*n* = 13), or *Mir142*^*flox(f)/f*^*Lck-cre*+ (*Mir142*^*TΔ/Δ*^, *n* = 9) recipients (**g**) and survival of the recipients was analyzed (**h**). Experimental design and results. *Mir142*^*+/+*^*BCR-ABL* LSKs (CP-LSK) were transplanted into *Mir142*^*+/+*^ (*n* = 21), *Mir142*^*−/−*^ (*n* = 15), or *Mir142*^*TΔ/Δ*^ (*n* = 9) recipients (**i**) and survival of the recipients was analyzed (**j**). **k** Survival of *Mir142*^*flox(f)/f*^*Lck-cre- BCR-ABL* (*Mir142*^*Twt/wt*^ B/A, *n* = 14) and *Mir142*^*f/f*^*Lck-cre*+ *BCR-ABL* (*Mir142*^*TΔ/Δ*^ B/A, *n* = 7) mice. CP chronic phase, BC blast crisis, PB peripheral blood, tet tetracycline, LSK Lin^−^Sca-1^+^c-Kit^+^, w week. For **b**, comparison between two groups was performed by two-tailed, unpaired *t*-test. For **d**, comparisons among multi-groups were performed by one-way ANOVA. For overall survival data in **b**, **d**, **f**, **h**, **j** and **k**, log-rank test was used to compare two or more survival curves. *P* values were corrected for multiple comparisons using Holm–Šídák method. Results shown represent mean ± SEM. For **a**, **c**, **e**, **g** and **i**, mouse images were created in BioRender. Chen, F. (2025) https://BioRender.com/e61c469. Source data are provided as a Source Data file.

To assess in vivo the T cell antileukemic activity in BC CML, we co-transplanted *Mir142*^*−/−*^*BCR-ABL* LSK (CD45.2, 5000 cells) and *Mir142*^*+/+*^ T or *Mir142*^*−/−*^ T cells (CD45.2, 10^6^ cells) into CD45.1 congenic recipients (lethally irradiated to eradicate host T cells; Fig. 4a). Four weeks after transplantation, we observed significant reduction of circulating T cells (CD45.2^+^CD3^+^: 0.07% vs 1.73%, *P* < 0.0001) and increase of circulating leukemic blasts (CD45.2^+^ minus CD45.2^+^CD3^+^: 45% vs 28%, *P* = 0.002) in LSK/*Mir142*^*−/−*^ T recipients compared with LSK/*Mir142*^*+/+*^ T recipients (Fig. 4b). The LSK/*Mir142*^*−/−*^ T recipients also had shorter survival (median: 58 vs unreached after monitoring for 100 days, *P* = 0.008) compared to the LSK/*Mir142*^*+/+*^ T recipients (Fig. 4b).

To eliminate the potential impact of the recipient mouse’s “endogenous” miR-142 wt T cells and to evaluate if even only a partial deficit of miR-142 as that observed in human T cells from BC CML patients could lead to reduced T cell-mediated antileukemic activity, we repeated the experiment by transplanting immunodeficient NSG recipients (CD45.1) that lack their own T cells, with *Mir142*^*−/−*^*BCR-ABL* LSKs (CD45.2, 10^4^ cells/mouse) and *Mir142*^*−/−*^, *Mir142*^*+/−*^ or *Mir142*^*+/+*^ T cells (CD45.2, 10^6^ cells/mouse) selected from non-leukemic donors with the respective genotypes (Fig. 4c). Four weeks after transplantation, LSK/*Mir142*^*−/−*^ T recipients showed decrease of circulating T cells compared with LSK/*Mir142*^*+/+*^ T recipients, increase of circulating leukemic blasts and shorter survival (median: 31.5 vs 54.5 vs unreached, LSK/*Mir142*^*−/−*^ T vs LSK/*Mir142*^*+/−*^ T: *P* = 0.001; LSK/*Mir142*^*−/−*^ T vs LSK/*Mir142*^*+/+*^ T: *P* < 0.001) compared with LSK/*Mir142*^*+/−*^ T and LSK/*Mir142*^*+/+*^ T recipients (Fig. 4d). Of note, LSK/*Mir142*^*+/−*^ T recipients also showed decrease of circulating T cells (CD45.2^+^CD3^+^: 0.22% vs 4.48%, *P* < 0.0001), and shorter survival (median: 54.5 days vs unreached, *P* = 0.0018) compared to LSK/*Mir142*^*+/+*^ T recipients (Fig. 4d). Next, we transplanted BC CML LSCs (LSKs) into *Mir142*^*−/−*^, *Mir142*^*+/−*^, or *Mir142*^*+/+*^ recipient mice (Fig. 4e). *Mir142*^*−/−*^ and *Mir142*^*+/−*^ recipients had similarly shorter survival (median: 35 vs 31 days, *P* = 0.434), than *Mir142*^*+/+*^ recipients (median: 42 days; *Mir142*^*−/−*^ vs *Mir142*^*+/+*^: *P* < 0.001; *Mir142*^*+/−*^ vs *Mir142*^*+/+*^: *P* < 0.001; Fig. 4f).

To compartmentalize the impact of miR-142 deficit on T cells and separate it from that on LSKs, we then transplanted *Mir142*^*−/−*^*BCR-ABL* LSKs into normal *Mir142*^*−/−*^, *Mir142*^*flox(f)/f*^*Lck-cre*^+^ (i.e., conditional miR-142 KO in T cells only; *Mir142*^TΔ/Δ^), or *Mir142*^*+/+*^ recipients (Fig. 4g). *Mir142*^*−/−*^ and *Mir142*^TΔ/Δ^ recipients had a similar survival (median: 44 vs 42 days, *P* = 0.64), and both had a significantly shorter survival than the *Mir142*^*+/+*^ recipients (median: *Mir142*^*−/−*^ vs *Mir142*^*+/+*^ recipients: 44 vs 53 days, *P* = 0.003; *Mir142*^TΔ/Δ^ vs *Mir142*^*+/+*^ recipients: 42 vs 53 days, *P* = 0.001; Fig. 4h). Of note, similar results were also observed in normal *Mir142*^*−/−*^, *Mir142*^TΔ/Δ^ or *Mir142*^*+/+*^ recipients transplanted with *Mir142*^*+/+*^*BCR-ABL* LSKs. *Mir142*^*−/−*^ and *Mir142*^TΔ/Δ^ recipients had a shorter survival than *Mir142*^*+/+*^ recipient controls (median: *Mir142*^*−/−*^ vs *Mir142*^TΔ/Δ^ vs *Mir142*^*+/+*^ recipients: 62 vs 64 vs 157 days; *Mir142*^*−/−*^ vs *Mir142*^*+/+*^ recipients: *P* < 0.001; *Mir142*^TΔ/Δ^ vs *Mir142*^*+/+*^ recipients: *P* < 0.001; Fig. 4i, j). Finally, we generated *Mir142*^*f/f*^*Lck-cre*^+^*/BCR-ABL* (with T-specific miR-142 KO, hereafter indicated as *Mir142*^*TΔ/Δ*^*BCR-ABL*) mice and found that *Mir142*^*TΔ/Δ*^*BCR-ABL* mice had a significantly shorter survival than the Cre- (*Mir142*^*Twt/wt*^*BCR-ABL*) controls (median: 57 vs 72 days, *P* = 0.007; Fig. 4k). Taken altogether, these results support that miR-142 deficit negatively impacts on T cell-mediated antileukemic activity likely by inducing T lymphopenia, although a functional impact of miR-142 loss on T cell activity could also be possible.

### Cytokines mediate T cells’ miR-142 deficit in BC CML

**Fig. 5:**
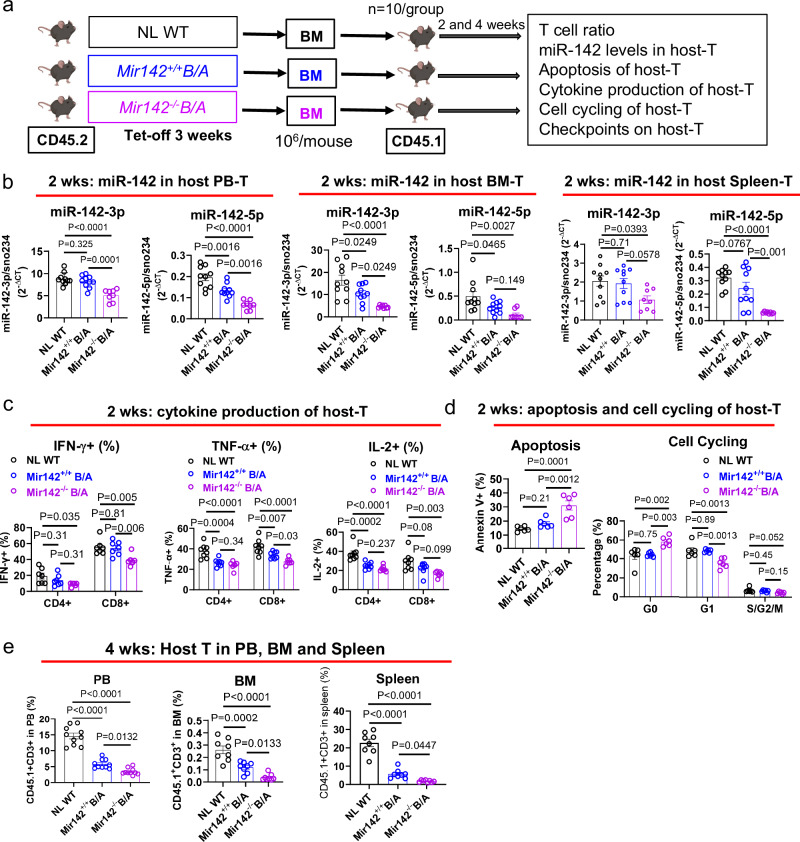
T cell miR-142 deficit in BC CML is mediated by blast-secreted cytokines. **a**–**e** Experimental design and results. BM MNCs from CD45.2 normal wt, *Mir142*^*+/+*^*BCR-ABL* and *Mir142*^*−/−*^*BCR-ABL* mice were transplanted into congenic CD45.1 recipients (**a**; 10^6^/mouse, *n* = 10 mice per group; mouse images created in BioRender. Chen, F. (2025) https://BioRender.com/e61c469). miR-142 levels in host T cells (CD45.1^+^ CD3^+^) from PB, BM and spleen of the recipients was determined by Q-RT-PCR at 2 weeks after transplantation (**b**; *n* = 10 mice for normal wt and *Mir142*^*+/+*^*BCR-ABL* groups; *n* = 8 for *Mir142*^*−/−*^*BCR-ABL* group). Cytokine production (**c**) of IFN-γ (*n* = 7 mice per group), TNF-α (*n* = 8 mice per group), and IL-2 (*n* = 8 mice per group) and apoptosis and cell cycling (**d**; *n* = 6 mice per group) of host T cells from the spleen of the recipients at 2 weeks after transplantation, and percentages of host T cells in PB (*n* = 10 mice per group), BM (*n* = 8 mice per group) and spleen (*n* = 15 mice per group) at 4 weeks after transplantation (**e**) were analyzed by flow cytometry. wks weeks, BM bone marrow, MNC mononuclear cells, NL WT normal wild-type, *B/A* *BCR-ABL,* tet tetracycline, PB peripheral blood. For **b**–**e**, comparisons among multi-groups were performed by one-way ANOVA. *P* values were corrected for multiple comparisons using Holm–Šídák test. Results shown represent mean ± SEM. Source data are provided as a Source Data file.

While GEMMs are useful to understand the mechanistic role of miR-142 deficit in T cell differentiation and activity during BC transformation, they do not explain how miR-142 deficit is acquired in T cells from BC patients. To this end, we transplanted BM MNCs from *Mir142*^*−/−*^*BCR-ABL, Mir142*^*+/+*^*BCR-ABL*, or normal wt mice (CD45.2) into congenic normal wt recipients (CD45.1, 10^6^/mouse; Fig. 5a) and measured miR-142 levels in the host T cells. Two weeks after transplantation, we observed that CD45.1^+^CD3^+^ T cells from the recipients of *Mir142*^*−/−*^*BCR-ABL* BM MNCs had lower miR-142 levels, reduced cytokine production, increased spontaneous apoptosis and cell cycle arrest compared with those from the recipients of *Mir142*^*+/+*^*BCR-ABL* or normal wt BM MNCs (Fig. 5b–d and Supplementary Fig. 12a–c). Four weeks after transplantation, we observed a significant reduction of host T cells in the recipients of *Mir142*^*−/−*^*BCR-ABL* BM MNCs compared with the recipients of *Mir142*^*+/+*^*BCR-ABL* and normal wt BM MNCs (Fig. 5e). Similar results were also observed when T cells were co-cultured with BM MNCs from *Mir142*^*−/−*^*BCR-ABL* mice compared with those co-cultured with BM MNCs from *Mir142*^*+/+*^*BCR-ABL* or normal wt mice, in a transwell plate that separated these two cell populations (Supplementary Fig. 12d). Of note, we also observed reduced miR-142 levels and cytokine production in human T cells co-cultured with BM MNCs from BC CML patients compared with those co-cultured with BM MNCs from CP CML patients and healthy donors (Supplementary Fig. 12e). Thus, we postulated that reduction of miR-142 levels in T cells could be induced by secreted factors during disease growth and evolution.

Using a Luminex assay, we then compared PB, BM and spleen cytokines levels (Supplementary Fig. 12f). Treatments of normal wt T cells with cytokines that were found significantly higher in the BC mouse, narrowed down to IL-6 as one of the main cytokines that downregulated miR-142 (Supplementary Fig. 12g, h). Of note, IL-6 has been previously reported to be significantly higher in the serum of BC CML patients compared with CP CML patients^25^, and has been also linked to miR-142 downregulation^26^. We also observed increased IL-6 expression in BC CML PB MNCs compared to CP CML PB MNCs or normal PB MNCs (Supplementary Fig. 12i). Accordingly, we showed in vitro reduction of miR-142 levels in LMPPs from the *Mir142*^*+/+*^*BCR-ABL* mouse co-cultured with OP9-DL1 cells in the presence of IL-6 (Supplementary Fig. 13a), which also promoted myeloid differentiation and impaired T cell differentiation (Supplementary Fig. 13b–h). On day 6 of co-cultures, we detected 62% myeloid cells (CD11b+) in IL-6-treated LMPPs vs 48% in vehicle (PBS)-treated LMPPs (*p* = 0.0006; Supplementary Fig. 13b, e); on day 10, we observed 14% myeloid cells in IL-6-treated LMPPs vs 7% in PBS-treated LMPPs (*p* = 0.0007; Supplementary Fig. 13c, e). Gating on non-myeloid cells (38% vs 52% at day 6), we observed a delay in lymphoid differentiation at the early stage of T cell development, i.e., DN1 to DN2 and DN3 on day 6 (55%, 34%, 5.9% vs 43%, 48%, 7.8%, respectively; Supplementary Fig. 13b) and to DN4 on day 19 (8% vs 29%; Supplementary Fig. 13d, f) in IL-6-treated vs PBS-treated LMPP co-cultures. On day 19 of the OP9-DL1 co-culture system, a significant reduction in the late stage of T cell development, i.e., DP (0.199% vs 7.59%, *p* = 0.03) and CD4+ (0.29% vs 0.65%, *p* = 0.0007) and CD8^+^ (1.1% vs 3.5%, *p* = 0.04) mature T cells, was also observed in the IL-6-treated LMPPs compared with the PBS-treated LMPPs (Supplementary Fig. 13d, g, h). These results suggest that IL-6 may play a role in redirecting LMPPs to myeloid differentiation through downregulating miR-142.

Of note, we also observed an increase of miR-142 levels in T cells from normal wt mice or from healthy donors that were co-cultured respectively with BM MNCs from *Mir142*^*−/−*^*BCR-ABL* mice or from BC CML patients and treated with anti-IL-6 blocking antibody (Ab) compared with those treated with IgG (Supplementary Fig. 13i, j). Furthermore, to assess if leukemia-induced decrease in miR-142 levels and activity of T cells could be rescued by blocking IL-6, we transplanted BM cells from *Mir142*^*−/−*^*BCR-ABL* mice (CD45.2) into congenic normal wt recipients (CD45.1, 10^6^/mouse). Starting on Day 3 after transplant, we treated these mice with IL-6 blocking Ab (100 µg, ip, 3 times per week) or IgG for 2 weeks (Supplementary Fig. 13k). We observed increased miR-142 levels in the CD45.1^+^CD3^+^ host T cells from the IL-6 Ab-treated mice compared with those from the IgG-treated controls (Supplementary Fig. 13l). The IL-6 Ab-treated mice also showed an increase of host T cells in PB at 4 weeks post transplantation and survived longer than the IgG-treated controls (Supplementary Fig. 13m, n). Taken altogether, these data support that IL-6 can impair both T cell differentiation and immune activity by downregulating miR-142.

### Upregulation of the PD-1/PD-L1 axis during BC transformation

Programmed cell death protein 1 (PD-1) is a marker for T cell activation and, if accompanied by other indicators of reduced T cell activity, for possible exhaustion^27^. We noticed that T cells from *Mir142*^*−/−*^*BCR-ABL* mice had an increase in PD-1 expression (Supplementary Fig. 14a–d). In addition to the aforementioned reduction of cytokine production, cell cycling and anti-leukemic activity and increase of apoptosis rate (Fig. 3b; Supplementary Fig. 8a–d), increased levels of other immune checkpoints (e.g., Tim-3 and CTLA-4) were also observed in the CD4^+^ and CD8^+^ spleen T cells from the *Mir142*^*−/−*^*BCR-ABL* mouse compared to those from the *Mir142*^*+/+*^*BCR-ABL* mouse (Supplementary Fig. 14e, f), suggesting T cell exhaustion in the BC mouse.

The causative impact of miR-142 deficit on PD-1 expression was supported by the increase in PD-1 expression in normal wt T cells treated with a miR-142 inhibitor (2 μM anti-miR-142-3p/5p) compared to SCR-treated controls (Supplementary Fig. 14g, h). Conversely, treatment with a synthetic miR-142 mimic compound [i.e., 2μΜ miR-142-3p and -5p mimics hereafter called collectively M-miR-142^16^] reduced both in vitro and in vivo PD-1 expression and spontaneous apoptosis rate (Supplementary Figs. 14i–k, 15a), and increased proliferation and cytokine production (IFNγ; Supplementary Fig. 15b–d) of *Mir142*^*−/−*^*BCR-ABL* T cells. Of note, concurrently with the increase in T cells’ PD-1 expression, we also noticed increased expression of the programmed cell death ligand 1 (PD-L1) on LSKs (Supplementary Fig. 15e) from *Mir142*^*−/−*^*BCR-ABL* mice compared with those from *Mir142*^*+/+*^*BCR-ABL* or normal wt mice, which was partly rescued by treatment with M-miR-142 (2 μΜ; Supplementary Fig. 15f). The relevance of these findings to human disease was supported by the observation of reduced miR-142 levels (Supplementary Fig. 1a) and increased PDCD1 (PD-1) and CTLA-4 mRNA expression (by RT-PCR; Supplementary Fig. 15g), along with increased membrane surface levels of PD-1 and TIM-3 proteins (by flow cytometry analysis; Supplementary Fig. 15h–j), in T cells from BC CML patients compared with those from CP CML patients. Like in the mouse, CD4^+^ and CD8^+^ effector and effector memory T cells from BC CML patients had significantly increased PD-1 levels compared to their counterparts from CP CML patients (Supplementary Fig. 15j).

To test further the interplay between miR-142 deficit and PD-1 expression in T cells and understand if the latter may be indicative also of T cell exhaustion, we isolated LSKs from *Mir142*^*+/+*^*BCR-ABL* and *Mir142*^*−/−*^*BCR-ABL* mice and T cells from *Mir142*^*−/−*^ and *Mir142*^*+/+*^ normal mice and co-cultured four different cell combinations for 72 h in the presence of activating anti-CD3/CD28 antibody-coated beads +/- PD-1 blocking Ab (Supplementary Fig. 16a). We observed increased PD-1 expression on *Mir142*^*−/−*^ T cells along with increased LSK viability in the co-cultures of *Mir142*^*−/−*^ LSK/*Mir142*^*−/−*^ T cells compared to those of *Mir142*^*−/−*^ LSK/*Mir142*^*+/+*^ T cells (Supplementary Fig. 16b–d). T cell PD-1 expression and LSK growth were rescued by PD-1 Ab (1 µg/ml, 96 h; Supplementary Fig. 16b–d). To confirm these results in vivo, we treated a cohort of *Mir142*^*−/−*^*BCR-ABL* mice with PD-1 blocking Ab (BioXcell, cat: BE0273) or IgG control (10 mg/kg, ip, 3x/week for 4 weeks) starting at day 2 post BCR-ABL induction (Supplementary Fig. 16e). PD-1 Ab-treated mice survived significantly longer (median: 139 vs 75 days, *P* = 0.02; Supplementary Fig. 16f) than IgG-treated controls.

Of note, engraftment of BM MNCs from CD45.2 *Mir142*^*−/−*^*BCR-ABL* mice into CD45.1 normal wt recipients (Fig. 5a) was associated with both reduced miR-142 levels (Fig. 5b) and increased PD-1 expression (Supplementary Fig. 16g) in the host T cells of the recipients compared with those of the recipients of *Mir142*^*+/+*^*BCR-ABL* or *Mir142*^*+/+*^ BM MNCs. These features were partly rescued by M-miR-142 treatment (30 mg/kg/day, iv, for 3 weeks; Supplementary Fig. 16h). Similar results were observed in co-cultures of mouse T cells with BM MNCs from *Mir142*^*−/−*^*BCR-ABL, Mir142*^*+/+*^*BCR-ABL* or normal wt mice (Supplementary Figs.12d, 16i), or in co-cultures of human T cells with BM MNCs from BC CML or CP CML patients or healthy donors (Supplementary Figs. 12e, 16j). The reduced miR-142 levels and increased PD-1 expression in T cells co-cultured with BC CML MNCs were rescued by anti-IL-6 Ab treatment (Supplementary Figs. 13i, j and 16i, j, right panel).

TGFBR1 and TGFBR2 are validated miR-142-3p and -5p targets, respectively^28^. TGFBR1 and TGFBR2 form a heterodimeric complex receptor for TGF-β that reportedly upregulates PD-1 on T cells through a signaling pathway converging on NFATc1^29,30^. To this end, RNA-seq analysis supported enhanced TGF-β signaling in T cells (Supplementary Fig. 17a) from *Mir142*^*−/−*^*BCR-ABL* mice compared with those from *Mir142*^*+/+*^*BCR-ABL* mice. Furthermore, we observed significantly increased expression levels of *Tgfbr1*, *Tgfbr2*, *Nfatc1* and *Pdcd1* in BM-T and spleen-T cells from *Mir142*^*−/−*^*BCR-ABL* mice compared with those from *Mir142*^*+/+*^*BCR-ABL* mice by Q-RT-PCR (Supplementary Fig. 17b, c). Western blot analysis confirmed increased protein levels of Tgfbr1, Tgfbr2, Nfatc1 and Pdcd1 in spleen T cells from *Mir142*^*−/−*^*BCR-ABL* mice compared with those from *Mir142*^*+/+*^*BCR-ABL* mice (Supplementary Fig. 17d). Tgfbr1, Tgfbr2, Nfatc1 and Pdcd1 were also increased in the host T cells (Supplementary Fig. 17e), along with the decreased miR-142 levels (Fig. 5b), from the recipients of *Mir142*^*−/−*^*BCR-ABL* BM MNCs compared with the recipients of *Mir142*^*+/+*^*BCR-ABL* and *Mir142*^*+/+*^ BM MNCs. The increased levels of Tgfbr1, Tgfbr2, Nfatc1 and Pdcd1 in the T cells from *Mir142*^*−/−*^*BCR-ABL* mice were rescued by treatment with M-miR-142 (2 μΜ, 72 h; Supplementary Fig. 17f, g). Thus, we concluded that PD-1 upregulation in T cells carrying miR-142 deficit was likely mediated by an increased TGF-β signaling via upregulation of the miR-142 targets, TGFBR1 and TGFBR2, thereby suggesting that miR-142 deficit impacts on T cell activation and differentiation at least partly through TGF-β-mediated mechanisms^31^.

### M-miR-142 restores T cell antileukemic activity

**Fig. 6:**
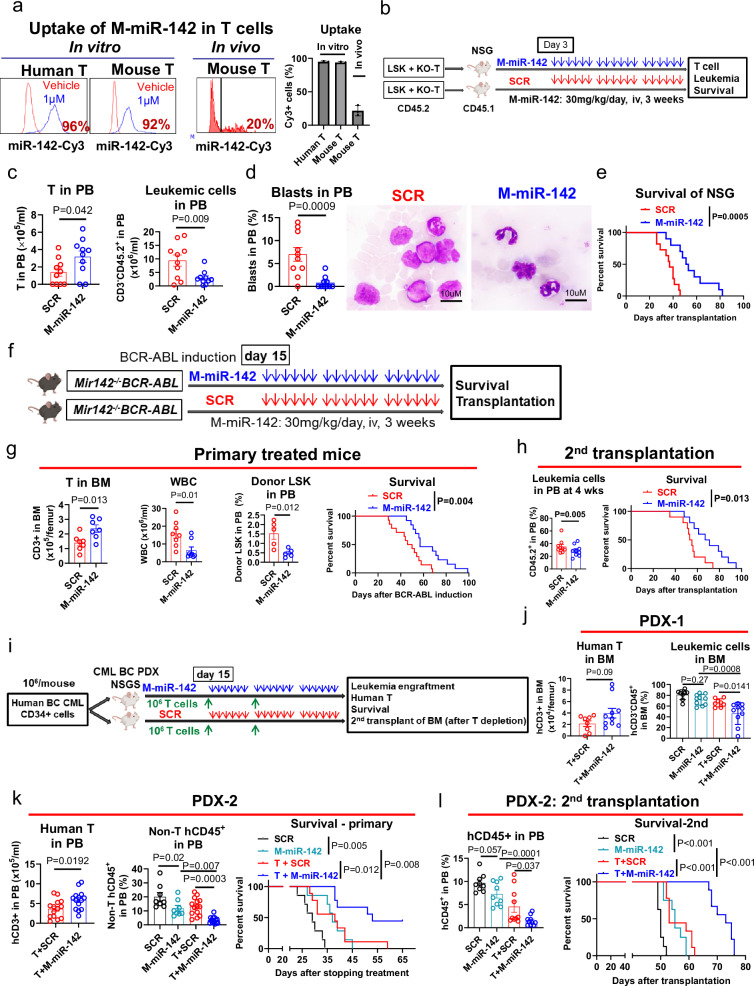
M-miR-142 restores T cell antileukemic activity. **a** Representative plots and combined results of uptake of Cy3-conjugated M-miR-142 by human and mouse T cells (in vitro: 1μΜ; in vivo: 30 mg/kg; *n* = 3). **b**–**e**. Experimental design and results. NSG mice were co-transplanted with *Mir142*^*−/−*^*BCR-ABL* LSK+*Mir142*^*−/−*^ T cells and then treated with SCR or M-miR-142 for 3 weeks (**b**), circulating T and leukemic cell counts (**c**), circulating blasts (%) and blood smear (**d**), and survival (**e**) were shown (*n* = 10 per group). **f**–**h**. Experimental design and results. *Mir142*^*−/−*^*BCR-ABL* mice were treated with SCR or M-miR-142 for 3 weeks (**f**), then BM T cells (*n* = 7 per group), WBC counts (*n* = 8), PB LSKs (*n* = 5), and survival (*n* = 14 for SCR group; *n* = 13 for M-miR-142 group) were shown (**g**). Leukemic cell engraftment (*n* = 9) and survival (*n* = 10) of the 2nd recipients were shown (**h**). **i**–**l** Experimental design and results. Two cohorts of BC CML PDX were given autologous human T cells and then treated with SCR or M-miR-142 (30 mg/kg/day, iv) for 3 weeks (**i**). Human T and leukemic cell engraftment in PDX-1 (**j**; *n* = 8 for SCR and T+ SCR groups; *n* = 12 for M-miR-142 and T+M-miR-142 groups) and PDX-2 (**k**; n = 8 for SCR and M-miR-142 groups; *n* = 14 for T+ SCR and T+M-miR-142 groups) and the survival of PDX-2 (**k**; *n* = 7 for SCR and M-miR-142 groups; *n* = 9 for T+ SCR and T+M-miR-142 groups) were shown. PB human leukemic cell engraftment and survival (**l**; *n* = 8 for SCR and M-miR-142 groups; *n* = 9 for T+ SCR and T+M-miR-142 groups) of the 2nd recipients receiving BM cells from the treated PDX-2 were shown. SCR scramble RNA, LSK Lin^−^Sca-1^+^c-Kit^+^, PDX patient-derived xenograft. For **c**, **d**, **g**, **h**, **j** and **k**, comparison between two groups was performed by two-tailed, unpaired *t*-test. For **j**–**l**, comparisons among multi-groups were performed by one-way ANOVA. For **e**, **g**, **h**, **k** and **l**, log-rank test was used to compare two or more survival curves. *P* values were corrected for multiple comparisons using Holm–Šídák method. Results shown represent mean ± SEM. For **b**, **f** and **i**, mouse images were created in BioRender. Chen, F. (2025) https://BioRender.com/e61c469. Source data are provided as a Source Data file.

To rescue miR-142 deficit of BC T cells, we produced synthetic miR-142-3p and -5p mimic hereafter called M-miR-142^32–34^. We confirmed effective drug uptake by human and mouse T cells using Cy3-conjugated M-miR-142 (Fig. 6a). We showed that treatment with M-miR-142 (2 μΜ) rescued the spontaneous apoptosis and poor cytokine production in T cells from *Mir142*^*−/−*^*BCR-ABL* mice (Supplementary Figs. 14i–k and 15a–d). Next, we performed in vivo experiments and delivered the drug (20 mg/kg M-miR-142-3p and 10 mg/kg M-miR-142-5p; hereafter collectively indicated as 30 mg/kg M-miR-142; see methods for details) by intravenous injection.

To test the in vivo activity of M-miR-142, we transplanted *Mir142*^*−/−*^*BCR-ABL* LSKs (CD45.2, 10,000/mouse) on day 1 and *Mir142*^*−/−*^ T cells (CD45.2, 10^6^/mouse) on day 2 into NSG mice (CD45.1). Starting on day 3, we treated these mice with M-miR-142 (30 mg/kg, iv) or SCR daily for 3 weeks (Fig. 6b). T cell (CD3^+^) and leukemic cell (CD45.2^+^ minus CD3^+^) engraftments were monitored by PB analysis. At the end of treatment, we observed a significant increase of circulating T cells (mean: 3.2 × 10^5^/ml vs 1.4 × 10^5^/ml, *P* = 0.042) and a decrease of circulating leukemic blasts (0.8% vs 7%, *P* = 0.0009) in the recipients treated with M-miR-142 compared with those treated with SCR (Fig. 6c, d). Importantly, we also observed an increased survival (median: 52 vs 37 days, *p* = 0.0005; Fig. 6e) of recipients treated with M-miR-142 vs those treated with SCR. To confirm these results also in mice with an already established BC phenotype, we treated a cohort of *Mir142*^*−/−*^*BCR-ABL* mice with M-miR-142 or SCR starting on day 15 after BCR-ABL induction (Fig. 6f). After 3 weeks of treatment, we observed increased BM T cell number (mean: 2.36 × 10^5^ vs 1.39 × 10^5^ per femur, *P* = 0.013), reduced circulating blasts (LSK: 0.48% vs 1.52%, *P* = 0.012), and significantly prolonged survival (median: 57 vs 48 days, *P* = 0.004) in M-miR-142-treated mice compared with SCR-treated controls (Fig. 6g). Secondary (2nd) recipients of BM cells from M-miR-142-treated donors also showed significantly longer survival than recipients of BM cells from SCR-treated donors (median: 69 vs 54 days, *P* = 0.013; Fig. 6h), suggesting that M-miR-142 may decrease LSC burden through restoring T cell activity.

To test the relevance of these findings to human disease, we first generated two BC CML PDX models with high human leukemic cell burden (both: >50%) by transplanting CD34^+^ cells from a few BC CML patients into NSG-SGM3 (NSGS) mice. Two weeks after transplantation, >5% circulating human CD45^+^ cells were detected and each mouse received autologous human T cells (T cells from the same patient, expanded by anti-CD3/CD28 Ab-coated beads for 2 weeks, 10^6 ^T cells on day 14 and day 21 respectively), and SCR (as control) or M-miR-142 (30 mg/kg/day, iv) for 3 weeks (Fig. 6i). Of note, since untreated PDX model 1 had a prolonged elapsed time-to-death (>16 weeks), only changes in leukemic burden, rather than median survival, were evaluated as the endpoint for treatment activity. In contrast, since untreated PDX model 2 had a relatively short median survival (≤60 days), changes in both leukemia burden and survival were measured as the endpoint for treatment activity. PDX-1 mice that received T cells and M-miR-142 had a trend for increased human T cells (BM: 3.96 × 10^4^ vs 2.125 × 10^4^ per femur, *P* = 0.09) and a significant reduction in human leukemic burden (BM: 46.22% vs 66.01%, *P* = 0.0141) compared with the mice receiving T cells and SCR (Fig. 6j). PDX-2 mice that received T cells and M-miR-142 had a significant increase of human T cells (PB: 5.9 × 10^5^/ml vs 3.67 × 10^5^/ml, *P* = 0.0192), decrease of leukemic burden (PB: 3.35% vs 13.12%, *P* = 0.0003), and prolonged survival (median: 53 vs 38 days, *P* = 0.012) compared with controls receiving T cells and SCR (Fig. 6k).

Of note, after prolonged follow-up, while the T+ SCR-treated PDX-2 mice died of leukemia, the T+M-miR-142-treated PDX-2 mice showed very low leukemic burden (<5% in PB and BM and spleen) and high T cell percentage (>50%), with diarrhea, weight loss, fur ruffling, hunching, and lethargy, suggesting that these mice eventually developed graft-vs-host disease (GVHD). To evaluate the impact of treatment on LSC burden, after completion of treatment of another cohort of PDX-2 mice, we transplanted T cell-depleted BM MNCs (10^6^) from T+M-miR-142- or T+ SCR-treated primary mice into 2nd NSGS recipients. Significantly lower human cell engraftment rates (1.5% vs 4.6% at 4 weeks post-transplantation; *P* = 0.037; Fig. 6l) and increased survival (median: 73 vs 53 days, *P* < 0.001; Fig. 6l) were observed in the recipients of BM from T+M-miR-142-treated donors compared to the recipients of BM from T+ SCR-treated donors.

### Enhanced antileukemic activity of M-miR-142 in combination with TKI and PD-1 antibody

**Fig. 7:**
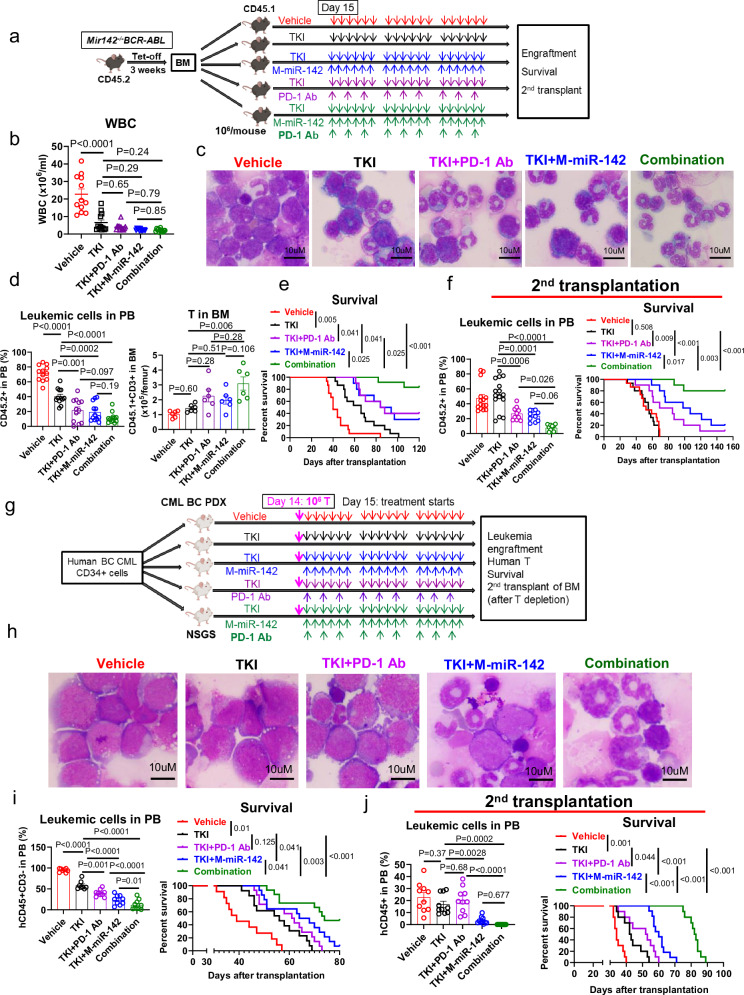
M-miR-142 in combination with TKI and PD-1 Ab showed enhanced antileukemic activity in BC CML models. **a**–**f** Experimental design and results. A cohort of BC CML mice were treated with NIL (30 mg/kg/day, oral gavage), NIL+M-miR-142(30 mg/kg/day, iv), NIL+PD-1 Ab (10 mg/kg, 3x/week), NIL+M-miR-142+PD-1 Ab, or vehicle for 3 weeks (**a**; *n* = 15 per group). WBC counts (**b**), blood smear (**c**), leukemic cell engraftment and host T cell percentages in PB (**d**), and survival of the treated mice (**e**) are shown. BM cells from the treated mice were transplanted into 2nd recipients (*n* = 15 for vehicle and NIL groups and n = 10 for the remaining three groups). Leukemic cell engraftment in PB and survival of the 2nd recipients (**f**) are shown. **g**–**j** Experimental design and results. A cohort of BC CML PDX mice were given autologous human T cells (10^6^/mouse on day 14) and 3 weeks’ treatment with vehicle, NIL (30 mg/kg/day, oral gavage), NIL+M-miR-142(30 mg/kg/day, iv), NIL+PD-1 Ab (10 mg/kg, 3x/week), or NIL+M-miR-142+PD-1 Ab (**g**). Blood smear (**h**), human (h) leukemic cell engraftment in PB (**i**, left; % of hCD45^+^ minus % of hCD3^+^; *n* = 8 for vehicle and NIL groups; *n* = 9 for NIL+M-miR-142 and NIL+PD-1 Ab groups; *n* = 10 for NIL+M-miR-142+PD-1 Ab group) and survival (**i**, right; *n* = 11 for vehicle group; *n* = 13 for NIL group; *n* = 14 for NIL+M-miR-142 and NIL+PD-1 Ab groups; *n* = 15 for NIL + M-miR-142 + PD-1 Ab group) were monitored. BM cells from the treated mice were transplanted into 2nd NSGS recipient mice (*n* = 10 per group). Human cell engraftment in PB and survival of the 2nd recipients are shown (**j**). TKI tyrosine kinase inhibitor, Ab antibody, BC blast crisis, CML chronic myeloid leukemia, NIL nilotinib, WBC white blood cell, PB peripheral blood, 2nd: secondary; PDX: patient-derived xenograft. For **b**, **d**, **f**, **i** and **j**, comparisons among multi-groups were performed by one-way ANOVA. For **e**, **f**, **i** and **j**, log-rank test was used to compare survival curves among multi-groups. *P* values were corrected for multiple comparisons using Holm–Šídák method. Results shown represent mean ± SEM. For **a** and **g**, mouse images were created in BioRender. Chen, F. (2025) https://BioRender.com/e61c469. Source data are provided as a Source Data file.

Since TKIs represent a first line targeting approach for CML, next, we tested if targeting miR-142 deficit and/or the consequential PD-1 upregulation enhanced TKI activity in BC models. To this end, we generated a cohort of BC CML mice by transplanting leukemic blasts from diseased CD45.2 *Mir142*^*−/−*^*BCR-ABL* mice into congenic normal wt CD45.1 recipients. On day 15 after transplantation, we treated the recipient mice with TKI (nilotinib, 30 mg/kg/day, oral gavage), TKI+M-miR-142 (30 mg/kg/day, iv), TKI+PD-1 blocking Ab (10 mg/kg, ip, 3x/week), TKI+M-miR-142+PD-1 Ab (“triplet”), or vehicle for 3 weeks (Fig. 7a). We observed significantly reduced donor-cell derived leukemic burden (mean: 40.17% vs 71.64%, *P* < 0.0001) and prolonged survival (median: 66 vs 40 days, Log-rank test, *P* = 0.005) in the TKI-treated group compared with the vehicle-treated group (Fig. 7b–e). TKI+M-miR-142 treatment decreased BC CML leukemic burden in PB (19.23 vs 40.17%, *P* = 0.0002) and prolonged survival (median: 89 vs 66 days, *P* = 0.041) compared to TKI alone treatment (Fig. 7b–e). Treatment with TKI+PD-1 Ab also decreased circulating leukemic burden (mean: 21.64% vs 40.17%, *P* = 0.001) and prolonged survival (median: 86 vs 66 days, *P* = 0.041) compared to TKI treatment (Fig. 7c–e). Of note, mice treated with the “triplet” TKI+M-miR-142+PD-1 Ab had fewer leukemic blasts and longer survival (median: unreached after monitoring for 120 days) compared with TKI+M-miR-142 (89 days, *P* = 0.025), TKI+PD-1 Ab (86 days, *P* = 0.025), TKI (66 days, *P* < 0.001), or vehicle (40 days, *P* < 0.001) -treated mice (Fig. 7c–e). BM cells from the treated mice were also transplanted into 2nd recipient mice to evaluate post-treatment LSC burden. In 2nd transplant experiments, recipients of BM cells from the “triplet”-treated mice also had lower leukemic burden (mean: 7.7%) than TKI+M-miR-142 (23.75%, *P* = 0.06), TKI+PD-1 Ab (26.74%, *P* = 0.026), TKI (52.98%, *P* < 0.0001), or vehicle (47.52%, *P* < 0.0001) -treated mice at 4 weeks post transplantation and longer survival (median: unreached after monitoring for 150 days) than TKI+M-miR-142 (102.5 days, *P* = 0.017), TKI+PD-1 Ab (77 days, *P* = 0.003), TKI (55 days, *P* < 0.001), or vehicle (52 days, *P* < 0.001) -treated mice (Fig. 7f).

Next, we generated a cohort of BC CML PDX-2 mice as described above. Two weeks after transplantation and upon detecting >5% circulating human CD45^+^ cells, each mouse received autologous human T cells (10^6^ T cells on day 14), and three-week treatment with vehicle or TKI alone, doublet (TKI+M-miR-142 or TKI+PD-1 Ab) or triplet (TKI+M-miR-142+PD-1 Ab; see methods for details; Fig. 7g). We observed lower percentage of leukemic blasts and human BC CML cells (mean: 58.4% vs 94.56%, *P* < 0.0001) and longer survival (median: 56 vs 39 days, *P* = 0.01) in the TKI-treated group than in the vehicle-treated group (Fig. 7h, i). TKI+M-miR-142 treatment significantly decreased human BC CML leukemic burden (mean: 23.42% vs 58.4%, *P* < 0.0001) and prolonged survival (median: 66 vs 56 days, *P* = 0.041) compared to TKI treatment (Fig. 7h, i). Treatment with TKI+PD-1 Ab also reduced human BC CML leukemic burden (mean: 40.01% vs 58.4%; *P* = 0.001) but did not change survival significantly (median: 61.5 vs 56 days, *P* = 0.125) compared to TKI alone (Fig. 7i). Mice treated with “triplet” had lower leukemic burden (mean: 11.55%) after 3 weeks’ treatment and longer survival (median: 74 days) than TKI+M-miR-142 (mean: 23.42%, *P* = 0.01; median: 66 days, *P* = 0.041), TKI + PD-1 Ab (40.01%, *P* < 0.0001; 61.5 days, *P* = 0.003), TKI (58.4%, *P* < 0.0001; 56 days, *P* < 0.001), or vehicle (94.56%, *P* < 0.0001; 39 days, *P* < 0.001) -treated mice (Fig. 7h, i). In 2nd transplant experiments, recipients of BM MNCs from triplet-treated mice also had lower leukemic burden than the other groups at 4 weeks post transplantation and survived longer (median: 82.5 days) than the recipients of BM MNCs from TKI+M-miR-142 (60 days, *P* < 0.001), TKI+PD-1 Ab (52.5 days, *P* < 0.001), TKI (42.5 days, *P* < 0.001), or vehicle (34 days, *P* < 0.001) -treated mice (Fig. 7j).

## Discussion

While autonomous mechanisms of transformation of CP-LSCs into BC-LSCs have been extensively studied^35^, the impact of the leukemic microenvironment, including the immune system in the BC transformed niche, remains to be fully elucidated. We have recently shown that miR-142 deficit acquired by CP-LSCs enhances mitochondrial fusion and OxPhos and transforms these cells into BC-LSCs^16^. Previous reports have suggested that lower miR-142 levels associated with accelerated phase and reduced response to TKI treatment in CML patients^36–38^. Using BC GEMM and PDX models, herein we show that during BC evolution, an acquired miR-142 deficit causes a loss of T cells, thereby allowing BC immune escape. Using a combination of scRNA-seq and immunophenotypic analyses and in vivo differentiation studies, we demonstrated that miR-142 deficit redirected LMPPs preferentially toward the myeloid lineage differentiation in *Mir142*^*−/−*^*BCR-ABL* mice compared with those from the control mouse strains (i.e., *Mir142*^*+/+*^, *Mir142*^*−/−*^ or *Mir142*^*+/+*^*BCR-ABL*). Furthermore, we showed that miR-142 deficit also impaired LMPPs’ downstream thymic T differentiation. To this end, the thymus of the *Mir142*^*−/−*^*BCR-ABL* mouse was significantly smaller than that of the *Mir142*^*+/+*^*BCR-ABL* mouse, and the thymic T cell precursors were found arrested at the DN stage. In the *Mir142*^*−/−*^*BCR-ABL* mouse, the fewer mature T cells that eventually emerged were depleted in Tnaïve and Treg and enriched in CD8^+^ Tcm and Teff. These results are consistent with a recent report showing that Treg-miR-142 KO in miR-142^f/f^Foxp3Cre mice causes reduction of Treg number and activity^39^. Thus, Treg reduction, along with increase in cytokines released by leukemic blasts, could potentially explain the Teff enrichment observed in the *Mir142*^*−/−*^*BCR-ABL* mouse. However, the enrichment in CD8^+^ Tcm and Teff cells in the *Mir142*^*−/−*^*BCR-ABL* mouse did not translate into antileukemic activity. To this end, Teff cells in the *Mir142*^*−/−*^*BCR-ABL* mouse showed higher immune checkpoint levels, less cytokine production, loss of the activating metabolic switch (i.e., concurrent increase in glycolysis and OxPhos)^24^ and an increased rate of spontaneous apoptosis compared with those from the control *Mir142*^*+/+*^*BCR-ABL* mouse. Furthermore, we confirmed decrease in antileukemic activity both in vitro using co-cultures of LSK blasts with *Mir142*^*−/−*^ T cells, and in vivo using combinatorial transplants of *Mir142*^*−/−*^*BCR-ABL* LSK and *Mir142*^*−/−*^ *or Mir142*^*+/−*^ or *Mir142*^*+/+*^ T cells into congenic recipient mice. In fact, leukemic mice hosting *Mir142*^*−/−*^ or *Mir142*^*+/−*^ T cells invariably had a shorter survival compared with those hosting *Mir142*^*+/+*^ T cells. Based on these results, we then postulated that T cell immune escape mechanisms could be mediated by T lymphopenia due to a cytokine-mediated miR-142 deficit acquired during BC evolution. Of note, while we demonstrated in vitro decrease of cytokine production and cytotoxic activity, whether this functional impact also contributed to leukemia immune escape remains to be fully elucidated. Reduced miR-142 levels and T lymphopenia were also observed in the BC vs CP CML samples, even though the difference in miR-142 levels between BC vs CP CML could also be accounted for by the difference in T cell subtypes.

Of note, while GEMMs are valuable tools to study the functional consequence of miR-142 deficit and how it impacts leukemogenesis, they are not useful to explain how miR-142 deficit is acquired by T cells during human BC transformation. To this end, we showed that IL-6 was increased both in BC mouse models and primary patient blasts. IL-6 was previously reported to be higher in BC CML patients than in CP CML patients^25,40^ and to be both a target and a regulator of miR-142^26^. Our data supports that IL-6 secreted by growing myeloid blasts contributes to miR-142 downregulation both in LMPP and T cells, thereby reducing T cell production, persistence and in turn their antileukemic activity. Of note, IL-6 has also been shown to be involved in inducing PD-1 expression through mechanisms that remain to be fully elucidated^41^. Our study provided evidence that IL-6 can induce PD-1 upregulation in T cells via miR-142 downregulation. To this end, TGFBR1 and TGFBR2 are reported targets of miR-142-3p and -5p respectively^28^ and our RNA-seq data showed upregulation of TGF-β signaling in the T cells from the *Mir142*^*−/−*^*BCR-ABL* mice. Accordingly, we showed IL-6-induced miR-142 deficit resulted in TGFBR1/TGFBR1 upregulation, enhanced TGF-β/Nfatc1 signaling and ultimately PD-1 upregulation, which possibly impact on T cell activity. Of note, CD8^+^ T cell exhaustion has also been reportedly associated with high levels of PD-1 and decrease in cytotoxicity, proliferation, and cytokine production in CML^42–46^. Taken together, our data showed that enhanced TGF-β signaling driven by miR-142 deficit could associate with PD-1 upregulation and possibly with a negative impact on T cell activity, as previously reported^28,47,48^.

We believe that our observations have a twofold clinical relevance. Firstly, they add novel insights to the mechanisms of BC transformation, which also included acquisition of overexpressed or mutated driver genes that could impact both on leukemic growth and immune evasion^35^. Secondly, as we showed that BC CML immune escape was rescued by M-miR-142^16^, a synthetic miR-142 mimic, they may offer conceptually new treatment strategies. In fact, BC CML GEMMs and PDXs carrying miR-142^−/− or low^ T cells have a more rapid disease growth and shorter survival than controls with miR-142^+/+^ T cells. Treatment of these mice with M-miR-142 rescued the BC phenotype and prolonged survival compared with those treated with SCR control, by restoring T cell activity and reducing mitochondrial oxidative metabolism in LSCs^16^. The recent progress made in the design and formulation of RNA-based therapeutics supports the growing enthusiasm for this class of compounds and the increasing hope that they can be used to aim at otherwise non druggable targets. One of the advantages of leukemia-targeted miRNA-based therapeutics is the ability to concurrently target distinct compartments of the leukemic niche, thereby affecting both intrinsic and extrinsic mechanisms of LSC homeostasis. To this end, miR-142 deficit drives BC transformation by acting both on LSCs^16^ and T cells. Of note, the activity of M-miR-142 was enhanced by the PD-1 blocking Ab, and the triple combination of M-miR-142, PD-1 Ab and TKI induced the longest survival compared with any doublet combination or single agent, thereby opening a window for novel therapeutic approaches for BC patents. Of note, it is likely that our observation can be extended to other types of myeloproliferative neoplasms transforming into secondary AML.

Of note, miR-142 reportedly regulates differentiation and activation of immune cells other than T cells [i.e., B, dendritic cells (DC), NK cells and myeloid cells]^4,5,9,49^. While our study showed that miR-142 deficit affects the output of T cells from LMPP as well as the persistence and activity of mature T cells, changes in other immune cell subpopulations may also occur and contribute to the immune escape and disease growth.

In conclusion, herein we unveil novel immune escape mechanisms of BC transformation mediated by an acquired miR-142 deficit that causes loss of T cells. The miR-142 deficit is promptly rescued with a synthetic miR-142 mimic, M-miR-142, that alone or in combination with PD-1 Ab and/or TKI extends survival of BC murine and PDX models. IND-enabling studies for M-miR-142 are also underway for a rapid translation of our observations from the bench to the bedside.

## Methods

Our research complies with all relevant ethical regulations. Animal experiments were carried out following the federal guidelines and protocols were approved by the Institutional Animal Care and Use Committee at City of Hope.

### Human samples

Normal PB and BM samples were obtained from healthy donors at the City of Hope National Medical Center (COHNMC). CP and BC CML samples were obtained from patients who had not received TKI treatment at the COHNMC. All CML samples used in this study are P210 BCR–ABL positive, as confirmed by FISH analysis and qPCR. MNCs were isolated using Ficoll™ separation. When necessary, CD34+ cells were then isolated using a positive magnetic bead selection protocol (Miltenyi Biotech, Germany). All CML patients and healthy donors signed an informed consent form. Sample acquisition was approved by the Institutional Review Board (IRB # 06229 and IRB# 18067 protocols) at the COHNMC, in accordance with an assurance filed with and approved by the Department of Health and Human Services and met all requirements of the Declaration of Helsinki.

### Animal studies

All mouse models used in this study are in CD45.2 C57Bl/6j background and 6–10 weeks old male and female mice were used. SCLtTA/BCR-ABL transgenic mice (hereafter called BCR-ABL)^17,50^ were maintained on tetracycline (tet)-containing water at 0.5 g/l. Withdrawal of tet results in expression of BCR-ABL and generation of a CP CML-like disease in these mice^17,50^. MiR-142 KO (i.e., *Mir142*^*−/−*^) and *Mir142*^*f/f*^ mice were generated by our collaborator Dr. Mark Boldin^4^. *Mir142*^−/−^*BCR-ABL* mice were generated by crossing *Mir142*^*−/−*^ with *BCR-ABL* mice. *Mir142*^*f/f*^*Lck-cre*^+^(miR-142 KO in T cells, also called *Mir142*^TΔ/Δ^) mice were generated by crossing *Mir142*^*f/f*^ with *Lck-cre*^+^(Jax lab, 3802) mice. *Mir142*^TΔ/Δ^*BCR-ABL* mice were generated by crossing *Mir142*^*f/f*^*Lck-cre*^+^ mice with *BCR-ABL* mice. The genotyping of the above mice was performed by Transnetyx. All *Mir142*^+/+^*BCR-ABL* and *Mir142*^−/−^*BCR-ABL* mice used in this study were 6-10 weeks old and BCR-ABL expression was induced by tet-off for 3 weeks, unless indicated differently. To evaluate the contribution of miR-142 deficit in T cells to BC disease growth, we co-transplanted *Mir142*^−/−^*BCR-ABL* LSKs with *Mir142*^*+/+*^ T cells or *Mir142*^*+/−*^ T cells or *Mir142*^*−/−*^ T cells into CD45.1 normal wt recipients [irradiated with 6Gy (X-ray) to deplete T cells] or NSG recipients (lacking T cells). We also transplanted *Mir142*^−/−^*BCR-ABL* LSKs or *Mir142*^+/+^*BCR-ABL* LSKs into *Mir142*^*−/−*^ (homozygous KO), *Mir142*^*+/−*^ (heterozygous KO), or *Mir142*^*+/+*^ (wt) recipients, or into *Mir142*^*−/−*^ (global KO), *Mir142*^TΔ/Δ^ (T-KO), or wt recipients. To evaluate the impact of expanded leukemic blasts on host T cells, we transplanted BM MNCs from CD45.2 normal wt, *Mir142*^+/+^*BCR-ABL* (CP CML), or *Mir142*^−/−^*BCR-ABL* (BC CML) mice into congenic CD45.1 normal wt recipient mice and measured miR-142 levels and activity of CD45.1^+^CD3^+^ host T cells in the recipients at 2 and 4 weeks post transplantation. Six- to ten-week-old CD45.1 C57Bl/6j (from Charles River) male or female mice were irradiated at 6Gy (X-ray) within 24 hrs before transplantation and used as recipients to allow tracking of CD45.2 C57Bl/6j donor cells. The number of mice for each study group was chosen based on the expected endpoint variation (i.e., engraftment rate and latency period of leukemia) and on the availability of mice from different strains. To achieve a meaningful comparison, we matched *Mir142*^−/−^*BCR-ABL* and *Mir142*^*+/+*^*BCR-ABL* mice not only for age and gender but also for SCL and BCR-ABL level (by Q-RT-PCR). Investigators were blinded to mouse genotype while performing treatment or monitoring for engraftment or survival. All the experimental mice were housed in 68-79F temperature and 30-70% humidity, in a 12:12-h light:dark cycle. Mice are group-housed in individually ventilated cages (Optimice, Animal Care Systems, Centennial, CO). Mice are allowed free access to rodent chow (no. 5053, LabDiet, St Louis, MO), and reverse-osmosis–purified water. Mouse care and experimental procedures were performed in accordance with federal guidelines and protocols and were approved by the Institutional Animal Care and Use Committee at City of Hope.

### Flow cytometry analyses

Human T cells and CD34^+^ cells were selected using the CD3 microbeads and indirect CD34 microbead kit (Miltenyi Biotec, San Diego, CA) respectively. For flow cytometry analysis, cells were washed and suspended in phosphate-buffered saline (PBS; Gibco) supplemented with 1% BSA and stained with corresponding Abs (Supplementary Table 2). Mouse cells were obtained from PB, BM (both tibias and femurs), or spleen. To measure intracellular cytokine production, mouse and human T cells were treated with stimulation cocktail (PMA plus ionomycin, eBioscience) and protein transport inhibitor (Brefeldin A and Monensin, eBioscience) for 5 h according to the manufactures’ protocol, then T cells were fixed and permeabilized using Foxp3/Transcription Factor Staining Buffer Set (eBioscience) and stained with Abs against intracellular cytokines. The mouse and human Abs used in this study were described in Supplementary Table 2. Stem and progenitor subpopulations were identified as LSK (Lin^−^Sca-1^hi^c-Kit^hi^). Lymphoid-primed multipotent progenitors (LMPPs) were identified as Flt3^+^CD150^−^ LSK^17,51^. To sort LSK or LMPP cells, c-kit^+^ cells were selected with anti-mouse CD117 microbeads (Miltenyi Biotec, San Diego, CA) or Lin^−^ cells were selected with Lineage depletion microbeads (Miltenyi Biotec, San Diego, CA) and then stained with mouse Abs before sorting. To determine engraftment rate of human CML cells in NSGS mice, PB, BM and spleen cells were stained with anti-human CD45, CD33 and CD34 Abs (Supplementary Table 2). All analyses were performed on a Fortessa x20 flow cytometer (BD Biosciences) and sorting was performed on Aria Fusion instrument (BD Biosciences) and data were analyzed by BD FACSDiva (v9.0) or FlowJo (v10.8.1) software.

### LMPP in vitro and in vivo differentiation assay

LMPP (Flt3^+^CD150^−^ LSK) cells were sorted from the BM of *Mir142*^*+/+*^*BCR-ABL* or *Mir142*^*−/−*^*BCR-ABL* mice (BCR-ABL were induced by tet-off for 3 weeks). To support the differentiation of LMPP cells into T lymphocytes, OP9-DL1 stromal cells (RIKEN) were cultured in MEMα medium with 20%FBS in 24 well plates until 50% confluent, then 5 × 10^4^ LMPPs in 1 mL of MEMα (without nucleotide) medium with 20% FBS, 5 ng/mL of Flt3L and 1 ng/ml of IL-7 were seeded on the feeder OP9-DL1 cells. LMPPs were passaged every 3 or 4 days and transferred to a new plate of OP9-DL1 cells with 50% confluent. On day 6, 10, 15, and 19, cell numbers were counted; cells were aliquoted for RNA isolation and flow cytometry analysis of myeloid, B and T lineage differentiation by staining with CD11b, CD19, CD25, CD44, CD3, CD4 and CD8 Abs (Supplementary Table 2).

To compare LMPP thymic homing and differentiation, we next transplanted BM LMPPs from CD45.2 *Mir142*^*−/−*^*BCR-ABL* and *Mir142*^*+/+*^*BCR-ABL* mice into normal wt CD45.1 recipients. We also transplanted BM LMPPs from CD45.2 *Mir142*^*−/−*^ or *Mir142*^*+/+*^ mice into CD45.1 normal wt recipients. Thymic homing of LMPPs at 24 h were measured. After transplantation, donor LMPP-derived cells, including CD4 and CD8 DN, DP, and SP cells, in thymus on day 14 and mature T cells, including αβ T and γδ T cells in PB, BM and spleen on day 28 after transplantation were analyzed by flow cytometry.

### Cell Culture

Murine BM LSKs were cultured in Stemspan™ serum-free medium II (SFEM II, StemCell Technologies) supplemented with 10 ng/ml SCF and 10 ng/ml TPO. Human and mouse T cells were cultured in advanced RPMI1640 medium with 10% FBS supplemented respectively with human or mouse 100 μ/ml IL-2 and 2.5 ng/ml IL-7. The co-culture of T cells and BM MNCs was conducted in a 24-transwell plate with lower and upper chambers separated by a membrane with 0.4 μm-diameter pores (Corning Costar). BM MNCs (2 × 10^6^ per well) were seeded into the lower chamber and T cells (2 × 10^5^ per well) were added into the upper compartment. T cells were harvested at 72 h for RNA extraction and flow cytometry analysis. All the cultured cells were maintained at 37 °C with 5% CO_2_ and high humidity.

### Apoptosis, cell cycling and cell growth

Human T cells from CP CML or BC CML patients and murine T cells from *Mir142*^*+/+*^*BCR-ABL* (CP CML) or *Mir142*^−/−^*BCR-ABL* (BC CML) mice were analyzed for apoptosis, cell cycling and cell growth. T cells were also treated with scrRNA or M-miR-142 (2 µM) or miR-142 inhibitor (2 µM), for 72 h and analyzed for apoptosis, cell cycling and cell growth. Apoptosis was measured by labeling cells with annexin V (Supplementary Table 2) and 4, 6‐diamidino‐2‐phenylindole (DAPI, BD-PharMingen, San Diego, CA) and analyzed by flow cytometry. Cell cycling was measured by Ki-67/DAPI staining (Supplementary Table 2). T cells’ ex vivo proliferation rate was also analyzed by CellTrace™ Violet Cell Proliferation Kit (Thermal Fisher). T cells were pre-stained with 5 μM Celltrace™ violet dye and then cultured with CD3/CD28 Dynabeads™ for 3 days, followed by flow cytometry analysis of T cell proliferation. Cell growth was detected using Cell-Titer Glo® Luminescent Cell Viability Assay (Promega) following the manufacturer’s protocol.

### Gene expression by Q-RT-PCR

To measure the miRNA and mRNA expression, total RNA was extracted using the miRNeasy Mini Kit (Qiagen, Valencia, CA). For miRNA expression, reverse transcription using MultiScribe™ Reverse Transcriptase and Q-PCR analysis using Taqman assays (Applied Biosystems; Supplementary Table 3) were performed according to the manufacturer’s protocol. RNU44 and snoRNA234 were used as internal controls for human and mouse miRNA respectively. For mRNA expression, first-strand cDNA was synthesized using the SuperScript III First-Strand Kit and then Q-PCR was performed using TaqMan Gene Expression Assays (Applied Biosystems; Supplementary Table 3). *BCR-ABL* expression in human and mouse samples were measured with primer and probe sequences for *BCR-ABL* (B3A2 or B2A2), as previously described^52^. B2M was used as internal controls, and the results are presented as log2-transformed ratio according to the 2^–ΔCt^ method (ΔCt=Ct of target –Ct of reference).

### Single cell RNA sequencing

Murine BM LMPPs from *Mir142*^*+/+*^*, Mir142*^*−/−*^*, Mir142*^*+/+*^*BCR-ABL* (CP CML), and *Mir142*^*−/−*^*BCR-ABL* (BC CML) mice (BCR-ABL was induced by tet-off for 3 weeks) and human LMPPs from CP CML or BC CML patients were sorted for scRNA-seq. PB MNCs and BM and spleen T cells (CD3^+^) isolated from *Mir142*^*+/+*^*BCR-ABL* and *Mir142*^*−/−*^*BCR-ABL* mice (BCR-ABL was induced by tet-off for 3 weeks) were also subjected to scRNA-seq. Flow cytometry sorted single cells were captured on a Chromium Controller (10xGenomics) using a single cell 3’ Reagent V3.1 Chemistry Dual Index kit (10X Genomics, PN-1000121) targeting ~5000–7000 cells/sample. Single-cell RNA-seq libraries were constructed by following the manufacturer’s instruction. The cDNA and library quality were analyzed on a High Sensitivity DNA Chip (Agilent Technologies, #5067-4626). Qubit High Sensitivity DNA assay Kit (ThermoFisher Scientific) was used for sequencing library quantification. The sequencing was performed on Illumina NovaSeq 6000 platform (Illumina) at The Translational Genomics Research Institute (TGen) with the sequencing depth of 50K–75K reads/cell. Real-time analysis (RTA) v3.4.4 software was used to process the image analysis.

Raw sequencing data were aligned back to the mouse genome (mm10) using cellranger count command to produce expression data at a single-cell resolution according to 10x Genomics (https://support.10xgenomics.com/single-cell-gene-expression/software/pipelines/latest/using/count).

The Seurat R package^53^ was employed to facilitate various critical data processing and analysis tasks, including gene and cell filtration, data normalization, principal component analysis (PCA), identification of variable genes, clustering analysis, and Uniform Manifold Approximation and Projection (UMAP) dimension reduction. To provide a succinct overview of the methodology, individual matrices containing gene-by-cell expression data were imported to create distinct Seurat objects for each sample. Subsequently, cells with fewer than 200 detectable genes and a mitochondrial gene content exceeding 15% were systematically excluded. Following this, the data from different samples were harmoniously integrated using scTransform package for subsequent analytical procedures. Dimensionality reduction was carried out through PCA and the first few principal components were selected based on an elbow plot and retained for clustering. The resulting clusters were visually represented using UMAP embedding, and a comprehensive examination of gene expression patterns was conducted through the utilization of various visualization techniques, including VlnPlot, FeaturePlot, and DotPlot. The T cell clusters were annotated based on expression patterns of known markers shown in Supplementary Table 1. Differentially expressed (DE) genes in each cluster or using whole cells between two samples were discovered with function FindAllMarkers. Gene ontology (GO) and Kyoto encyclopedia of genes and genomes pathway analysis were performed with DE genes using GSEA function implemented in clusterProfiler package^54^, then being plotted with ggplot2 (H. Wickham. ggplot2: Elegant Graphics for Data Analysis. Springer-Verlag New York, 2016.).

### Bulk RNA sequencing

T cells (2 × 10^5^/sample) from the spleen of *Mir142*^*+/+*^*BCR-ABL* (CP CML, *n* = 8 mice) and *Mir142*^*−/−*^*BCR-ABL* (BC CML, *n* = 6 mice) (BCR-ABL were induced by tet-off for 3 weeks) mice were sorted, and total RNA was extracted using the miRNeasy Micro Kit (Qiagen, Valencia, CA). RNA sequencing libraries were prepared with Kapa mRNA HyperPrep kit (Kapa Biosystems) according to the manufacturer’s protocol. RNA-seq libraries were sequencing on Illumina NovaSeq6000 with the sequencing length of 2 × 101 bp. RNA-Seq reads were trimmed to remove sequencing adapters using Trimmomatic^55^ and polyA tails using FASTP^56^. The processed reads were mapped to the mouse genome mm10 using STAR (v. 2.6.0.a)^57^, and gene expression levels were summarized by HTSeq-count v.0.11.1^58^. Gene expression raw counts were normalized using TMM normalization method and differential expression analysis was conducted using log likelihood ratio test implemented in “edgeR”^59–62^. DEGs between BC vs CP T cells were considered significant with an FDR-adjusted *p*-value less than 0.05. Genes ranked according to their log2 fold change and *p*-values were then subjected to pre-ranked GSEA analysis using hallmark pathways. Heatmaps were generated using “pheatmap” package.

### Untargeted metabolomics

CD3^+^ T cells were sorted by flow cytometry from the spleen of *Mir142*^*−/−*^*BCR-ABL* and *Mir142*^*+/+*^*BCR-ABL* mice (BCR-ABL expression was induced by tet-off for 3 weeks, *n* = 9 mice per group). After sorting, T cells from 5 mice per group were snap-frozen in liquid nitrogen as resting T cells. T cells from 4 mice per group were cultured in advanced RPMI1640 with 10% FBS and 50 μ/ml IL-2 in the presence of Dynabeads™ Mouse T-Activator CD3/CD28 (11452D, ThermoFisher) for 24 h and then snap-frozen as activated T cells. Metabolite extraction was performed on resting (6 × 10^6^/sample, *n* = 5 sample per group) and activated (3 × 10^6^/sample, *n* = 4 sample per group) T cells from both *Mir142*^*−/−*^*BCR-ABL* and *Mir142*^*+/+*^*BCR-ABL* mice and subjected to untargeted metabolomics as described previously^16^. A matrix pooled QC was used for determining instrument variability, performing batch correction and normalization^16,63^. System suitability was performed before and after batch using inhouse standard metabolite extract^16,63^.The raw data were processed using Compound discoverer 3.2 where minimum peak intensities of 70,000 and 60,000 for the respective positive and negative modes were used as an threshold for compound detection^16,63^. The data were searched against HMDB, KEGG, LipidMaps and MzCloud databases for putative metabolite annotation. Compounds with <25% RSD (relative standard deviation) in pooled QC post SERRF (Systematic Error Removal Using Random Forest) normalization were subjected to normalization using internal standard followed by VSN (variance stabilizing normalization)^16,64^. The precursor ion extraction of metabolites from glycolysis, TCA cycle and oxidative phosphorylation was performed on skyline 20.1.0.155 with mass error threshold of ±5 ppm. One-way ANOVA in R was performed to determine differentially abundant compounds among multi-groups, and the *p*-values were adjusted for multiple comparisons using Tukey method. Compounds with *P* < 0.05 were considered statistically significant. Each annotated compound was manually verified for its match to predicted/expected retention time, product ions, mz logic score, ionization preference prior and after the statistical analysis. Only endogenous metabolites were used for data representation and interpretation (The global metabolome profile is available in Supplementary Data 4).

### Seahorse assay

An Agilent Seahorse XF Sensor Cartridge was hydrated in Agilent Seahorse XF Calibrant at 37 °C in a non-CO2 incubator overnight. On the day of assay, each well of a Seahorse XF-96-well cell culture microplate was seeded with 400,000 resting T cells or 200,000 activated T cells [cultured with Dynabeads™ Mouse T-Activator CD3/CD28 (11452D, ThermoFisher) for 24 h] in 180 μl of Seahorse assay medium, consisting of basal XF media, 1 mM sodium pyruvate, 2 mM glutamine, and 20 mM glucose, with a pH of 7.4. Four wells have only Seahorse assay medium without cells as controls. The cell culture plate was thereafter placed in a non-CO2 incubator at 37 °C for 1 h prior to the assay. Stressor mix (1 μM oligomycin and 5 μM FCCP for resting T cells; 1 μM oligomycin and 1 μM FCCP for activated T cells) was subsequently loaded into the hydrated sensor cartridge. The sensor cartridge was then mounted onto the cell culture plate and loaded into the Seahorse XF96 Analyzer (Agilent, Santa Clara, CA), and Agilent Seahorse XF Cell Energy Phenotype Test was performed with simultaneous injection of oligomycin and FCCP.

### Measurement of cytokine and chemokine expression by Luminex assay

To measure cytokine and chemokine levels, 0.5 ml peripheral blood per mouse was collected from *Mir142*^*−/−*^*BCR-ABL* and *Mir142*^*+/+*^*BCR-ABL* mice (BCR-ABL expression was induced by tet-off for 3 weeks), and plasma was obtained and aliquoted. Bone marrow (BM) was flushed out from the two femurs using 0.2 ml IMDM medium. Spleen was weighed, cut into small pieces, and squeezed through a 100 µm cell strainer using 1 ml IMDM medium. BM and spleen cell suspensions were centrifuged, and supernatants were aliquoted and snap frozen in liquid nitrogen. Customized Luminex assay for a panel of murine cytokines and chemokines (R&D systems; Supplementary Table 4) were performed on plasma and BM and spleen supernatants and concentrations of cytokines/chemokines calculated using standard curves, based on the manufacturer’s protocol. The volume of the two femur cavities is about 20 μl, and the measured concentrations were adjusted based on this dilution factor (200/20 = 10×) to obtain the concentrations of cytokines/chemokines per unit volume BM. Concentrations in splenic supernatants were normalized to weight.

### Western blotting

T cells were lysed in the RIPA buffer (Thermal Fisher) with protease inhibitor cocktail (Sigma Aldrich). The protein concentration of the lysates was quantified by the Bradford protein assay kit (Bio-Rad). Equal amounts of protein (20 μg) were electrophoresed in 12% sodium dodecyl sulfate-polyacrylamide gel and transferred to polyvinyldifluoride membranes (Millipore). The membranes were blocked for 1 h with 5% non-fat milk, and incubated with primary Abs (TGFBR1, ab235578, Abcam; TGFBR2, ab269279, Abcam; PD-1, AF1021, R&D; ACTIN, 8H10D10, Cell Signaling) diluted by 1:1,000 in 5% non-fat milk at 4 °C overnight. For NFATC1, the membrane was blocked for 1 h with 5% BSA and incubated with primary Ab (NFAT2, #8032, Cell Signaling) diluted by 1:1000 in 5% BSA. After washing, membranes were incubated with the corresponding horseradish peroxidase-conjugated secondary Ab diluted by 1:2000 in 5% non-fat milk. The immune complexes were visualized using SuperSignal™ West Femto Maximum Sensitivity Substrate detection kit according to the manufacturer’s instructions (ThermoFisher). When necessary, membranes were stripped by stripping buffer (Bioland Scientific) for 10 min at room temperature, and re-probed with Abs.

### Lentiviral transduction of human T cells

GFP-expressing miRZip anti-miR-142-3p/5p (CS940MZ-1, a custom order from System Biosciences, with H1 promoter for anti-miR-142-3p and EF1a promoter for GFP-T2A-Puro expression) and non-target control (cat: SI506A-1, from System Biosciences) lentiviruses were produced and used for transduction of human T cells. Briefly, human T cells were activated using Dynabeads™ Human T-Activator CD3/CD28 (11131D, ThermoFisher) with a ratio of 3:1 in 24-well culture plate in the presence of 30 μ/ml IL-2. On the second day, cells were transferred into RetroNectin-coated plate followed by addition of lentivirus with a MOI of 3 and TransDux virus transduction reagent (System Biosciences). The plate was then centrifuged for 90 min at 1500 × *g* for spinoculation. After 3 days of culturing, GFP positive cells were sorted, and then analyzed for apoptosis, cell cycling, cell growth and intracellular cytokines.

### Oligodeoxynucleotide design and synthesis

MiR-142-3p inhibitor and miR-142-5p inhibitor (collectively called miR-142-3p/5p inhibitor) and miR-142-3p and -5p mimics (collectively called M-miR-142) were designed and synthesized as previously stated^33,34^. The double-stranded sequence of miR-142 mimic was conjugated through the 5′ end of the passenger strand to the 3′ end of a single-stranded, partly phosphorothioated oligodeoxynucleotide (CpG ODN) using a synthetic carbon linked to obtain CpG-M-miR-142. To ensure the maximum activity and target specificity and to confer nuclease resistance to M-miR-142, the miR-142 moiety was also 2′-O-methyl modified at the 3′ end of the passenger strand. The constructs were also conjugated with Cy3 to track the internalization in the cells by flow cytometry. The sequences were as follows: miR-142-3p inhibitor: 5′ G*G*T GCA TCG ATG CAGG*G*G* G*G xxxxx mUmCmC mAmUmA mAmAmG mUmAmG mGmAmA mAmCmA mCmUmA mCmA-3′; miR-142-5p inhibitor: 5′- G*G*T GCA TCG ATG CAG G*G*G* G*G/iSpC3//iSpC3//iSpC3//iSpC3//iSpC3/mA mGmUmA mGmUmG mCmUmU mUmCmU mAmCmU mUmUmA mUmG -3′; miR-142-3p mimic (guide): 5′- rUrGrU rArGrU rGrUrU rUrCrCrUrArC rUrUrU rArUrG rGrA -3′; CpG-miR-142-3p mimic (passenger): 5′- G*G*T GCA TCG ATG CAG G*G*G* G*G xxxxx rC rArUrA rArArGrUrArG rGrArA rArCrA rCrUrA rCrAmA rA-3′; miR-142-5p mimic (guide): 5′- rCrArU rArArA rGrUrA rGrArA rArGrC rArCrU rArCrU -3′; CpG-miR-142-5p mimic (passenger): 5’- G*G*T GCA TCG ATG CAG G*G*G* G*G/iSpC3//iSpC3//iSpC3//iSpC3//iSpC3/rU rArGrU rGrCrU rUrUrC rUrArC rUrUrU rArUrG mArA -3′; Scramble RNA (scrRNA, guide): 5′-rGrGrCrGrUrGrUrArUrUrArArGrGrCrUrArArArUrCrU-3′; CpG-scrRNA (passenger): 5′-G*G*T GCATCGATGCAGG*G*G*G*G xxxxx rArUrUrUrArGrCrCrUrUrArArUrArCrArCrGrCrCmArA-3′, where ‘r’ indicates ribo, ‘*’ indicates phosphorothioation, one nonbridging atom of oxygen on phosphate was replaced with sulfur, ‘x’ indicates a C3 Spacer, and ‘m’ indicates the 2′-O-methyl analog of the nucleotide. The annealed miR-142-3p mimic is as follows:
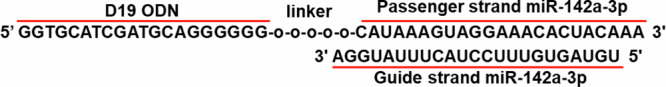


The annealed miR-142-5p mimic is as follows:
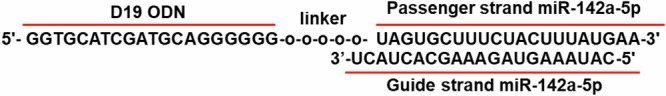


### In vivo treatment of mice

To evaluate the impact of upregulating miR-142 in T cells by the homemade drug M-miR-142 on T cell antileukemic activity in vivo, we performed in vivo experiments and delivered M-miR-142 to the mice by retroorbital injection (i.e., 20 mg/kg miR-142-3p mimic and 10 mg/kg miR-142-5p mimic, hereafter called collectively 30 mg/kg M-miR-142). We chose these doses based on our previous experience working with miRNA mimics and the expression levels of miR-142-3p vs -5p in T cells (higher base levels of miR-142-3p vs -5p). We first co-transplanted *Mir142*^*−/−*^*BCR-ABL* LSKs (CD45.2) and *Mir142*^*−/−*^ T cells (CD45.2) into NSG mice (CD45.1, lacking T cells) and then treated these recipient mice with M-miR-142 or SCR (30 mg/kg/day, iv) for 3 weeks, starting on day 3 after transplantation, and monitored them for leukemic burden and survival. To determine if M-miR-142 could rescue miR-142 deficit and T cell hypofunction and therefore inhibit BC evolution, a cohort of *Mir142*^*−/−*^*BCR-ABL* mice were treated with M-miR-142 or SCR (30 mg/kg/day, iv), starting on day 15 after tet-off BCR-ABL induction, for 3 weeks. T cells in PB, BM and spleen, leukemic blasts, and survival were monitored. A 2nd transplant experiment was also performed by transplanting BM cells from the treated mice into 2nd recipient mice to evaluate post-treatment LSC burden. To study the effect of M-miR-142 on human T cells, we generated BC CML PDX model by transplanting human BC CML CD34^+^ cells into NSGS (2Gy, The Jackson Laboratory) mice. Two weeks after transplantation, we detected >5% circulating human CD45^+^ cells and gave each mouse autologous human T cells (T cells from the same patient, expanded by Dynabeads™ Human T-Activator CD3/CD28 for 2 weeks, 10^6^ T cells respectively on day 14 and day 21 post transplantation), and M-miR-142 or SCR (30 mg/kg/day, iv, starting on day 15 after transplantation, for 3 weeks). A cohort of mice were monitored for T cells and leukemic blasts and survival, and another cohort were used for 2nd transplant to evaluate post-treatment LSC burden.

Since TKIs represent a first-line targeting approach for CML, to test if targeting miR-142 deficit and/or the consequential PD-1 upregulation would enhance TKI activity in BC models, we generated a cohort of BC CML mice with a similar leukemia onset time by transplanting BM cells (from both tibias and femurs) from diseased *Mir142*^*−/−*^*BCR-ABL* mice (CD45.2, BCR-ABL was induced by tet-off for 3 weeks) into congenic normal wt recipient mice (CD45.1). On day 15 after transplantation, we treated these mice with TKI (nilotinib; NIL, 30 mg/kg/day, oral gavage), TKI+M-miR-142 (30 mg/kg/day, iv), TKI+PD-1 blocking Ab (10 mg/kg, ip, 3x/week), TKI+M-miR-142 + PD-1 Ab (“triplet”), or vehicle for 3 weeks. Leukemic burden, host T cells, and survival were monitored. A 2nd transplant experiment was also performed to evaluate post-treatment LSC burden. A cohort of BC CML PDX mice were also generated by transplanting 10^6^ CD34^+^ cells from BC CML patients into NSGS mice (2 Gy). Two weeks after transplantation, we detected >5% circulating human CD45^+^ cells and gave each mouse autologous human T cells (10^6^ on day 14 post transplantation; only one dose of human T cells was administrated to NSGS mice to avoid GVHD), and NIL (30 mg/kg/day, oral gavage), NIL+M-miR-142 (30 mg/kg/day, iv), NIL+PD-1 blocking Ab (10 mg/kg, ip, 3x/week), “triplet”, or vehicle for 3 weeks. A 2nd transplant experiment was also performed to evaluate post-treatment LSC burden. Human CD45^+^ cell engraftment rates and survival were monitored both in the primary treated and the 2nd recipient mice.

### Statistics and reproducibility

All statistical analyses were performed using Prism version 10 software (GraphPad Software) and SAS version 9.4 (SAS Institute). For all animal experiments, numbers of biologically independent mice were provided. For immunoblotting experiments, results from one of three independent experiments are shown. For all in vitro experiments, numbers of biologically independent samples used in each experiment are provided. Randomization was used for all animal experiments. For some experiments, investigators were blinded to mouse genotype while performing treatment or monitoring for engraftment or survival. For the remaining experiments, the investigators were not blinded to allocation during experiments and outcome assessment. All data were summarized by descriptive statistics. For continuous variables, the difference between two independent groups was assessed by two-tailed, unpaired *t*-test. Differences among multiple independent groups were assessed by one-way ANOVA models. For overall survival data, Kaplan–Meier method and log-rank tests were used to estimate and compare two or more survival curves. Sample size was determined based on daily laboratory practice. No data were excluded from the analyses. *P* values were corrected for multiple comparisons by Holm–Šídák method. For all cases, statistical significance was set as *p* < 0.05. Results shown represent mean ± standard error of the mean (SEM).

### Reporting summary

Further information on research design is available in the Nature Portfolio Reporting Summary linked to this article.

## Supplementary information

Supplementary Information

Description of Additional Supplementary Files

Supplementary Data 1

Supplementary Data 2

Supplementary Data 3

Supplementary Data 4

Reporting Summary

Transparent Peer Review file

## Source data

Source Data

## Data Availability

The RNA-seq (GSE261798) and scRNA-seq (GSE254285 for mouse cells and GSE276130 for human cells) data generated in this study are available at the Gene Expression Omnibus (GEO) repository of the National Center for Biotechnology Information. Metabolomic profiles are available at the NIH Common Fund’s National Metabolomics Data Repository (NMDR) website [http://dev.metabolomicsworkbench.org:22222/data/DRCCMetadata.php?Mode=Study&StudyID=ST003091&Access=HieS7992, The DOI for this project (PR001920) is: 10.21228/M8JB09], the Metabolomics Workbench^65^. Supplementary information, including Supplementary Figs. and legends, Supplementary Tables, Supplementary Data, and Source data are provided with the online version of this paper. Source data are provided with this paper.
