# Supplementary material for: miR-142 deficit in T cells during blast crisis promotes chronic myeloid leukemia immune escape
Source: Nat Commun. 2025 Feb 1;16:1253. doi: 10.1038/s41467-025-56383-y (PMC11787332; doi:10.1038/s41467-025-56383-y)
Supplement: Supplementary file 1 — Supplementary Information [file 41467_2025_56383_MOESM1_ESM.pdf]

miR-142 deficit in T cells during blast crisis promotes chronic myeloid leukemia immune escape

Inventory of Supplementary Information

Supplementary figure 1
Supplementary figure 2
Supplementary figure 3
Supplementary figure 4
Supplementary figure 5
Supplementary figure 6
Supplementary figure 7
Supplementary figure 8
Supplementary figure 9
Supplementary figure 10
Supplementary figure 11
Supplementary figure 12
Supplementary figure 13
Supplementary figure 14
Supplementary figure 15
Supplementary figure 16
Supplementary figure 17
Supplementary Figure 18
Supplementary Table 1
Supplementary Table 2
Supplementary Table 3
Supplementary Table 4

Supplementary Fig. 1

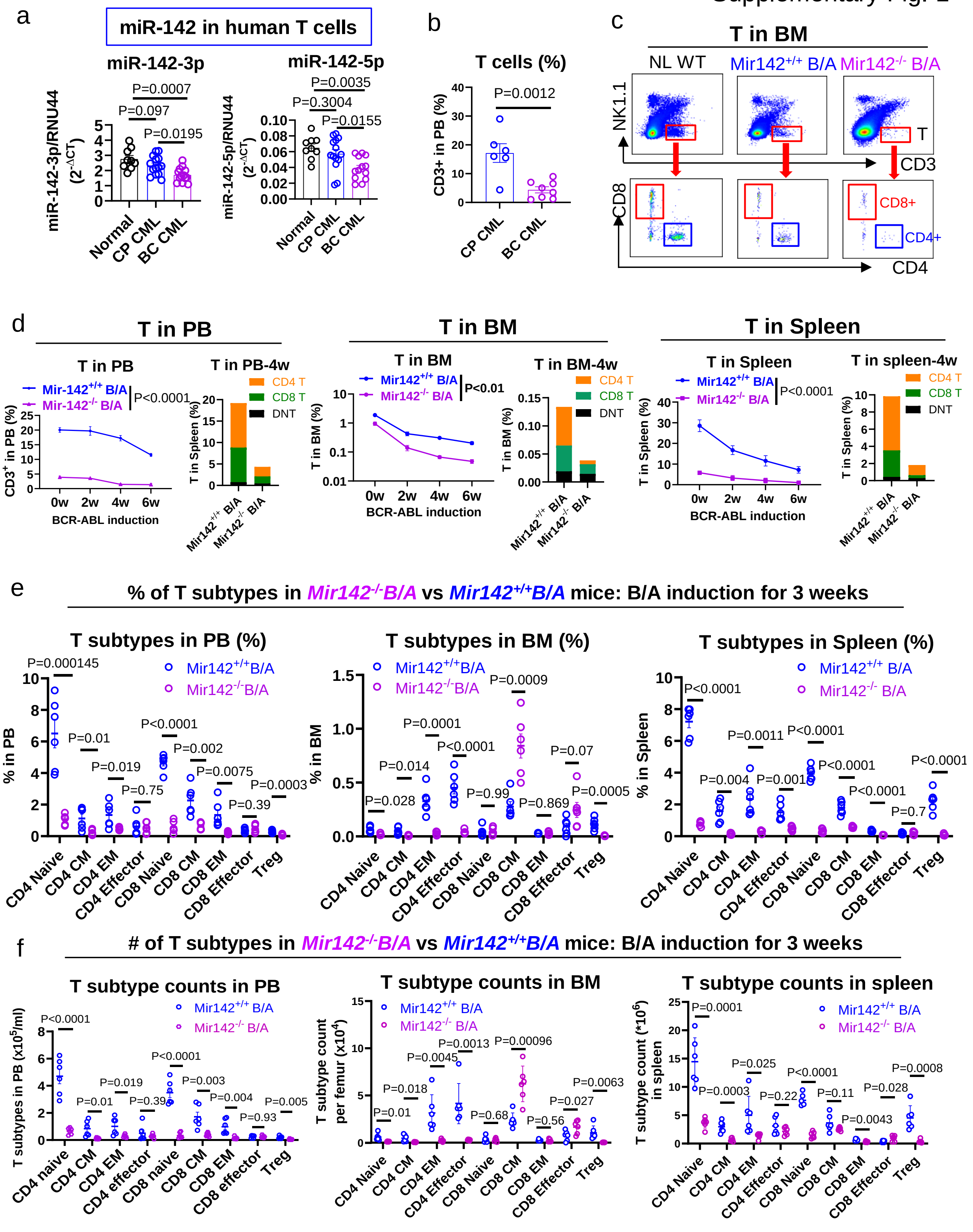

Supplementary Fig. 1: *Mir142$^{-/-}$BCR-ABL* mice exhibit significant T lymphopenia compared with *Mir142$^{+/+}$BCR-ABL* mice. a. Levels of miR-142-3p and -5p in human T cells collected from healthy donors (normal; 3p: n=7 samples; 5p: n=9 samples), CP CML (3p: n=18 samples; 5p: n=14 samples) and BC CML (n=14 samples for both) patients, analyzed by Q-RT-PCR. **b.** Frequencies of T cells in PB samples from CP CML (n=6 samples) or BC CML (n=8 samples) patients, analyzed by flow cytometry. **c**. Representative plots of CD3+, CD3+CD4+, and CD3+CD8+ T cells in BM MNCs from normal wt, *Mir142$^{+/+}$BCR-ABL* (n=11) and *Mir142$^{-/-}$BCR-ABL* (n=9) mice (BCR-ABL were induced by tet-off for 4 weeks), analyzed by flow cytometry. **d.** Percentages of CD3+ (T), CD3+CD4+ (CD4 T), CD3+CD8+ (CD8 T), and CD3+CD4-CD8- (DNT) cells in PB (n=9 each), BM (n=7 each) and spleen (n=6 each) from *Mir142$^{+/+}$BCR-ABL* and *Mir142$^{-/-}$BCR-ABL* mice before (0w) and after BCR-ABL induction by tet-off (2w, 4w and 6w), analyzed by flow cytometry. **e-f**. Percentages (**e**) and absolute numbers (**f**) of CD4+ and CD8+ T cell subpopulations in PB, BM and spleen of *Mir142$^{-/-}$BCR-ABL* versus *Mir142$^{+/+}$BCR-ABL* mice (BCR-ABL were induced by tet-off for 3 weeks). Abbreviation: CP: chronic phase; BC: blast crisis; CML: chronic myeloid leukemia; *B/A*: *BCR-ABL*; MNC: mononuclear cells; tet: tetracycline; PB: peripheral blood; BM: bone marrow; CM: central memory; EM: effector memory; Treg: regulatory T cells. For **a**, comparisons among multi-groups were performed by one-way ANOVA and P values were corrected for multiple comparisons using Holm-Šídák method. For **b** and **d-f**, comparison between two groups was performed by two-tailed, unpaired t-test. Results shown represent mean ± standard error of the mean (SEM). Source data are provided as a Source Data file.

Number of T subtypes in *Mir142$^{-/-}$B/A* vs *Mir142$^{+/+}$B/A* mice: B/A induction for 3 weeks

Cells	Immunopheotypes	PB	BM	Spleen
**CD4 Naïve**	CD3+CD4+CD44-CD62L+	↓	↓	↓
**CD4 CM**	CD3+CD4+CD44+CD62L+	↓	↓	↓
**CD4 EM**	CD3+CD4+CD44+CD62L-IL-7Rα+	↓	↓	↓
**CD4 Effector**	CD3+CD4+CD44+CD62L-IL-7Rα-	=	↓	=
**CD8 Naïve**	CD3+CD8+CD44-CD62L+	↓	=	↓
**CD8 CM**	CD3+CD8+CD44+CD62L+	↓	↑	=
**CD8 EM**	CD3+CD8+CD44+CD62L-IL-7Rα+	↓	=	↓
**CD8 Effector**	CD3+CD8+CD44+CD62L-IL-7Rα-	=	↑	↑
**Treg**	CD3+CD4+CD25+Foxp3+	↓	↓	↓

Supplementary Fig. 2: Table showing the changes of the absolute numbers of CD4 and CD8 naïve, central memory, effector memory, effector, and regulatory T cells in PB, BM (per femur) and spleen from *Mir142$^{-/-}$BCR-ABL* mice compared with the counterparts from *Mir142$^{+/+}$BCR-ABL* mice (BCR-ABL were induced by tet-off for 3 weeks). ↓ means reduced; ↑ means increased; = means no change. Abbreviation: *B/A*: *BCR-ABL*; CM: central memory; EM: effector memory; Treg: regulatory T cells; PB: peripheral blood; BM: bone marrow; tet: tetracycline.

Supplementary Fig. 3

Hallmark GSEA of **scRNA-seq**: *Mir142*$^{-/-}$ *B/A* LMPP vs *Mir142*$^{+/+}$ *B/A* LMPP

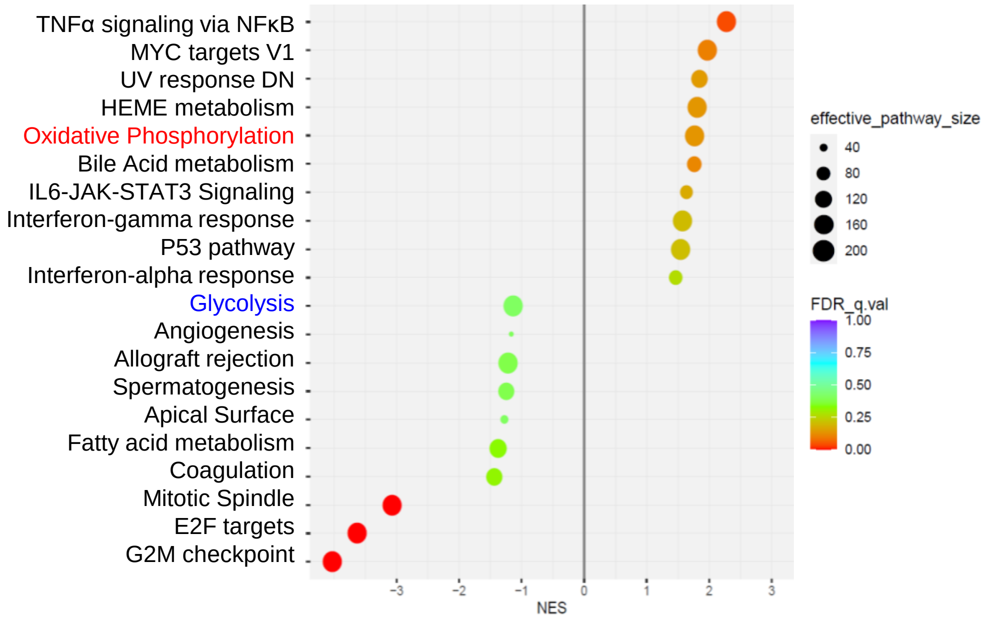

Supplementary Fig. 3: Single cell RNA-seq analysis identifies enrichment of the hallmark gene sets involved in OxPhos in *Mir142*$^{-/-}$*BCR-ABL* LMPPs versus *Mir142*$^{+/+}$*BCR-ABL* LMPPs. GSEA of scRNA-seq data showing the top 10 upregulated and downregulated hallmark gene sets in LMPPs from *Mir142*$^{-/-}$*BCR-ABL* mice versus LMPPs from *Mir142*$^{+/+}$*BCR-ABL* mice (BCR-ABL were induced by tet-off for 3 weeks). Abbreviation: GSEA: gene set enrichment analysis; *B/A*: *BCR-ABL*; LMPP: lymphoid-primed multipotent progenitors; tet: tetracycline; OxPhos: oxidative phosphorylation.

Supplementary Fig. 4

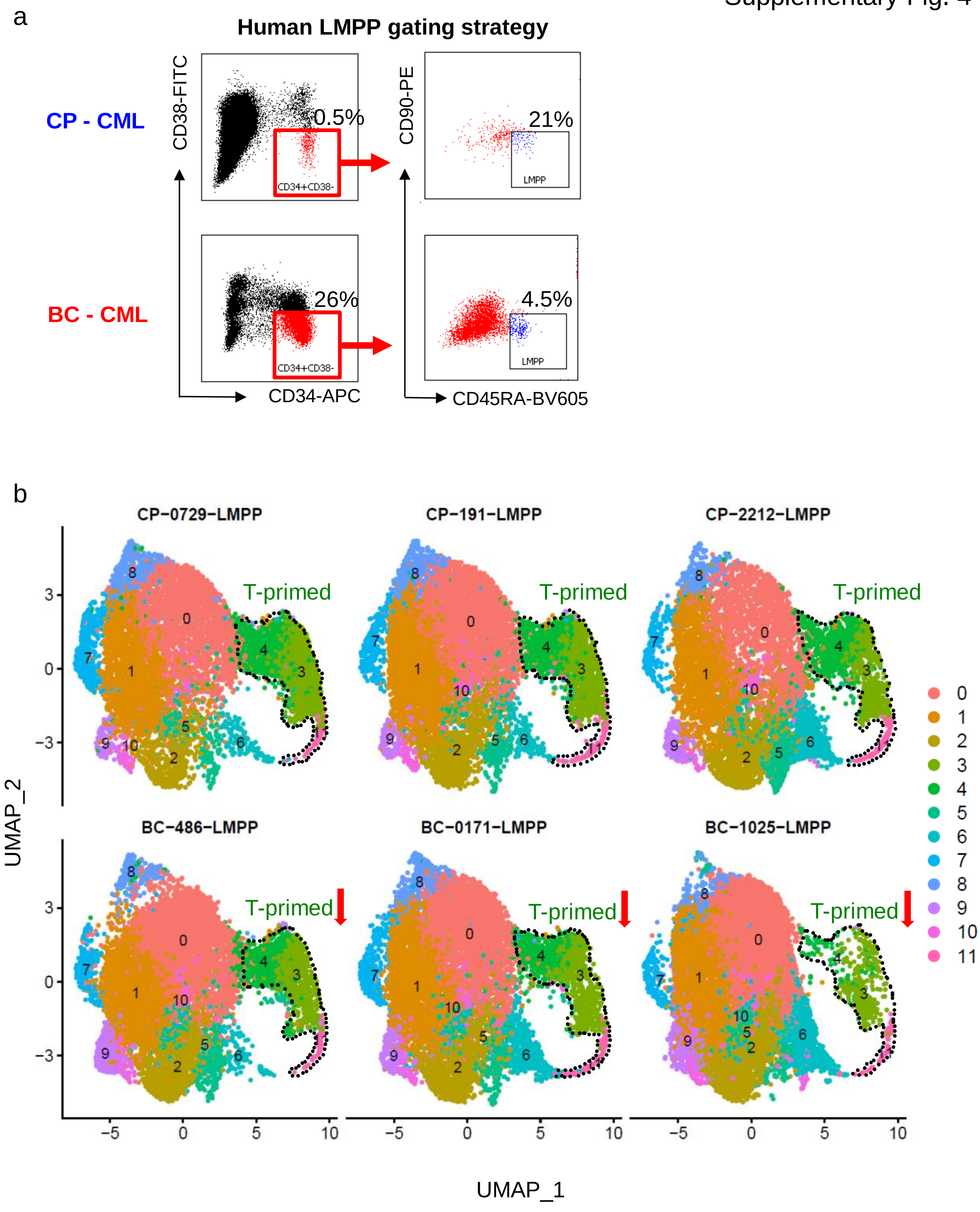

Supplementary Fig. 4: ScRNA-seq analysis of human LMPPs from CP CML and BC CML patients. a. Representative plots of human LMPP gating. Samples from three CP CML patients and three BC CML patients were stained with antibodies and sorted for LMPPs (CD34+CD38-CD45RA+CD90-) using BD Fusion 2 sorter. The human LMPP cells were then subjected to scRNA-seq. **b.** Eleven clusters (C) including T-primed (C3, C4 and C11), Myeloid-primed (C5), and B/T-primed (C7) were identified. Abbreviation: CP: chronic phase; BC: blast crisis; CML: chronic myeloid leukemia; LMPP: lymphoid-primed multipotent progenitors; sc: single cell.

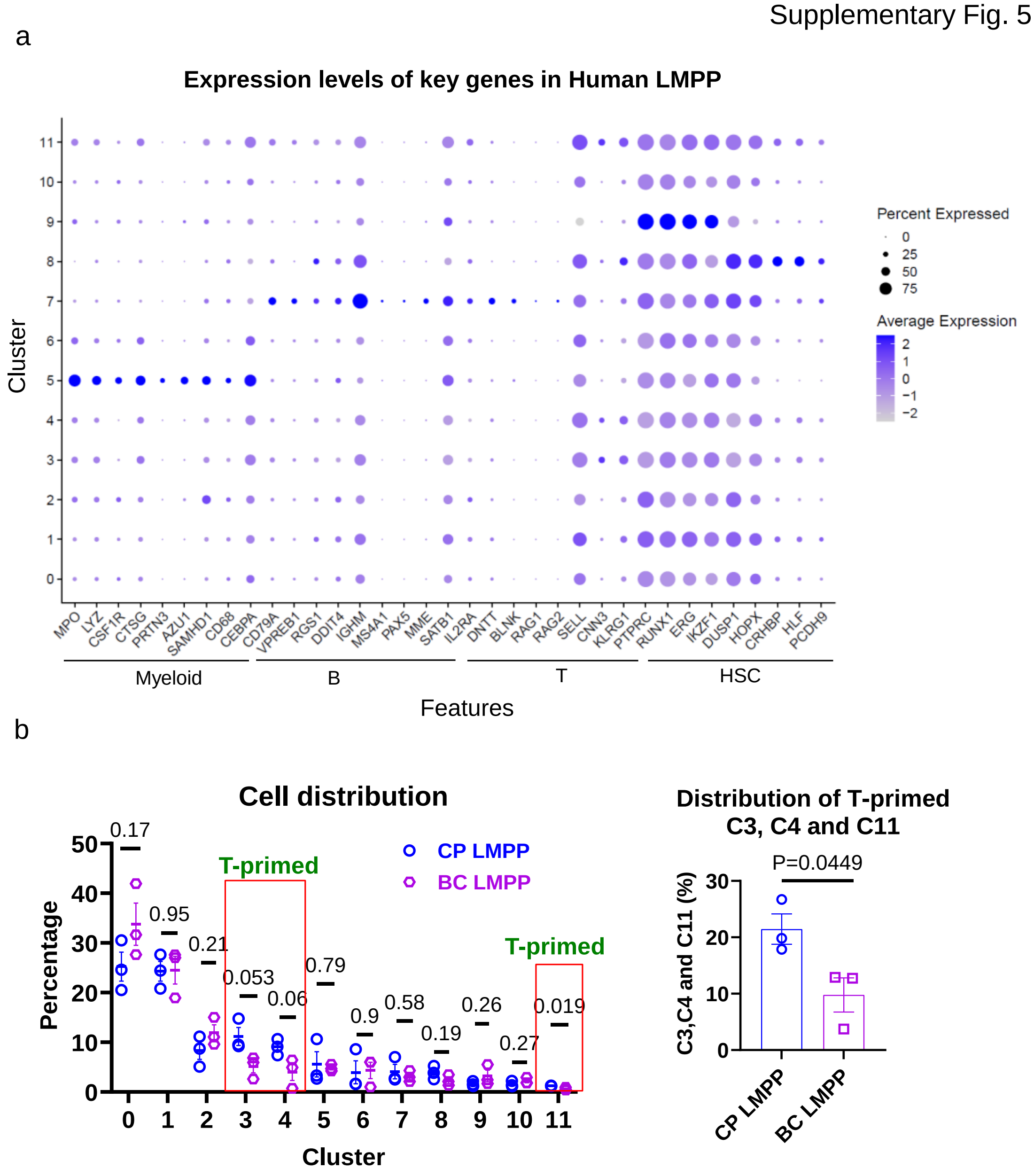

Supplementary Fig. 5: ScRNA-seq analysis of human LMPPs from CP CML and BC CML patients. a. Expression levels of hematopoietic gene transcription factors and cluster differentiation (CD) antigens in the eleven clusters identified by scRNA-seq analysis. **b**. Cell distribution of each cluster (**left**) and of T-primed cluster 3, 4 and 11 (**right**) in human LMPPs isolated from three CP CML patients and three BC CML patients. Abbreviation: sc: single cell; CP: chronic phase; BC: blast crisis; CML: chronic myeloid leukemia; LMPP: lymphoid-primed multipotent progenitors. Comparison between two groups was performed by two-tailed, unpaired t-test. Results shown represent mean ± SEM. Source data are provided as a Source Data file.

Supplementary Fig. 6

a

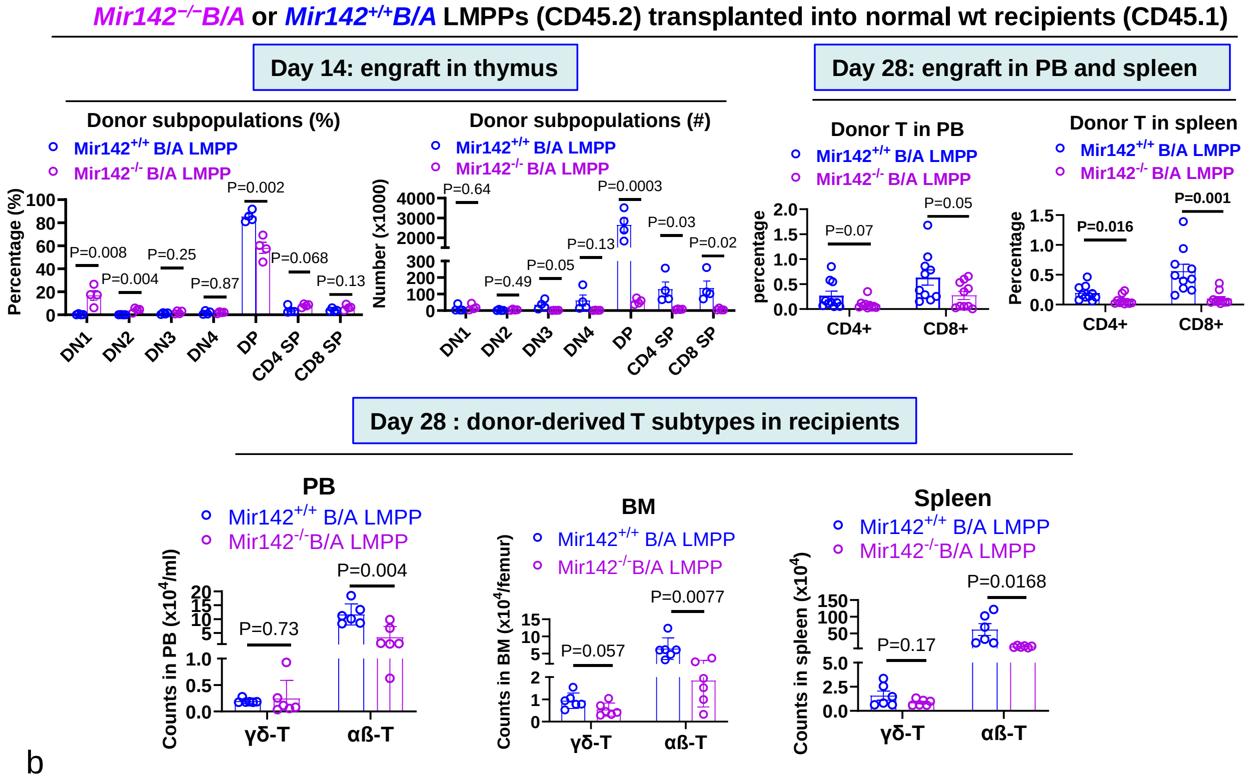

b

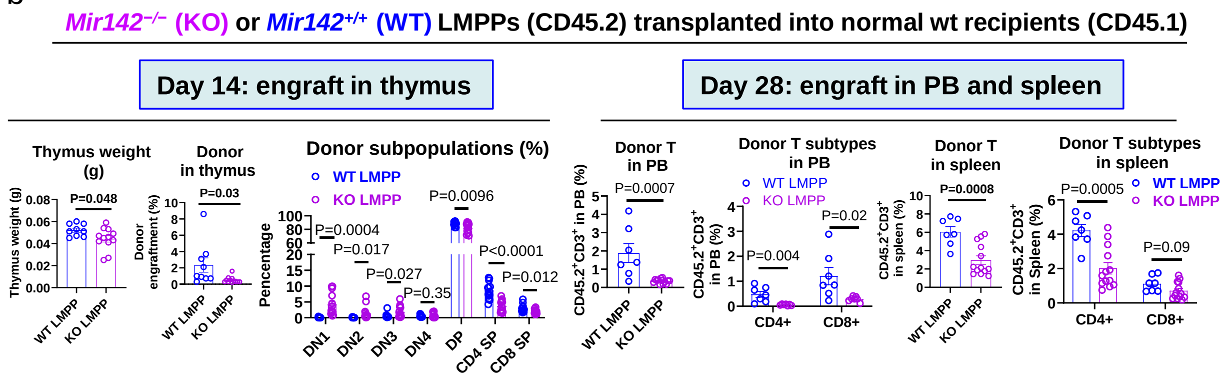

Supplementary Fig. 6: miR-142 deficit redirects LMPPs toward myeloid lineage and impairs T lymphoid differentiation. a. Relative to **Figs. 2e-h**. LMPPs from CD45.2 *Mir142*$^{+/+}$*BCR-ABL* or *Mir142*$^{-/-}$*BCR-ABL* mice (BCR-ABL were induced by tet-off for 3 weeks) were transplanted into congenic CD45.1 wt recipients. After transplantation, percentages and absolute numbers of donor cell subpopulations in thymus on day 14 (upper left panel; n=4 mice per group) and percentages of donor LMPP-derived CD45.2+CD3+CD4+ (donor CD4+ T) and CD45.2+CD3+CD8+ (donor CD8+ T) cells in PB and spleen on day 28 (upper right panel; n=10 mice per group), and absolute numbers of γδ-T and αβ-T lymphocytes in PB, BM and spleen on day 28 (lower panel; n=6 mice per group) were analyzed by flow cytometry**. b**. LMPPs from CD45.2 *Mir142*$^{+/+}$ (WT) or *Mir142*$^{-/-}$ (KO) mice were transplanted into congenic CD45.1 wt recipients. After transplantation, thymus weight and percentages of donor LMPPs-derived cells (CD45.2+) and CD4 and CD8 DN, DP and SP subpopulations in thymus on day 14 (left panel; thymus weight and donor in thymus: n=9 for WT LMPP and n=12 for KO LMPP; donor subpopulations: n=13 per group) and percentages of donor LMPP-derived CD45.2+CD3+ (donor T), CD45.2+CD3+CD4+ (donor CD4+ T), CD45.2+CD3+CD8+ (donor CD8+ T) cells in PB and spleen of the recipient mice on day 28 (right panel; WT LMPP: n=7; KO LMPP: n=7 in donor T subtypes in PB and n=13 in all the other figures) were analyzed by flow cytometry. Abbreviation: *B/A*: *BCR-ABL*; LMPP: lymphoid-primed multipotent progenitors; tet: tetracycline; DN: CD4 and CD8 double negative; DP: CD4 and CD8 double positive; SP: CD4 or CD8 single positive; PB: peripheral blood; BM: bone marrow. Comparison between groups was performed by two-tailed, unpaired t-test. Results shown represent mean ± SEM. Source data are provided as a Source Data file.

Supplementary Fig. 7

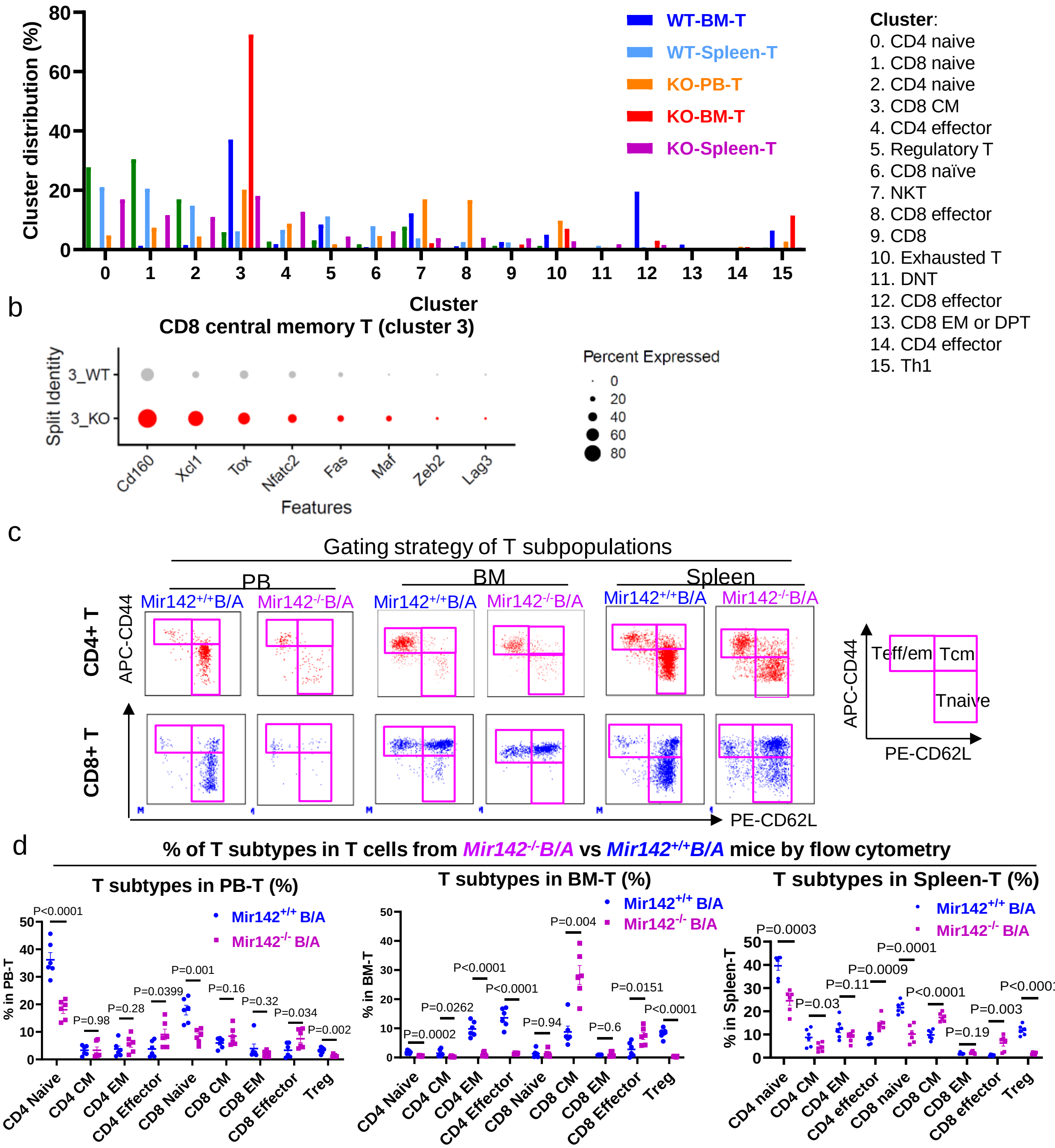

Cells	Immunopheotypes	PB-T	BM-T	Spleen-T
CD4 Naïve	CD3+CD4+CD44-CD62L+	↓	↓	↓
CD4 CM	CD3+CD4+CD44+CD62L+	=	↓	↓
CD4 EM	CD3+CD4+CD44+CD62L-IL-7Rα+	=	↓	=
CD4 Effector	CD3+CD4+CD44+CD62L-IL-7Rα-	↑	↓	↑
CD8 Naïve	CD3+CD8+CD44-CD62L+	↓	=	↓
CD8 CM	CD3+CD8+CD44+CD62L+	=	↑	↑
CD8 EM	CD3+CD8+CD44+CD62L-IL-7Rα+	=	=	=
CD8 Effector	CD3+CD8+CD44+CD62L-IL-7Rα-	↑	↑	↑
Treg	CD3+CD4+CD25+Foxp3+	↓	↓	↓

Supplementary Fig. 7: T cells from *Mir142$^{-/-}$BCR-ABL* mice exhibit reduced naïve T and regulatory T and increased effector T and CD8+ central memory T proportions compared with T cells from *Mir142$^{+/+}$BCR-ABL* mice. a. ScRNA-seq analysis showing distribution of clusters, representing CD4 and CD8 subtypes, in PB-T, BM-T and spleen-T cells collected from *Mir142$^{-/-}$BCR-ABL* (WT) and *Mir142$^{+/+}$BCR-ABL* (KO) mice (BCR-ABL were induced by tet-off for 3 weeks). **b**. Expression levels of exhaustion gene markers in CD8+ central memory T (cluster 3) from *Mir142$^{-/-}$BCR-ABL* (WT) and *Mir142$^{+/+}$BCR-ABL* (KO) mice (BCR-ABL were induced by tet-off for 3 weeks), analyzed by scRNA-seq. **c-d.** Representative plots (**c**) and combined results (**d, top**) of flow cytometry analysis showing percentages of CD4 and CD8 naïve, central memory (CM), effector memory (EM), effector and regulatory T cells in PB-T, BM-T and spleen-T cells from *Mir142$^{-/-}$BCR-ABL* and *Mir142$^{+/+}$BCR-ABL* mice (BCR-ABL were induced by tet-off for 3 weeks, n=6 mice per group). **d, bottom**. Table showing the changes of the percentages of CD4 and CD8 naïve, central memory, effector memory, effector, and regulatory T cells in PB-T, BM-T and spleen-T cells from *Mir142$^{-/-}$BCR-ABL* mice compared with those in PB-T, BM-T and spleen-T from *Mir142$^{+/+}$BCR-ABL* mice (BCR-ABL were induced by tet-off for 3 weeks). ↓ means reduced; ↑ means increased; = means no change. Abbreviation: *B/A*: *BCR-ABL;* tet: tetracycline; PB: peripheral blood; BM: bone marrow; CM: central memory; EM: effector memory; Treg: regulatory T cells. Comparison between groups was performed by two-tailed, unpaired t-test. Results shown represent mean ± SEM. Source data are provided as a Source Data file.

Supplementary Fig. 8

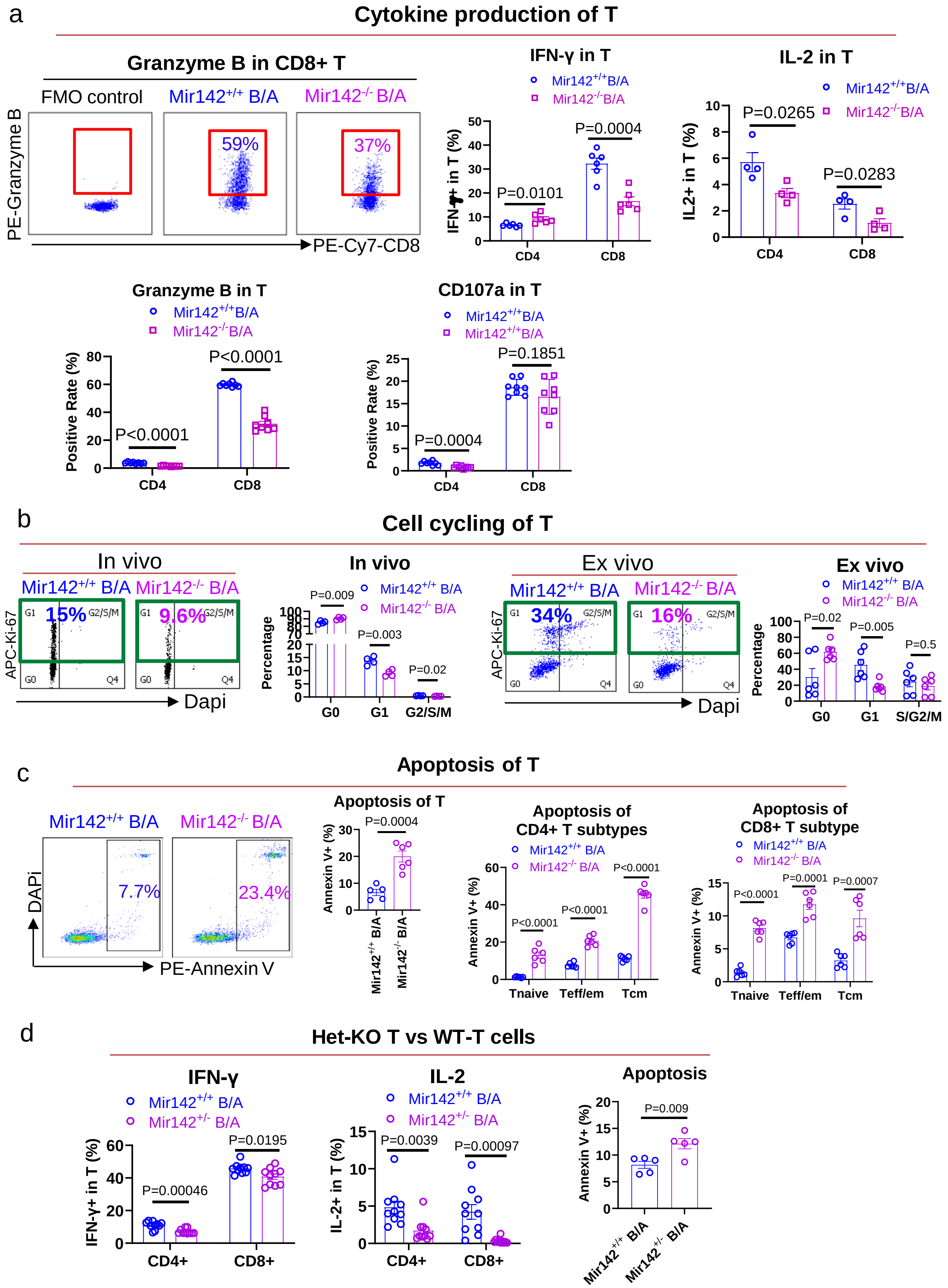

Supplementary Fig. 8: miR-142 deficit impacts T cell cytokine production, cell cycling and survival. a-c. Representative plots and combined results showing levels of intracellular cytokines and cytotoxicity associated proteins including IFN-γ (n=6 per group), IL-2 (n=4 per group), granzyme B and CD107a (n=8 per group; **a**), cell cycling (**b**; in vivo: n=4 per group, T cells were freshly isolated; ex vivo: n=6 per group, T cells were activated by CD3/CD28 Dynabeads for 3 days), and apoptosis (**c**; n=5 for the apoptosis of *Mir142*$^{+/+}$*BCR-ABL* T; n=6 for all the remaining groups) of T cells from the spleen of *Mir142*$^{+/+}$*BCR-ABL* and *Mir142*$^{-/-}$*BCR-ABL* mice (BCR-ABL were induced by tet-off for 3 weeks), analyzed by flow cytometry. **d**. Cytokine (IFN-γ and IL-2) production (n=10 per group) and apoptosis (n=5 per group) of T cells from the spleen of *Mir142*$^{+/+}$*BCR-ABL* and *Mir142*$^{+/-}$*BCR-ABL* mice (miR-142 het KO; BCR-ABL were induced by tet-off for 3 weeks), analyzed by flow cytometry. Abbreviation: *B/A*: *BCR-ABL;* tet: tetracycline; Teff/em: effector and effector memory T; Tcm: central memory T; Het: heterozygous; KO: knock out. Comparison between groups was performed by two-tailed, unpaired t-test. Results shown represent mean ± SEM. Source data are provided as a Source Data file.

Supplementary Fig. 9

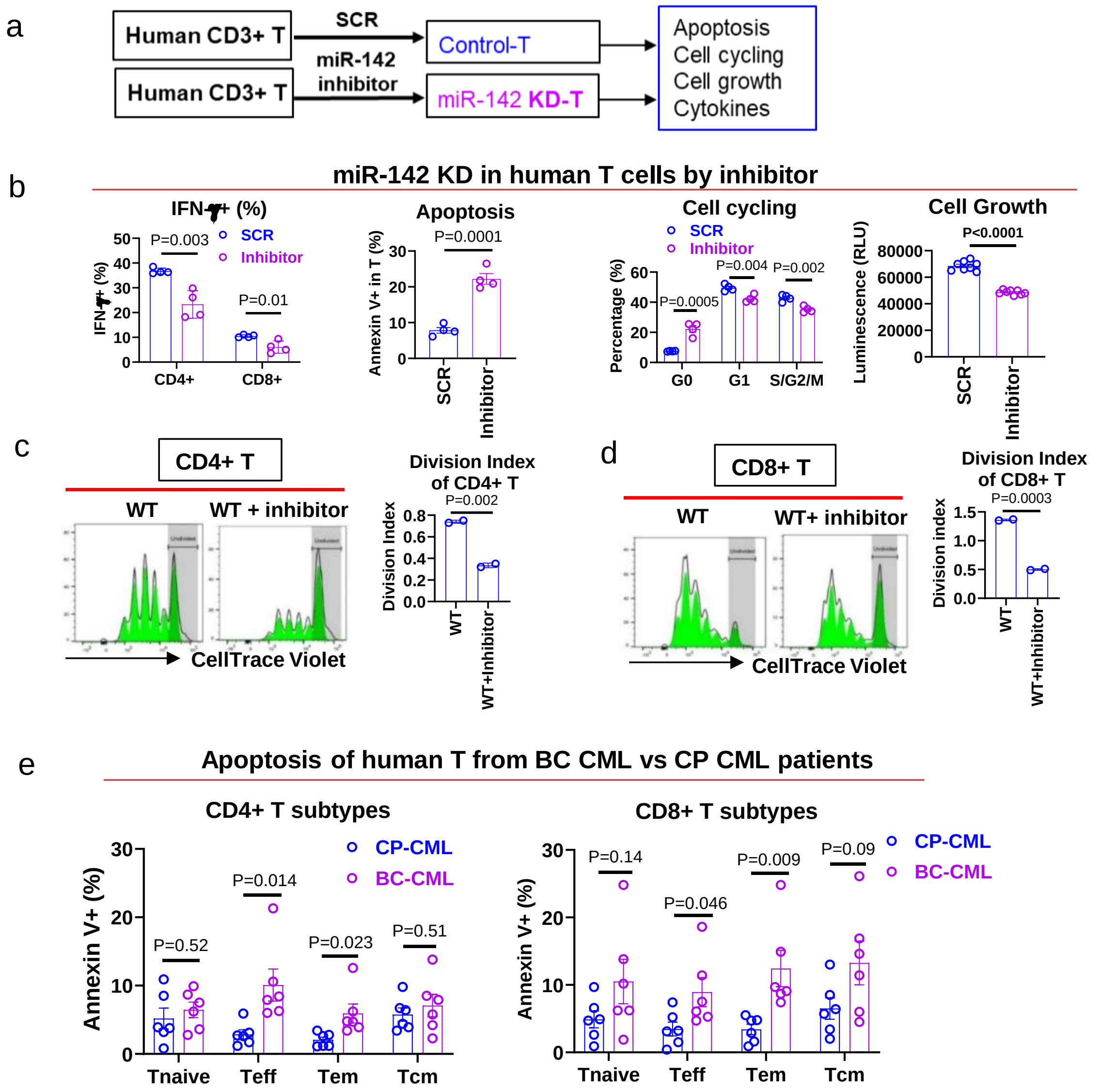

Supplementary Fig. 9: miR-142 deficit impairs *in vitro* T cell activity. a-d. Experimental design and results. Human T cells from healthy donors were treated with scramble RNA or miR-142 inhibitor (2μM) for 3 days (**a**), then levels of intracellular cytokine IFN-γ, apoptosis, and cell cycling were analyzed by flow cytometry (n=4 samples per group) and cell growth (n=8 per group) were measured by Cell-Titer Glo® Luminescent Cell Viability Assay (**b**). T cells were also stained with Celltrace Violet (5uM), activated with CD3/CD28 Dynabeads for 3 days, then CD4+ (**c**) and CD8+ (**d**) T cells were analyzed for cell proliferation by flow cytometry. For **c** and **d**, experiments were repeated twice with similar results. **e**. Apoptosis of CD4+ and CD8+ T cell subpopulations from CP CML and BC CML patients (n=6 samples per group) were analyzed by flow cytometry. Abbreviation: KD: knock down; WT: wild type; CP: chronic phase; BC: blast crisis; CML: chronic myeloid leukemia; Teff: effector T; Tem: effector memory T; Tcm: central memory T. Comparison between groups was performed by two-tailed, unpaired t-test. Results shown represent mean ± SEM. Source data are provided as a Source Data file.

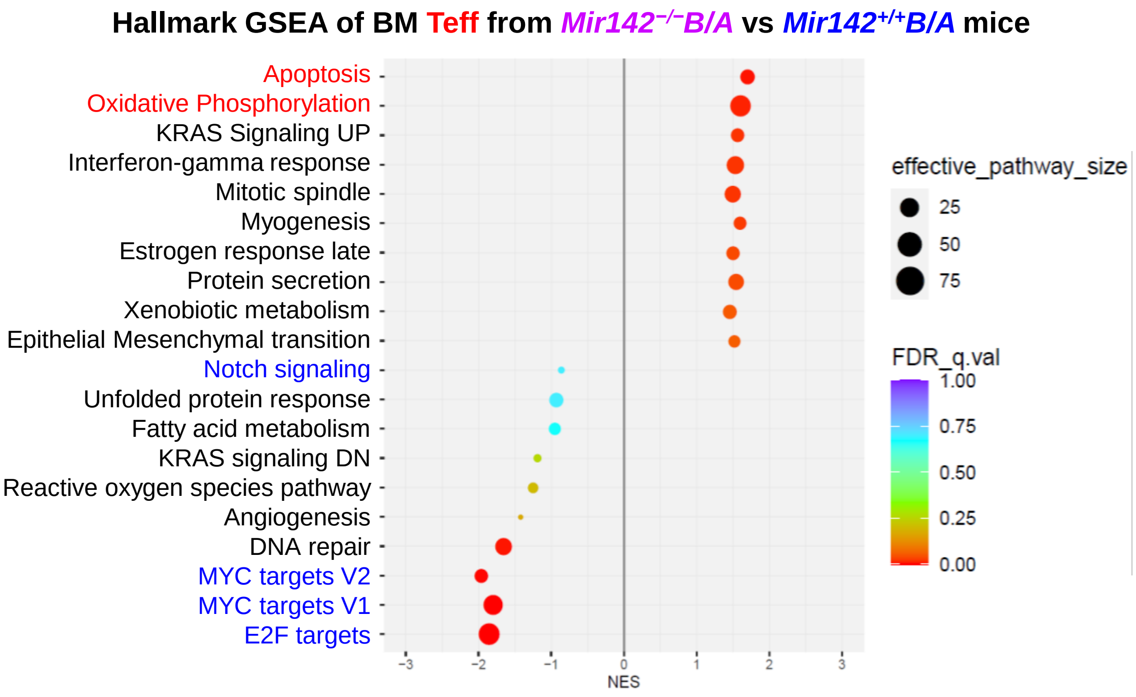

Supplementary Fig. 10: Hallmark gene sets differentially expressed in BM effector T cells from *Mir142*$^{-/-}$*BCR-ABL* versus *Mir142*$^{+/+}$ *BCR-ABL* mice. The top 10 upregulated and downregulated hallmark gene sets in BM effector T cells from *Mir142*$^{-/-}$*BCR-ABL* versus *Mir142*$^{+/+}$*BCR-ABL* mice (BCR-ABL were induced by tet-off for 3 weeks), identified by GSEA of scRNA-seq of T cells. Abbreviation: BM: bone marrow; Teff: effector T; *B/A*: *BCR-ABL*; tet: tetracycline; GSEA: gene set enrichment analysis.

Supplementary Fig. 11

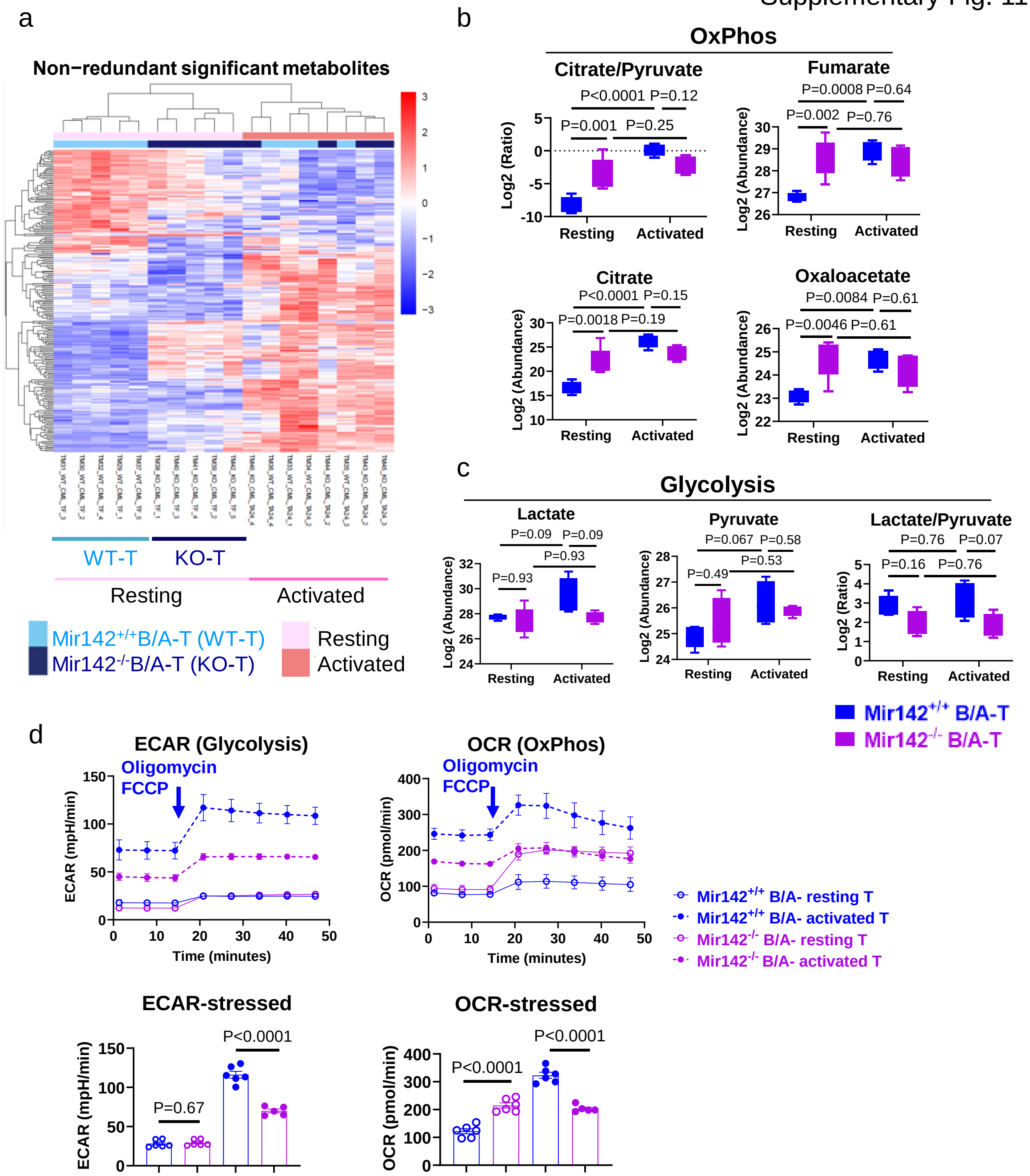

Supplementary Fig. 11: miR-142 deficit impairs T cells' metabolic switch in BC CML. a-c. Unbiased metabolomic profiling of freshly isolated (resting; n=5 per group) and activated (cultured with CD3/CD28 Dynabeads for 24 hours; n=4 per group) T cells from *Mir142*$^{+/+}$*BCR-ABL* (WT-T) or *Mir142*$^{-/-}$*BCR-ABL* (KO-T) mice (BCR-ABL were induced by tet-off for 3 weeks). 216 non-redundant endogenous metabolites that were differentially abundant in resting and activated T cells were identified (**a**). The citrate/pyruvate ratio and abundance of fumarate, citrate, and oxaloacetate, representing OxPhos level (**b**), and the abundance of lactate and pyruvate and lactate/pyruvate ratio, representing glycolysis level (**c**), in *Mir142*$^{+/+}$ *BCR-ABL* T cells versus *Mir142*$^{-/-}$*BCR-ABL* T cells were shown (n=5 per group for resting T; n=4 per group for activated T). **d.** Agilent Seahorse functional assays of ECAR and OCR (**upper**: by Seahorse cell energy phenotype test; **lower**: levels of stressed ECAR and OCR) using resting and activated T cells from *Mir142*$^{+/+}$*BCR-ABL* versus *Mir142*$^{-/-}$*BCR-ABL* mice (BCR-ABL were induced by tet-off for 3 weeks, n=6 mice per group). Abbreviation: *B/A*: *BCR-ABL*; tet: tetracycline; ECAR: extracellular Acidification Rate; OCR: oxygen consumption rate. For **b** and **c**, one-way ANOVA was performed to determine differentially abundant compounds among multi-groups, and P values were adjusted for multiple comparisons using Holm-Šídák method. For **d**, comparison between two groups was performed by two-tailed, unpaired t-test. Results shown represent mean ± SEM. Source data are provided as a Source Data file.

Supplementary Fig. 12

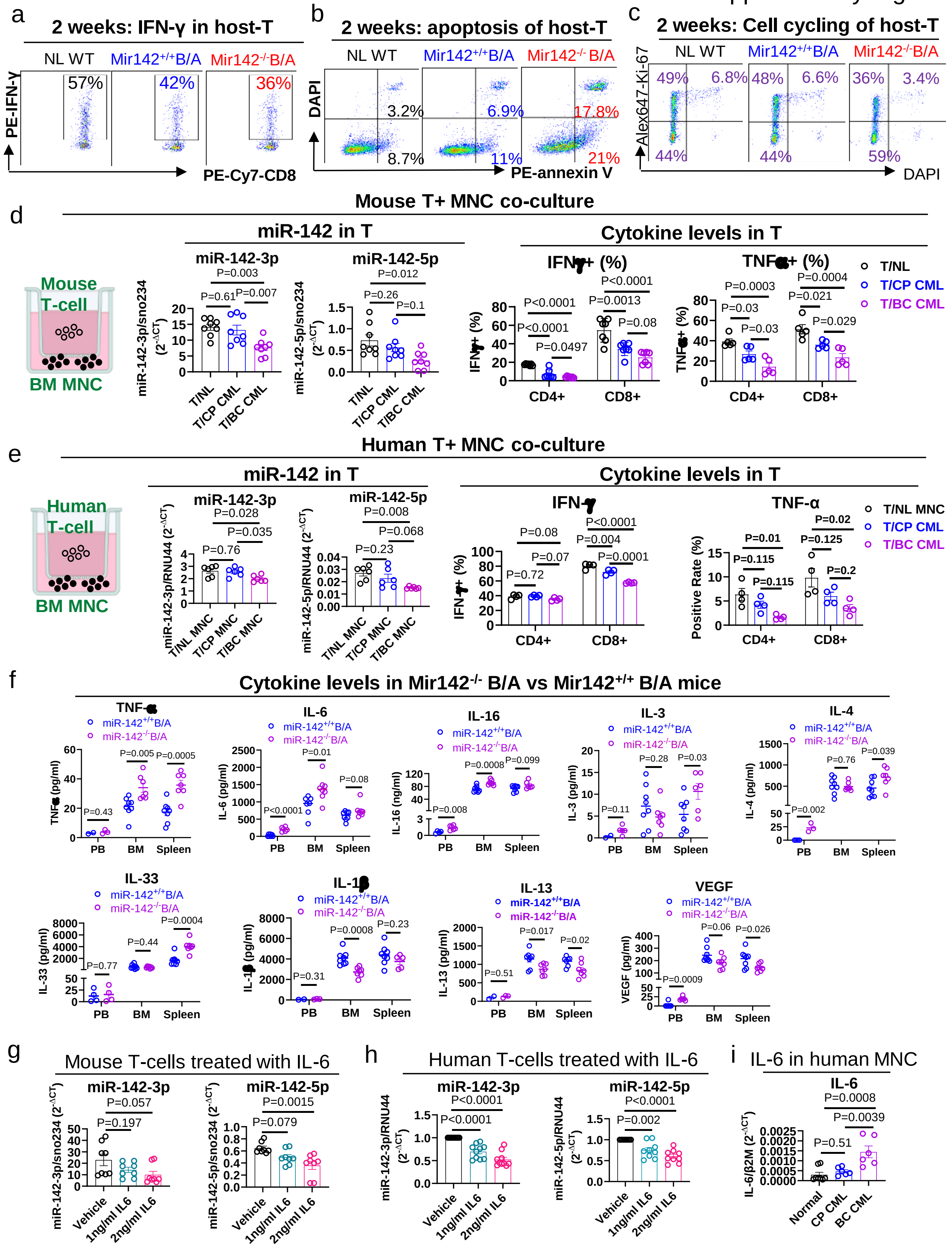

Supplementary Fig. 12: T cell miR-142 deficit is mediated by increased cytokines in BC CML. a-c. Relative to **Fig. 5**. BM MNCs from CD45.2 normal wt mice, *Mir142*$^{+/+}$ *BCR-ABL* and *Mir142*$^{-/-}$*BCR-ABL* mice were transplanted into congenic CD45.1 recipients (10^6/mouse, n=8 per group). Representative plots of IFN-γ production (**a**), apoptosis (**b**), and cell cycling (**c**) of CD45.1+ host-T cells from the recipient mice were analyzed by flow cytometry at 2 weeks after transplantation. **d.** Experimental design and results. Normal wt T cells were co-cultured with BM MNCs from normal wt, *Mir142*$^{+/+}$ *BCR-ABL*, or *Mir142*$^{-/-}$*BCR-ABL* mice in a transwell plate for 3 days, then T cell miR-142 levels by Q-RT-PCR (n=8 per group) and cytokine levels of IFN-γ (n=7 per group) and TNF-α (n=5) by flow cytometry were analyzed. **e.** Experimental design and results. Healthy donor T cells were co-cultured with BM MNCs from healthy donors, CP CML patients, or BC CML patients in a transwell plate for 3 days, then T cell miR-142 levels (n=6 per group) and cytokine levels of IFN-γ and TNF-α (n=4) were analyzed. **f.** Cytokine levels in PB serum and BM and spleen plasma of *Mir142*$^{+/+}$ *BCR-ABL* (n=8) and *Mir142*$^{-/-}$*BCR-ABL* (n=7) mice were analyzed using Luminex assay. **g**. miR-142 levels in mouse T cells treated with 1ng/ml and 2ng/ml IL-6 were analyzed by Q-RT-PCR (n=8 per group). **h**. miR-142 levels in human T cells treated with 1ng/ml and 2ng/ml IL-6 were analyzed by Q-RT-PCR (n=10 for miR-142-3p; n=9 for miR-142-5p). **i**. IL-6 mRNA levels in BM MNCs from healthy donors (n=8), CP CML patients (n=6) or BC CML patients (n=6), analyzed by Q-RT-PCR. Abbreviation: BM: bone marrow; MNC: mononuclear cells; *B/A*: *BCR-ABL*; CP: chronic phase; BC: blast crisis; CML: chronic myeloid leukemia; tet: tetracycline. For **d**, **e**, **g-i**, comparisons among multi-groups were performed by one-way ANOVA and P values were adjusted for multiple comparisons using Holm-Šídák method. For **f**, comparison between two groups was performed by two-tailed, unpaired t-test. Results shown represent mean ± SEM. For **d** and **e**, images created in BioRender. Chen, F. (2025) https://BioRender.com/i04w340. Source data are provided as a Source Data file.

Supplementary Fig. 13

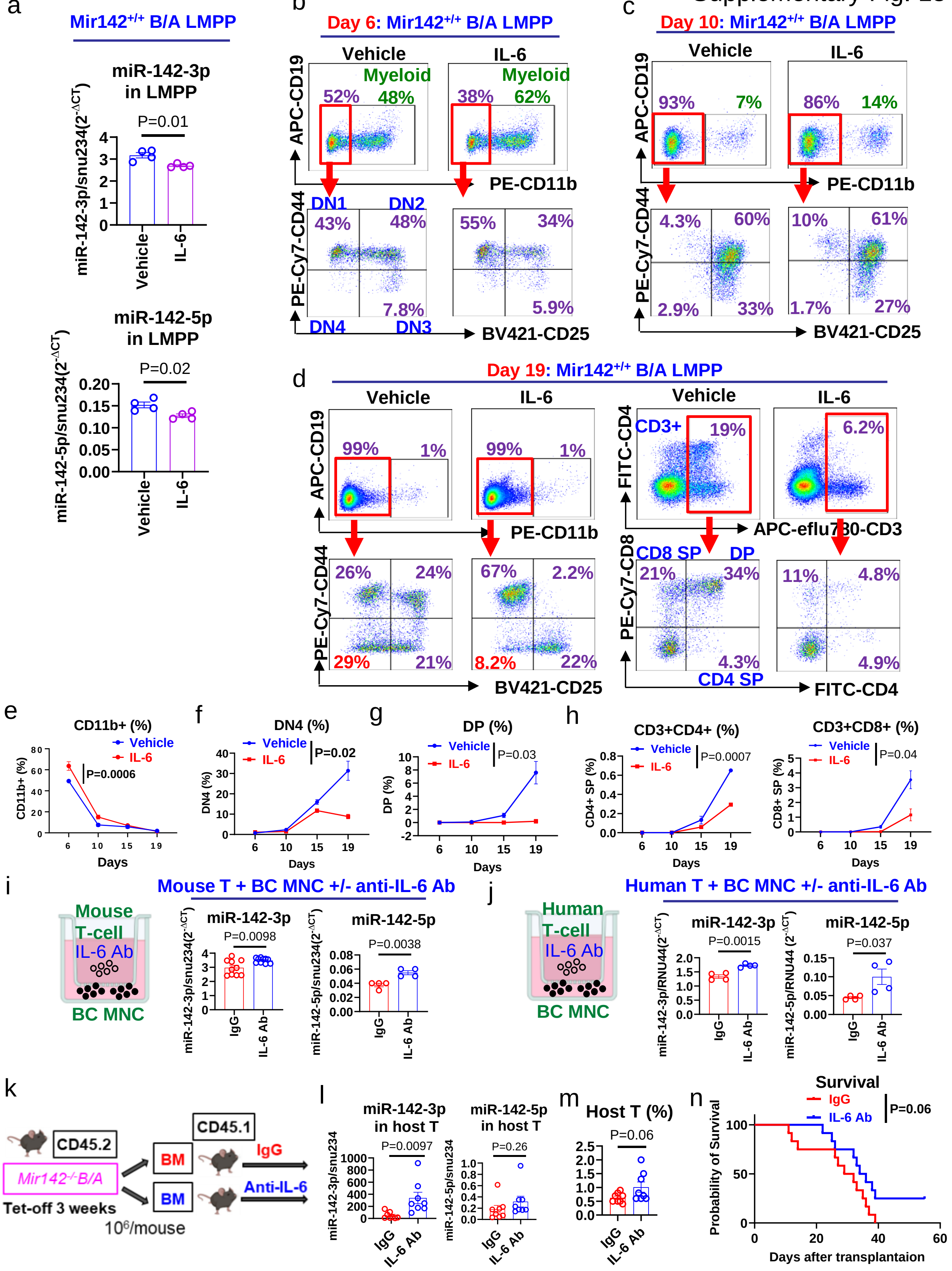

Supplementary Fig. 13: IL-6 redirects LMPP differentiation towards myeloid lineage and impairs T cell differentiation by downregulating miR-142. a-h. LMPPs from the *Mir142*$^{+/+}$ *BCR-ABL* mouse were co-cultured with OP9-DL1 cells and treated with mouse IL-6 (10ng/ml) or vehicle (PBS) for 19 days. MiR-142 levels in LMPPs (n=4 per group) on day 5 were analyzed by Q-RT-PCR (**a**). Representative plots of LMPP-derived subpopulations on day 6 (**b**), 10 (**c**), and 19 (**d**), analyzed by flow cytometry. Percentages of CD11b+ myeloid cells (**e**), DN4 (**f**), DP (**g**), and CD3+CD4+ and CD3+CD8+ mature T (**h**) cells on day 6, 10, 15 and 19 of co-culture are shown. **i.** Mouse T cells were cocultured with BM MNCs from *Mir142*$^{-/-}$*BCR-ABL* mice and treated with IgG or IL-6 blocking Ab (1ug/ml) for 3 days, then miR-142 levels were measured by Q-RT-PCR (n=10 for miR-142-3p; n=4 for miR-142-5p). **j.** Human T cells were cocultured with BM MNCs from BC CML patients and treated with IgG or IL-6 blocking Ab (1ug/ml) for 3 days, then miR-142 levels were measured by Q-RT-PCR (n=4 per group). **k-n**. Experimental design and results. BM cells from the diseased *Mir142*$^{-/-}$*BCR-ABL* mice (CD45.2) were transplanted into CD45.1 congenic recipients (10^6/mouse), and the recipient mice were treated with IgG or IL-6 blocking Ab (100µg, ip, 3x/week) for 3 weeks (**k**). After completion of treatment, levels of miR-142 in the CD45.1+CD3+ host T cells by Q-RT-PCR (**l**; n=8 per group), percentages of host T cells in PB by flow cytometry (**m**; n=8 per group), and survival (**n**; n=12 per group) were monitored. Abbreviation: *B/A*: *BCR-ABL*; LMPP: lymphoid-primed multipotent progenitors; tet: tetracycline; DN: CD4 and CD8 double negative; DP: CD4 and CD8 double positive; SP: CD4 or CD8 single positive; BC: blast crisis; BM: bone marrow; MNC: mononuclear cells; Ab: antibody. Comparison between groups was performed by two-tailed, unpaired t-test. Comparison of survival was performed by Log-Rank test. Results shown represent mean ± SEM. For **i** and **j**, images created in BioRender. Chen, F. (2025) https://BioRender.com/i04w340. For **k**, mouse images created in BioRender. Chen, F. (2025) https://BioRender.com/e61c469. Source data are provided as a Source Data file.

Supplementary Fig. 14

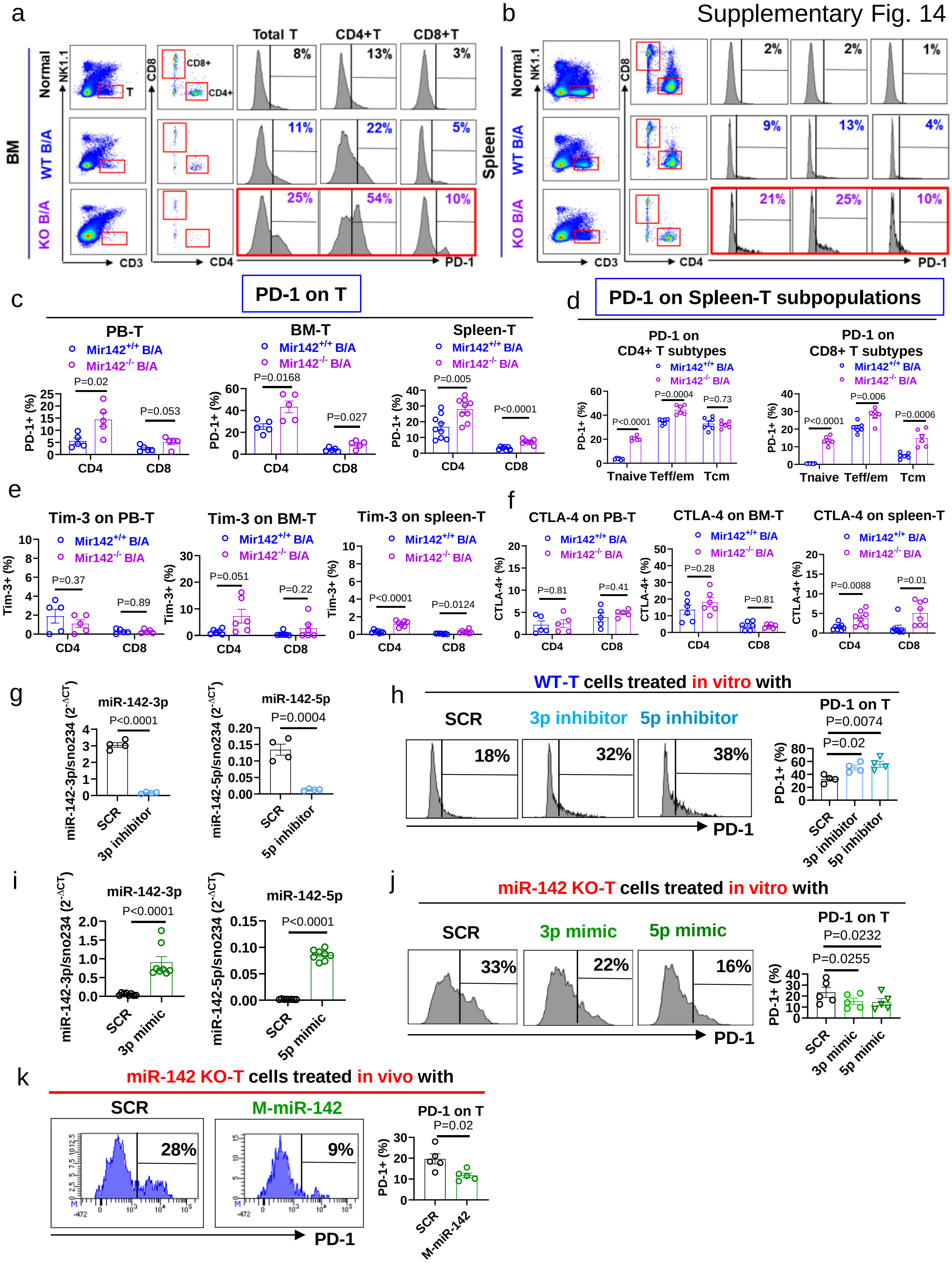

Supplementary Fig. 14: PD-1/PD-L1 axis is upregulated during BC transformation. **a-d.** Representative plots of PD-1 expression on CD3+, CD3+CD4+ and CD3+CD8+ T cells from BM (**a**) and spleen (**b**) and combined results showing percentages of PD-1+ cells in CD4+ and CD8+ T cells from PB (n=5 per group), BM (n=5 per group) and spleen (n=7 for CD4 and n=9 for CD8 per group) of normal wt, *Mir142*$^{+/+}$*BCR-ABL* and *Mir142*$^{-/-}$*BCR-ABL* mice (**c**), and percentages of PD-1+ cells in CD4+ and CD8+ T cell subpopulations from the spleen of *Mir142*$^{+/+}$*BCR-ABL* and *Mir142*$^{-/-}$*BCR-ABL* mice (**d**; n=6 per group), analyzed by flow cytometry. **e-f**. Expression levels of Tim-3 (**e**) and CTLA-4 (**f**) on CD4^{+} and CD8^{+} T cells from PB (n=5 mice per group), BM (n=6 mice per group) and spleen (n=7 for Tim-3 and n=8 for CTLA-4 per group) of *Mir142*$^{-/-}$*BCR-ABL* versus *Mir142*$^{+/+}$*BCR-ABL* mice, analyzed by flow cytometry. **g-h.** Normal wt T cells were treated with SCR or miR-142-3p inhibitor (2μM) or miR-142-5p inhibitor (1μM) for 3 days. Levels of miR-142-3p and miR-142-5p in T cells were quantified by Q-RT-PCR (**g**; n=4 samples per group) and PD-1 expression on T cells was analyzed by flow cytometry (**h**; n=4 samples per group). **i-j**. *Mir142*$^{-/-}$ T cells were treated with SCR or miR-142-3p mimic (2μM) or miR-142-5p mimic (1μM) for 3 days and levels of miR-142-3p and miR-142-5p (**i**; n=8 samples per group) and PD-1 expression (**j**; n=5 samples per group) were analyzed. **k.** *Mir142*$^{-/-}$*BCR-ABL* mice were induced for BCR-ABL induction by tet-off for 1 week, then treated with SCR or M-miR-142 (30mg/kg/day; iv) for 3 weeks, and PD-1 expression on T cells were measured by flow cytometry (n=5 mice per group). Abbreviation: *B/A*: *BCR-ABL*; SCR: scramble RNA; BC: blast crisis; PB: peripheral blood; BM: bone marrow; iv: intravenous injection. For **c-g**, **i** and **k**, comparison between two groups was performed by two-tailed, unpaired t-test. For **h** and **j**, comparisons among multi-groups were performed by one-way ANOVA and P values were adjusted for multiple comparisons using Holm-Šídák method. Results shown represent mean ± SEM. Source data are provided as a Source Data file.

Supplementary Fig. 15

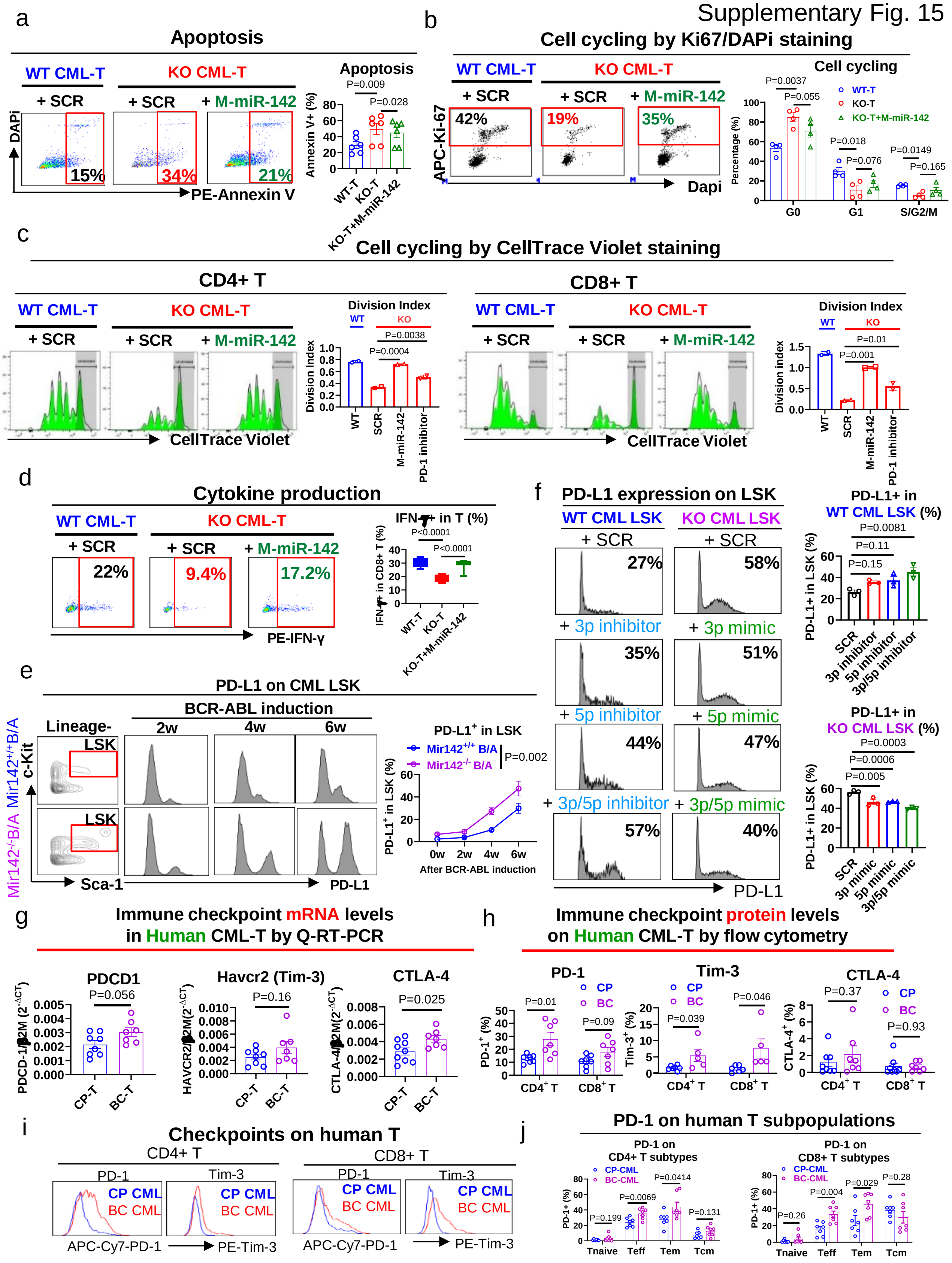

Supplementary Fig. 15: BC CML T cells exhibit high levels of exhaustion markers and defective activity which could be rescued by M-miR-142. a-d. Apoptosis (**a**; n=6 per group), cell cycling by Ki-67/Dapi (**b**; n=4 per group) or CellTrace Violet (**c**; n=2 per group) staining, and levels of cytokine IFN-γ production (**d**; n=8 per group) in *Mir142*$^{+/+}$*BCR-ABL* (WT CML) T cells treated with SCR versus *Mir142*$^{-/-}$*BCR-ABL* (KO CML) T cells treated with SCR or M-miR-142 (2μm, 3 days). **e.** Representative plots and combined results (n=3 per group for 0w and 2w; n=4 per group for 4w; n=5 per group for 6w) of PD-L1 expression on BM LSKs from *Mir142*$^{-/-}$BCR-ABL versus *Mir142*$^{+/+}$BCR-ABL mice after 2, 4 and 6 weeks of BCR-ABL induction. **f.** Representative plots and combined results of PD-L1 expression on *Mir142*$^{+/+}$*BCR-ABL* LSK cells treated with SCR or miR-142-3p or/and miR-142-5p inhibitor, and on *Mir142*$^{-/-}$*BCR-ABL* LSK cells treated with SCR or miR-142-3p or/and miR-142-5p mimic (n=3 per group). **g-h.** mRNA (**g**; PDCD-1 and HAVCR2: n=8 for CP-T and n=7 for BC-T; CTLA-4: n=9 for CP-T and n=7 for BC-T) and protein (**h**; PD-1: n=7 per group; Tim-3: n=7 for CP CD4+ T, n=6 for CP CD8+ T, n=5 for BC CD4+ or CD8+ T; CTLA-4: n=8 for CP-T and n=7 for BC-T) levels of immune checkpoints (PDCD-1, HAVCR2 and CTLA-4) in human T cells from CP CML or BC CML patients. **i-j.** Representative plots of PD-1 and Tim-3 expression on CD4+ and CD8+ T cells (**i**) and combined results of PD-1 on CD4+ and CD8+ T cell subpopulations (**j**) from CP or BC CML patients (n=7 per group). Abbreviation: WT: wild type; KO: knock out; *B/A*: *BCR-ABL*; SCR: scramble RNA; CP: chronic phase; BC: blast crisis; CML: chronic myeloid leukemia; LSK: Lin-Sca-1+c-Kit+. For **a-d** and **f,** comparisons among multi-groups were performed by one-way ANOVA and P values were adjusted for multiple comparisons using Holm-Šídák method. For **e**, **g**, **h** and **j**, comparison between two groups was performed by two-tailed, unpaired t-test. Results shown represent mean ± SEM. Source data are provided as a Source Data file.

Supplementary Fig. 16

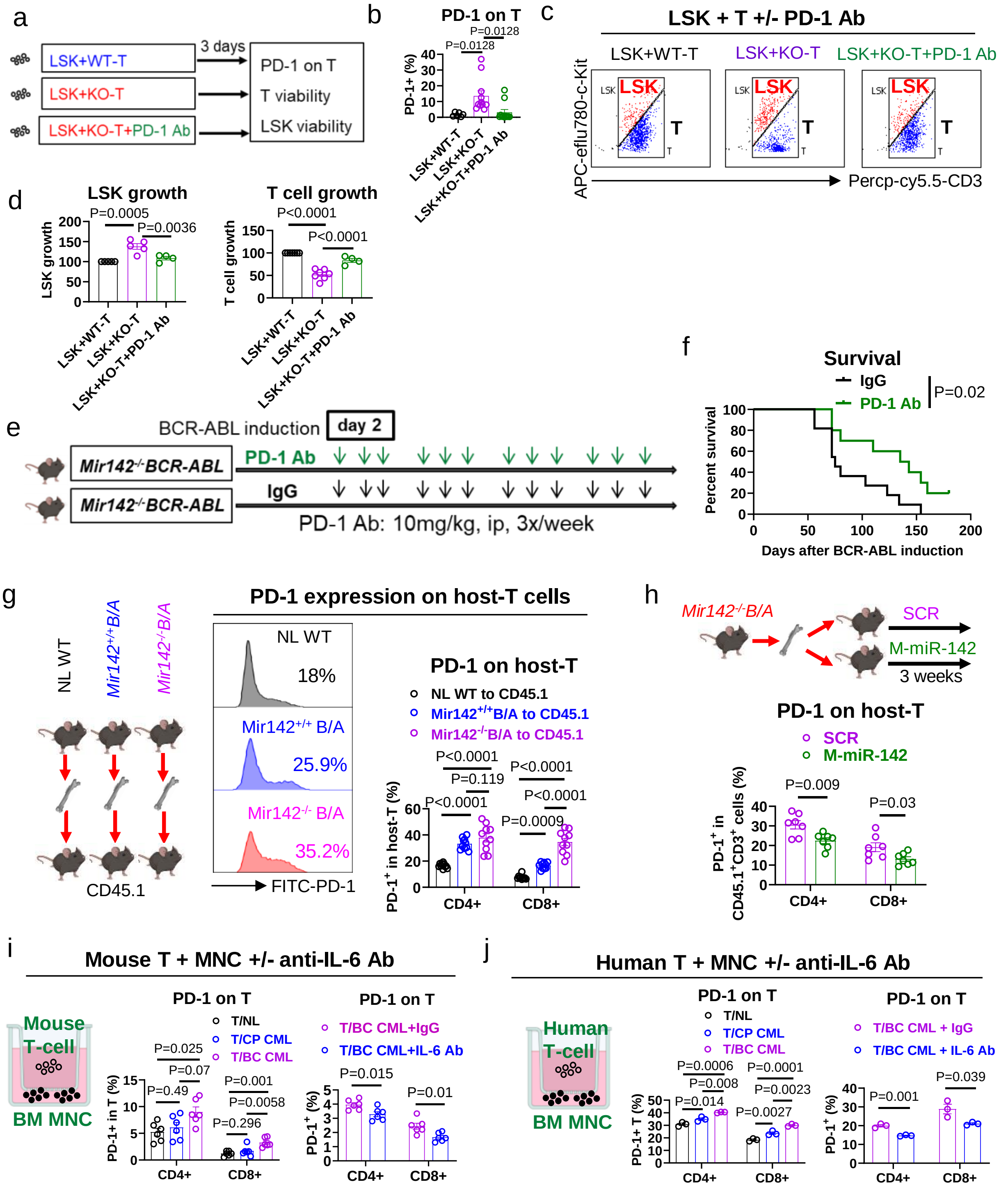

Supplementary Fig. 16: Blocking the upregulated PD-1 on T cells in BC CML can increase T cell antileukemic activity. **a-d.** Experimental design and results. *Mir142*$^{-/-}$*BCR-ABL* LSKs were co-cultured with *Mir142*$^{+/+}$ T (WT-T) or *Mir142*$^{-/-}$ T (KO-T) cells +/- PD-1 blocking Ab (1μg/ml) for 3 days (**a**). PD-1 expression on T cells (**b**; n=6 for WT-T group and n=11 for the other groups), representative plots (**c**) and combined results (**d**; LSK growth: n=5 for WT-T and KO-T groups and n=4 for KO-T+PD-1 Ab group; T cell growth: n=7 for WT-T and KO-T groups and n=4 for KO-T+PD-1 Ab group) of LSK and T cell numbers (relative to LSK+WT-T) were analyzed. **e-f.** Experimental design and results. *Mir142*$^{-/-}$*BCR-ABL* mice were treated with PD-1 blocking Ab or IgG control (10mg/kg, 3x/week) for 4 weeks (**e**) and survival is shown (**f**; n=10 per group; by Log-Rank test). **g.** PD-1 expression on host-T cells from the recipients of BM MNCs from wt, *Mir142*$^{+/+}$*BCR-ABL* or *Mir142*$^{-/-}$*BCR-ABL* mice (n=10 per group). **h.** PD-1 on host-T cells from the recipients of *Mir142*$^{-/-}$*BCR-ABL* BM MNCs, which were treated with SCR or M-miR-142 for 3 weeks (n=7). **i.** PD-1 on T cells cocultured with wt, *Mir142*$^{+/+}$*BCR-ABL* or *Mir142*$^{-/-}$*BCR-ABL* BM MNCs (**left**), or with *Mir142*$^{-/-}$*BCR-ABL* MNCs and treated with IgG or anti-IL-6 blocking Ab (**right**) for 3 days (n=6). **j.** PD-1 on human T cells cocultured with BM MNCs from healthy donors, CP, or BC CML patients (**left**), or with BC CML MNCs and treated with IgG or anti-IL-6 blocking Ab (**right**) for 3 days (n=3). Abbreviation: SCR: scramble RNA; CP: chronic phase; BC: blast crisis; MNC: mononuclear cells; wt: wild-type; Ab: antibody. For **b**, **d**, **g**, **i** and **j**, comparisons among multi-groups were performed by one-way ANOVA and P values were adjusted for multiple comparisons using Holm-Šídák method. For **h-j**, comparison between two groups was performed by two-tailed, unpaired t-test. Results shown represent mean ± SEM. For **e**, **g** and **h**, mouse images created in BioRender. Chen, F. (2025) https://BioRender.com/e61c469. For **i** and **j**, images created in BioRender. Chen, F. (2025) https://BioRender.com/i04w340. Source data are provided as a Source Data file.

Supplementary Fig. 17

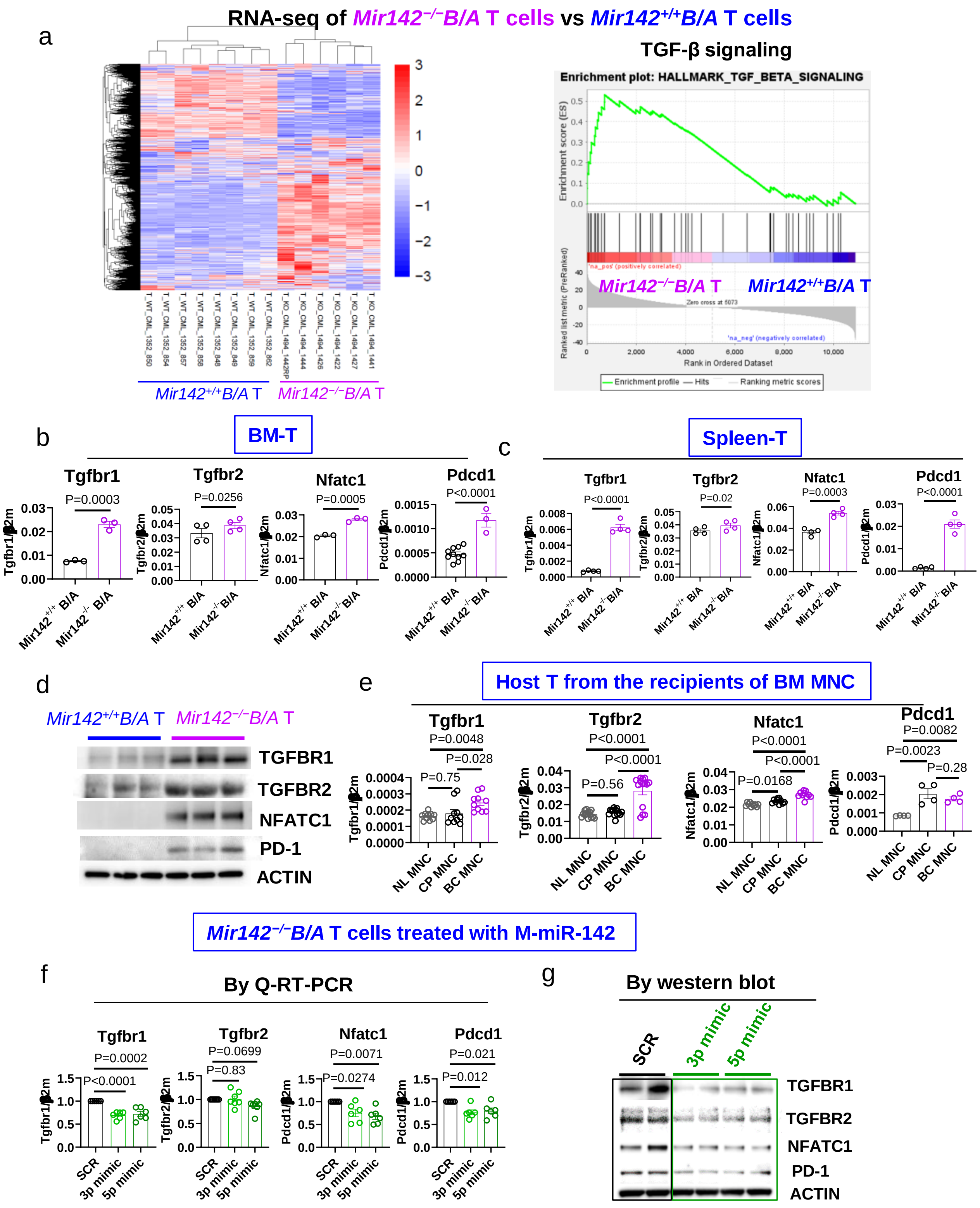

Supplementary Fig. 17: Increased PD-1 on T cells carrying miR-142 deficit was mediated by enhanced TGF-β signaling via upregulation of the miR-142 targets, TGFBR1 and TGFBR2. a. Heat map of differentially expressed genes in T cells from *Mir142*$^{-/-}$*BCR-ABL* versus *Mir142*$^{+/+}$*BCR-ABL* mice (BCR-ABL were induced by tet-off for 3 weeks) by RNA-seq analysis. Hallmark gene sets involved in TGF-β signaling are enriched in *Mir142*$^{-/-}$*BCR-ABL* T cells versus *Mir142*$^{+/+}$ *BCR-ABL* T cells. **b-d.** Tgfbr1, Tgfbr2, Nfatc1 and Pdcd1 mRNA levels in T cells from the BM (**b**) and spleen (**c**) by Q-RT-PCR (n=4 mice per group) and protein levels in spleen T cells by Western blotting (**d**; n=3 mice per group), selected from *Mir142*$^{-/-}$*BCR-ABL* versus *Mir142*$^{+/+}$ *BCR-ABL* mice (BCR-ABL were induced by tet-off for 3 weeks) are shown. **e.** BM MNCs from CD45.2 normal wt, *Mir142*$^{+/+}$*BCR-ABL* or *Mir142*$^{-/-}$*BCR-ABL* (BCR-ABL were induced by tet-off for 3 weeks) mice were transplanted into CD45.1 congenic normal wt mice, and mRNA expression levels of Tgfbr1, Tgfbr2, Nfatc1 and Pdcd1 in CD45.1+CD3+ host T cells were quantified by Q-RT-PCR (n=10 per group for Tgfbr1 and Nfatc1; n=14 per group for Tgfbr2; n=4 per group for Pdcd1). **f-g.** *Mir142*$^{-/-}$*BCR-ABL* T cells were treated with SCR, miR-142-3p or miR-142-5p mimic (2μM) for 3 days, then mRNA (**f**) and protein (**g**) levels of Tgfbr1, Tgfbr2, Nfatc1 and PD-1 were quantified by Q-RT-PCR (n=6 mice per group for Tgfbr1, Nfatc1 and Pdcd1; n=7 per group for Tgfbr2) and Western blotting (n=2 mice per group) respectively. For **d** and **g**: Results from one of the three independent experiments with similar results are shown. Abbreviation: *B/A*: *BCR-ABL*; SCR: scramble RNA; BM: bone marrow; MNC: mononuclear cells; tet: tetracycline; wt: wild-type. For **b** and **c**, comparison between two groups was performed by two-tailed, unpaired t-test. For **e** and **f**, comparisons among multi-groups were performed by one-way ANOVA and P values were adjusted for multiple comparisons using Holm-Šídák method. Results shown represent mean ± SEM. Source data are provided as a Source Data file.

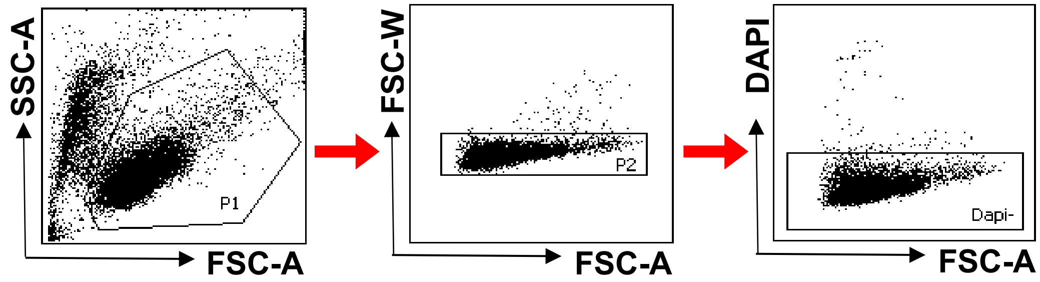

Supplementary Fig. 18: Gating strategy for flow cytometry analysis. All samples were FSC-A and SSC-A gated, followed by FSC-A/FSC-W gating to select singlet cells. DAPI negative cells were then gated for subsequent relevant gating, as shown in the main figures or supplementary figures.

Supplementary Table 1: Gene markers used to annotate the clusters of T cell scRNA-seq data

Cell type	Cluster	Gene markers	*Mir142$^{-/-}$B/A* vs *Mir142$^{+/+}$B/A*
CD4 naïve	0	CD4^{high}, CD8a^{neg}, CD44^{neg}, Sell (CD62L)^{high}, Ccr7^{high}, Lef1, Tcf7, Il7r	↓
CD8 naïve	1	CD4^{neg}, CD8a^{high}, CD44^{neg}, Sell (CD62L)^{high}, Ccr7^{high}, Il7r, Lef1, Tcf7, Nkg7, Ccr9, Dapl1, Plac8, Klf2	↓
CD4 naïve	2	CD4^{high}, CD8a^{neg}, CD44^{neg}, Sell (CD62L)^{high}, Ccr7^{high}, Lef1, Tcf7, Il7r	↓
CD8 central memory	3	CD8^{high}, CD44^{low}, Sell (CD62L)+, Il7r, Ccr7^{low}, Eomes, Prf1, Xcl1, Gzmb, Gzmm, Hopx, Tbx21(T-bet), Id2, Ifngr1, Nkg7, Cxcr6, Tmem176a, CD160, Bcl2, Cxcr3, Id2, Stat4, Ccl5, Plac8, Tox	↑
CD4 effector	4	CD4, Sell (CD62L)^{low}, Ccr7^{low}, CD44+, Il7r^{low}, Tcf7, Cxcr5, Pdcd1(PD-1), Ctla4, Lag3, Icos, Cd28, Tox^{high}, Ikzf2, Cxcr3, Cd40lg, Rora, Gata3, Slamf6, Tbc1d4, Btla, Il6st, Bcl6	↑
Regular T	5	CD4, Foxp3, Il2ra (CD25), Ikzf2, Ctla4, Tnfrsf9	↓
CD8 naïve	6	CD4^{neg}, CD8a^{high}, CD44^{neg}, Sell (CD62L)^{high}, Ccr7^{high}, Il7r, Lef1, Tcf7, Nkg7, Ccr9, Dapl1, Plac8, Klf2	↓
NKT	7	CD4^{low}, CD8^{low}, CD44, Il7r, Id2, Zbtb16 (PLZF), CD160, Il4, Ccl5, Cxcr3, Cxcr6, Gata3, Gzmk, Ifng, Gzma, Gzmb (Granzyme), Prf1 (Perfonin), Nkg7, Cxcr6, Ifngr1, Cd40lg, Rora, Prdm1(Blimp1), T-bet (Tbx21), Sell-, Ccr7-, Tcf7^{low}, Lef1^{low}	=
CD8 effector	8	CD4^{low}, CD8^{low}, Tnfrsf9, Tnf, Lta, Tgfb1, Bcl6, Stat3, Id3, Bcor	↑
CD8 central memory	9	CD4^{low}, CD8, Il7r, Sell, S1pr1, Klf2, Ifngr1	↑
CD4 effector	10	CD4, CD44, Id2, Cd244, Entpd1, Cd38, Lag3, Zeb2, Tgfb1	↑
Double negative T	11	Tcf7, Sell, Il7r, Nkg7, Tmem176a, Ccr9, Dntt, Ifngr1, Id3, Plac8, S1pr1, Klf2	↑
CD8 effector	12	CD4, CD8, Tmem176a, Btla, CD160, Mki67, Gzmb, Pdcd1, Ctla4, Icos, Tnfrsf9, Hopx, Tbx21, Tox, Tgfb1, Xcl1, Il12rb2, Gata3, Cxcr5, Eomes	↑
CD8 effector memory	13	CD8, Eomes, Tox, Stat4, Sell, Id3, Slamf6, CD28, CD27, Bcor, Ikzf2	=
CD4 effector	14	CD4, Tcf7^{low}, Sell^{low}, Lef1^{low}, Ccr7^{low}, Stat4^{low}	↑
Exhausted T	15	Plac8, Tmem176a, CD244, Entpd1, CD38, CD101, Mki67, Havcr2, Tnfrsf14, Hopx, Zeb2, Ifngr1, Tgfb1, Stat3, Anxa1, Ltb4r1, Bcor, Il6st	↑

Supplementary Table 2: Antibodies used for flow cytometry analysis

**Anti-mouse antibodies**				
Name	Conjugation/ Fluorochrome	Manufacturer	Clone	Cat#
CD3e	Biotin	eBioscience	17A2	13-0031-85
CD4	Biotin	eBioscience	GK1.5	13-0041-85
CD8a	Biotin	eBioscience	53-6.7	13-0083-85
B220	Biotin	eBioscience	RA3-6B2	13-0452-85
CD19	Biotin	eBioscience	eBio1D3 (1D3)	13-0193-85
IgM	Biotin	eBioscience	eB121-15F9	13-5790-85
Gr-1	Biotin	eBioscience	RB6-8C5	13-5931-85
CD11b	Biotin	eBioscience	M1/70	13-0112-85
NK1.1	Biotin	eBioscience	PK136	13-5941-85
Ter119	Biotin	eBioscience	TER-119	13-5921-85
Flt3	Biotin	eBioscience	A2F10	13-1351-85
Flt3	PE	eBioscience	A2F10	12-1351-82
Sca-1	PE-Cy7	eBioscience	D7	25-5981-82
CD117	APC-eFlu780	eBioscience	ACK2	47-1172-82
CD117	FITC	eBioscience	2B8	11-1171-82
CD117	APC-eFlu780	eBioscience	2B8	47-1171-82
CD150	PerCP-Cy5.5	Biolegend	TC15-12F12.2	115922
CD45.1	PE-Cy7	eBioscience	A20	25-0453-82
CD45.1	PerCP-Cy5.5	eBioscience	A20	45-0453-80
CD45.2	FITC	eBioscience	104	11-0454-85
CD45.2	eFluor450	eBioscience	104	48-0454-82
CD45	PE-Cy7	eBioscience	30-F11	25-0451-82
CD25	BV421	Biolegend	3C7	101923
CD44	PE	Biolegend	IM7	103007
CD62L	APC	Biolegend	MEL-14	104412
PD-1	FITC	eBioscience	RMP1-30	11-9981-82
CTLA-4	PE	eBioscience	UC10-4B9	12-1522-82
Tim-3	APC	eBioscience	8B.2C12	17-5871-82
IL-2	APC	eBioscience	JES6-5H4	17-7021-82
IFN-γ	PE	Biolegend	B27	506507
TNF-α	PerCP-Cy5.5	Biolegend	MAB11	502926

PD-L1	PE-Cy7	eBioscience	MIH5	25-5982-80
CD25	BV-421	eBioscience	PC61.5	404-0251-82
CD69	Percp-Cy5.5	eBioscience	H1.2F3	45-0691-82
CD127	BV421	Biolegend	A7R34	135027
CD11b	FITC	eBioscience	M1/70	11-0112-82
CD11b	PE	eBioscience	M1/70	12-0112-83
CD19	APC	eBioscience	eBio1D3	17-0193-82
CD3	Percp-eFlu710	eBioscience	17A2	46-0032-82
CD4	FITC	Biolegend	RM4-5	100510
CD8	PE-Cy7	Biolegend	53-6.7	100722
TCRβ	PE	Biolegend	H57-597	109208
TCRγδ	BV421	Biolegend	GL3	118120
**Anti-human antibodies**				
Name	Conjugation /Fluorochrome	Manufacturer	Clone	Cat#
CD34	FITC	eBioscience	4H11	11-0349-42
CD34	PE-Cy7	eBioscience	4H11	25-0349-42
CD34	APC	eBioscience	4H11	17-0349-42
CD38	PE	eBioscience	HIT2	12-0389-42
CD38	FITC	eBioscience	HB7	11-0388-42
CD45	APC	BD Pharmingen	HI30	555485
CD45	FITC	BD Pharmingen	HI30	561865
CD33	PE	BD Pharmingen	WM53	555450
PD-1	PE	Biolegend	EH12.2H7	329906
PD-1	APC-Cy7	Biolegend	EH12.2H7	329922
PD-L1	PE-Cy7	Biolegend	29E.2A.3	329718
CTLA-4	APC	Biolegend	BNI3	369612
Tim-3	PE	Biolegend	A18087E	364806
CD4	FITC	Biolegend	A161A1	357406
CD8	PE-Cy7	BD Pharmingen	RPA-T8	557746
CD69	FITC	BD Pharmingen	FN50	555530
CD25	APC-Cy7	BD Pharmingen	M-A251	557753
CD2	Percp-Cy5.5	Biolegend	RPA-2.10	300216
CD3	BV510	Biolegend	OKT3	317332
CD90	PE	eBioscience	eBio5E10 (5E10)	12-0909-42

CD45RA	BV605	BD	HI100	562886
CCR7	PE-Cy7	eBioscience	3D12	25-1979-42
**Other antibodies**				
Annexin V	PE	BD Pharmingen		559763
Annexin V	APC	BD Pharmingen		550475
Annexin V	FITC	BD Pharmingen		560931
Ki-67	Alexa Fluor 647	BD Pharmingen	B56	561126
Streptavidin	PE	eBioscience		12-4317-87
Streptavidin	FITC	eBioscience		11-4317-87
Streptavidin	APC	eBioscience		17-4317-82

Supplementary Table 3: TaqMan Gene Expression Assays

**Gene name**	**Assay ID**	**Manufacturer**
snoRNA234	1234	ThermoFisher
RNU44	1094	ThermoFisher
miR-142-3p	464	ThermoFisher
miR-142-5p	2248	ThermoFisher
IL-6	Hs00174131_m1	ThermoFisher
β2m	Mm00437762_m1	ThermoFisher
β2M	Hs00187842_m1	ThermoFisher
Pdcd-1	Mm01285676_m1	ThermoFisher
PDCD-1	Hs01550088_m1	ThermoFisher
HAVCR2 (TIM3)	Hs00958618_m1	ThermoFisher
CTLA-4	Hs00175480_m1	ThermoFisher
Tgfbr1	Mm00436964_m1	ThermoFisher
Tgfbr2	Mm03024091_m1	ThermoFisher
Nfatc1	Mm01265944_m1	ThermoFisher

Supplementary Table 4: Cytokines and chemokines measured by Luminex assay (R&D systems)

Analytes:	Species
CXCL12/SDF-1 alpha (BR54)	mouse
G-CSF (BR39)	mouse
GM-CSF (BR12)	mouse
IFN-gamma (BR33)	mouse
IL-1 alpha/IL-1F1 (BR47)	mouse
IL-1 beta/IL-1F2 (BR19)	mouse
IL-2 (BR22)	mouse
IL-3 (BR34)	mouse
IL-4 (BR25)	mouse
IL-5 (BR26)	mouse
IL-6 (BR27)	mouse
IL-10 (BR28)	mouse
IL-12 p70 (BR15)	mouse
IL-13 (BR29)	mouse
IL-16 (BR35)	mouse
IL-17/IL-17A (BR30)	mouse
IL-17E/IL-25 (BR55)	mouse
IL-27 (BR56)	mouse
IL-33 (BR43)	mouse
TNF-alpha (BR14)	mouse
VEGF (BR21)	mouse
