# Supplementary material for: miR-142 deficit in T cells during blast crisis promotes chronic myeloid leukemia immune escape
Source: Nat Commun. 2025 Feb 1;16:1253. doi: 10.1038/s41467-025-56383-y (PMC11787332; doi:10.1038/s41467-025-56383-y)
Supplement: Supplementary file 2 — Description of Additional Supplementary Files [file 41467_2025_56383_MOESM2_ESM.pdf]

Description of Additional Supplementary Files

Supplementary Data 1: Normalized area of differentially abundant non-redundant metabolites (p-value<0.05) in resting and 24 hrs-activated T cells from *Mir142*$^{-/-}$*BCR-ABL* (KO-T) and *Mir142*$^{+/+}$*BCR-ABL* (WT-T) mice.

Supplementary Data 2: Area and differential analysis of glycolytic and OxPhos metabolites of resting and 24 hrs-activated T cells from *Mir142*$^{-/-}$*BCR-ABL* (KO-T) and *Mir142*$^{+/+}$*BCR-ABL* (WT-T) mice.

Supplementary Data 3: Ratios and differential analysis of glycolytic and OxPhos metabolites of resting and 24 hrs-activated T cells from *Mir142*$^{-/-}$*BCR-ABL* (KO-T) and *Mir142*$^{+/+}$*BCR-ABL* (WT-T) mice.

Supplementary Data 4: Normalized areas of all compounds detected in the resting and 24 hrs-activated T cells from *Mir142*$^{-/-}$*BCR-ABL* (KO-T) and *Mir142*$^{+/+}$*BCR-ABL* (WT-T) mice.
