# Supplementary material for: miR-142 deficit in T cells during blast crisis promotes chronic myeloid leukemia immune escape
Source: Nat Commun. 2025 Feb 1;16:1253. doi: 10.1038/s41467-025-56383-y (PMC11787332; doi:10.1038/s41467-025-56383-y)
Supplement: Supplementary file 7 — Reporting Summary [file 41467_2025_56383_MOESM7_ESM.pdf]

nature portfolio

Corresponding author(s): Bin Zhang, Guido Marcucci

Last updated by author(s): 12/15/2024

Reporting Summary

Nature Portfolio wishes to improve the reproducibility of the work that we publish. This form provides structure for consistency and transparency in reporting. For further information on Nature Portfolio policies, see our Editorial Policies and the Editorial Policy Checklist.

Statistics

For all statistical analyses, confirm that the following items are present in the figure legend, table legend, main text, or Methods section.

n/a	Confirmed	
☐	☒	The exact sample size (*n*) for each experimental group/condition, given as a discrete number and unit of measurement
☐	☒	A statement on whether measurements were taken from distinct samples or whether the same sample was measured repeatedly
☐	☒	The statistical test(s) used AND whether they are one- or two-sided<br>*Only common tests should be described solely by name; describe more complex techniques in the Methods section.*
☒	☐	A description of all covariates tested
☐	☒	A description of any assumptions or corrections, such as tests of normality and adjustment for multiple comparisons
☐	☒	A full description of the statistical parameters including central tendency (e.g. means) or other basic estimates (e.g. regression coefficient) AND variation (e.g. standard deviation) or associated estimates of uncertainty (e.g. confidence intervals)
☐	☒	For null hypothesis testing, the test statistic (e.g. *F*, *t*, *r*) with confidence intervals, effect sizes, degrees of freedom and *P* value noted<br>*Give P values as exact values whenever suitable.*
☒	☐	For Bayesian analysis, information on the choice of priors and Markov chain Monte Carlo settings
☒	☐	For hierarchical and complex designs, identification of the appropriate level for tests and full reporting of outcomes
☒	☐	Estimates of effect sizes (e.g. Cohen's *d*, Pearson's *r*), indicating how they were calculated

Our web collection on statistics for biologists contains articles on many of the points above.

Software and code

Policy information about availability of computer code

Data collection	BD FACSDiva (v9.0) was used to collect flow cytometry data. Xcalibur 4.4.16.14 was used to collect data for untargeted metabolomics.
Data analysis	Graphpad Prism v10 (for in vitro and in vivo data analysis); BD FACSDiva v9.0 and FlowJo v10.8.1 (flow cytometry data); Cell Ranger v6.1.2, Seurat v4.1.0 and clusterProfiler v4.2.2 (scRNA sequencing); STAR v.2.6.0.a, HTSeq-count v.0 .11.1 and "edgeR" (RNA sequencing); Compound discoverer 3.2, Skyline 20.1.0.155, SMPD and KEGG metabolic pathways (untargeted metabolomics).

For manuscripts utilizing custom algorithms or software that are central to the research but not yet described in published literature, software must be made available to editors and reviewers. We strongly encourage code deposition in a community repository (e.g. GitHub). See the Nature Portfolio guidelines for submitting code & software for further information.

Data

Policy information about availability of data

All manuscripts must include a data availability statement. This statement should provide the following information, where applicable:

- Accession codes, unique identifiers, or web links for publicly available datasets
- A description of any restrictions on data availability
- For clinical datasets or third party data, please ensure that the statement adheres to our policy

RNA sequencing and scRNA sequencing data are available at GeneExpression Omnibus (GEO; RNA-seq: GSE261798; scRNA-seq: GSE254285 for mouse cells and GSE276130 for human cells) repository of the National Center for Biotechnology Information. Metabolomic profiles are available at the NIH Common Fund's National Metabolomics Data Repository (NMDR) website [http://dev.metabolomicsworkbench.org:22222/data/DRCCMetadata.php?

Mode=Study&StudyID=ST003091&Access=HieS7992, The DOI for this project (PR001920) is: http://dx.doi.org/10.21228/M8JB09].

Research involving human participants, their data, or biological material

Policy information about studies with human participants or human data. See also policy information about sex, gender (identity/presentation), and sexual orientation and race, ethnicity and racism.

Reporting on sex and gender	This information has not been collected.
Reporting on race, ethnicity, or other socially relevant groupings	This information has not been collected.
Population characteristics	Human blood or BM donors were between 30-70 years old. CML patients were diagnosed with either CP CML or BC CML, with BCR-ABL+ by FISH or PCR analysis. The samples were collected from the patients before the treatment.
Recruitment	Human blood or BM donors for experiments were anonymous.
Ethics oversight	Sample acquisition was approved by the Institutional Review Board (IRB) at the COHN MC, in accordance with an assurance filed with and approved by the Department of Health and Human Services and met all requirements of the Declaration of Helsinki. Patients with CML were consented on the IRB # 18067 protocol.

Note that full information on the approval of the study protocol must also be provided in the manuscript.

Field-specific reporting

Please select the one below that is the best fit for your research. If you are not sure, read the appropriate sections before making your selection.

- [x] Life sciences
- [ ] Behavioural & social sciences
- [ ] Ecological, evolutionary & environmental sciences

For a reference copy of the document with all sections, see nature.com/documents/nr-reporting-summary-flat.pdf

Life sciences study design

All studies must disclose on these points even when the disclosure is negative.

Sample size	Sample sizes were not based on formal power calculations. It was based on the magnitude, sample availability, consistency of measurable differences between groups. We repeated all of the experiments in the paper using at least 3-4 samples from mouse or human individuals to ensure adequate power. For animal experiments, animal numbers were chosen based on experimental group size, mice availability and variability, treatment frequency and previous experience.
Data exclusions	No animals or samples were excluded from analysis.
Replication	Experiments were replicated multiple times with reproducible results indicated in the figure legends.
Randomization	For all animal experiments, the mice with the same gender and age were divided randomly into experimental groups to reduce variation and enhance power.
Blinding	For some animal studies, the investigators were blinded to the mice allocations while performing the treatment or monitoring the mice for survival, and the investigators learned the genotypes when analyzing the results. For the remaining experiments, the Investigators could not be blinded to sample allocations because they have to allocate these samples first before the treatments.

Reporting for specific materials, systems and methods

We require information from authors about some types of materials, experimental systems and methods used in many studies. Here, indicate whether each material, system or method listed is relevant to your study. If you are not sure if a list item applies to your research, read the appropriate section before selecting a response.

Materials & experimental systems

n/a	Involved in the study
	☒ Antibodies
	☒ Eukaryotic cell lines
☒	☐ Palaeontology and archaeology
	☒ Animals and other organisms
☒	☐ Clinical data
☒	☐ Dual use research of concern
☒	☐ Plants

Methods

n/a	Involved in the study
☒	☐ ChIP-seq
	☒ Flow cytometry
☒	☐ MRI-based neuroimaging

Antibodies

Antibodies used	Anti-mouse antibodies: lineage markers-biotin (Ter-119, CD3, NK1.1, lgM, CD4 , CD8a, CD19 , Gr-1 , CD11b, B220), CD45-PE or FITC or APC-eflu780, CD45.l-PE-Cy7 or PE, CD45.2-FITC or APC or pacific blue, Flt3-biotin or PE, IL-7R -biotin or PE-Cy7, CD117 (c-Kit, clone: ACK2)-APC-eflu780 or FITC, Sca-1-PE or PE-Cy7, PD-1-FITC, CTLA-4-PE, Tim-3-APC, IL-2-APC, PD-L1-PE-Cy7, CD25-BV421, CD69-Percp-cy5.5, CD11b-PE or FITC, CD19-APC, CD3-Percp-eflu710 or APC-eflu780 (all from eBioscience, San Diego, CA), CD25-BV421, CD44-PE, IFN-gamma-PE, TNF-alpha-Percp-cy5.5, CD127-BV421, CD4-FITC, CD8-PE-cy7, TCR-betta-PE, TCR-gamma/delta-BV421 (Biolegend, San Diego, CA). Anti-human antibodies: CD34-PE-Cy7 or FITC or APC, CD38-PE or APC or APC-eflu780, CD90-PE, CCR7-PE-Cy7 (all from eBioscience), CD45-APC or FITC, CD33-PE, CD45RA-BV605 (all from BD), PD-1-PE or APC-cy7, PD-L1-PE-Cy7, CTLA-4-APC, TIM3-PE, CD4-FITC, CD3-BV510, CD2-Percp-cy5.5 (all from Biolegend, San Diego, CA), CD8-PE-cy7, CD25-APC-cy7, CD69-FITC (all from BD). Other antibodies: anti-streptavidin-APC or PE or FITC, Ki-67-APC or Alexflu 647, Annexin V-PE or FITC or APC. Detailed antibody information including manufacturer, cat number and/or clone number were provided in Supplementary Table 2.<br>Antibodies for immunoblotting: TGFBR1 (ab235578, Abcam), TGFBR2 (ab269279, Abcam), PD-1 (AF1021, R&D), ACTIN (8H10D10, Cell Signaling), NFAT2 (#8032, Cell Signaling).
Validation	Validation of all primary antibodies for the species and application was performed using variable strategies: cellular distribution, size of bands in western blotting experiments. Antibodies critical for novel conclusions were validated by elimination of signals upon knocking down or inhibiting experiments and/or by functional assays. Validation statements of all antibodies used in this manuscript were noted on the manufacturer's website with relevant citations.

Eukaryotic cell lines

Policy information about cell lines and Sex and Gender in Research

Cell line source(s)	OP9-DL1 cell line is obtained from the Riken Bio-Resource Center (BRC, Tsukuba, Japan)
Authentication	OP9-DL1 cell line was obtained from the company and used for this study right away, so it was not authenticated
Mycoplasma contamination	Cell line tested negative for mycoplasma contamination
Commonly misidentified lines (See ICLAC register)	No commonly misidentified cell lines were used in this study

Animals and other research organisms

Policy information about studies involving animals; ARRIVE guidelines recommended for reporting animal research, and Sex and Gender in Research

Laboratory animals	6-10 weeks old wild-type (WT), Mir142 KO, SCLtTA/BCR-ABL (BCR-ABL), Mir142 KO/BCR-ABL, Mir142flox/flox/Lck-cre+ (all CD45.2 C57BL B6, from collaborator or homemade), CD45.1 C57BL B6 (as recipient mice, from Charles River), NSG and NSGS-SGM3 (both from Jax lab) mice were used.
Wild animals	No wild animals were used in this study.
Reporting on sex	Both male and female mice were used.
Field-collected samples	No field-collected samples were used.
Ethics oversight	Mouse care and experimental procedures were performed in accordance with federal guidelines and protocols were approved by the Institutional Animal Care and Use Committee at City of Hope (IACUC #15005). All experimental mice are maintained on 12:12-h light:dark cycle, 68-79 F temperature and 30-70% humidity.

Note that full information on the approval of the study protocol must also be provided in the manuscript.

Plants

Seed stocks: n/a

Novel plant genotypes: n/a

Authentication: n/a

Flow Cytometry

Plots

Confirm that:

- [x] The axis labels state the marker and fluorochrome used (e.g. CD4-FITC).
- [x] The axis scales are clearly visible. Include numbers along axes only for bottom left plot of group (a 'group' is an analysis of identical markers).
- [x] All plots are contour plots with outliers or pseudocolor plots.
- [x] A numerical value for number of cells or percentage (with statistics) is provided.

Methodology

Sample preparation: Human and mouse cells were collected and stained with antibodies for 30 minutes at 4C, as detailed in the methods.

Instrument: BD Fortessa x20, BD Aria Fusion

Software: BD FACSDiva v9.0 and FlowJo v10.8.1

Cell population abundance: At least 10,000 cells were collected for analysis. Numbers of cells collected by sorting were listed in the methods or figure legends. Purity was determined by running a purity check of the sorted populations after the sort was completed.

Gating strategy: All samples were initially gated using forward scatter and side scatter to identify events corresponding to cells, next using forward scatter height vs. area to enrich for single cells, and then alive cells were selected by gating on negative cells for viability dye.

- [x] Tick this box to confirm that a figure exemplifying the gating strategy is provided in the Supplementary Information.
