# Supplementary material for: miR-142 deficit in T cells during blast crisis promotes chronic myeloid leukemia immune escape
Source: Nat Commun. 2025 Feb 1;16:1253. doi: 10.1038/s41467-025-56383-y (PMC11787332; doi:10.1038/s41467-025-56383-y)
Supplement: Supplementary file 8 — Transparent Peer Review file [file 41467_2025_56383_MOESM8_ESM.pdf]

nature portfolio

Peer Review File

miR-142 deficit in T cells during blast crisis promotes chronic myeloid leukemia immune escape

Corresponding Author: Dr Bin Zhang

This file contains all reviewer reports in order by version, followed by all author rebuttals in order by version.

Version 0:

Reviewer comments:

Reviewer #1

(Remarks to the Author)
In this manuscript by Chen et al. the authors build on previous work which describes lack of miR-142 to be associated with blast crisis (BC) chronic myeloid leukemia (CML). The authors demonstrate in the current manuscript that miR-142 is also decreased in T cells of BC CML patients and the employed murine model of BC CML. They implicate interleukin (IL)-6, secreted by the BC cells, to be the mediator of this phenomenon. The authors go on to show that miR-deficiency impairs T cell differentiation and function, in particular antileukemic activity. These T cells also showed increased exhaustion, which was found to be due to decreased metabolic reprogramming in T cells, and apoptosis. A miR-142 mimetic rescued the decreased T cell function and enhanced T cell number and antileukemic function. This led to survival prolongation in the BC CML model and xenotransplantation models.
The manuscript is well written, comprehensive, and it includes several murine models and elegant experiments, all of which add to a careful description of this newly discovered and important pathway. A couple of questions remain, and, overall, the mechanism on the involvement of the IL-6 pathway should be strengthened.
Major points:
1. Suppl. Fig 3b,c: Why are gamma delta T cells more affected than alpha beta T cells?
2. A quantification of all flow plots (for several mice) is needed.
3. Please describe the nature of the miR compound.
4. Figure 6i: More human samples are needed for the PDX models. One sample is not sufficient.
5. Please describe the GvHD in the mice treated with T+M-miR-142. Why did the T cells in these mice transplanted with autologous human T cells expand uncontrollably?
6. It would be informative to stratify human BC CML patients by expression of miR-142 and plot survival data.
7. Figure 2b and others: Why do the authors use B cell markers and negativity for CD19 rather than myeloid markers? Or why not use CD4 instead of CD25?
8. Figure 2d: How do you explain the increase in myeloid cells mechanistically in Mir-142 -/- mice?
9. Is the function of Treg cells affected by IL-6, or deficiency of MiR-142?
10. Figure 6d: It seems as if the blasts have differentiated? How and why?
11. Figure 7c: A better image is necessary for the vehicle group, as the presented cells do not look like blasts, but more like mature neutrophils.
12. Figure 7e: Did the combination treatment really keep all but one mouse alive? What did the bone marrow look like in the mice which survived, i.e. what cell types survived the treatment to cause death in 2 mice in the secondary transplantation experiment?

Minor points:
Suppl. Fig. 3C: Please fix the legend on the y-axis.

Reviewer #2

(Remarks to the Author)
This manuscript by Chen and Zhao et al. describes the role of miR-142 in T cell-mediated control of CML progression to a

blast crisis stage. miR-142 deficit was associated with loss of downstream T cell expansion by programming progenitors towards a myeloid lineage. This manuscript is clear with logical, well thought out mechanistic experiments to address hypotheses. The data supporting the role of miR-142 in T cell persistence/expansion and apoptosis is strong and well supported. The data in Figure 7 are also really nice. However, the data relating to the role of miR-142 in T cell exhaustion and differentiation require additional evidence to support the current conclusions or the conclusions should be softened.

Specific comments:

- Throughout the results section, the authors make comments that slightly overinterpret the data or are partially unsupported (line 329 for example). It detracts from the strength of the mechanistic experiments and the manuscript would be greatly enhanced by checking that supportive evidence is presented and subsequently editing these sentences as necessary.

- The authors focus on changes in T cell differentiation in figure 3. The overall reduction of T cell numbers makes the changes in frequency hard to interpret. Providing absolute counts for the flow cytometry data will be much clearer and more interpretable.

- Figure 3 human data: How were the human cells stimulated to measure cytokines? In (D) Was the phenotype of the control vs KD T cells similar prior to stimulation? Ie. did they have the same proportion of effector cells? In (E) was production of IFNg or TNF impacted?

- Figure 4 clearly demonstrates that Mir142 KO T cells do not engraft/expand in secondary recipients and it is evident that miR-142 is crucial for persistence of antileukemic T cells. However, the conclusion that these T cells have impaired function/killing activity is not supported.

- Throughout the manuscript T cell numbers need to be enumerated and presented as total counts not frequencies. Frequencies in blood will always be lower in untreated mice due to high numbers of circulating blasts. If total numbers are not possible, % within CD45.1+ cells to exclude blasts could be presented instead.

- Figure 6: T cell depletion experiments need to be performed in the immunocompetent model to prove that the effect of the M-miR-142 is T cell mediated. The therapy is interesting regardless of whether it is acting directly on T cells or by directly inhibiting leukemia cell growth. However, if the authors wish to conclude that the mechanism is T cell-dependent, this needs to be definitively demonstrated. The PDX model in (J) suggests that there are T cell independent mechanisms and this needs to be discussed.

- There is no clear evidence to support the conclusions related to T cell exhaustion and hypofunctionality. If the authors wish to focus on this, supporting evidence should be moved into main figures and sustainably strengthen with in vivo killing assays using purified TEFF populations with and without an miR-142 deficit.

Minor concerns:
- The colors for the miR-142 non-tumor controls in Figure 1 cannot both be black.
- Line 188, how were cells stimulated to measure cytokines? This should ideally be included in the text.
- Line 329. Is there data to support the conclusion that the IL-6 is being produced by blasts? If not, the "blast-produced" part of this sentence could be removed.
- Line 334: PD-1 is an activation marker and not an exhaustion marker.
- Lines 352 - 356: it should be made clearer that there is an association between reduced miR-142 levels and increased expression of inhibitory T cells as the evidence does not support causation here.
- The terminology for TKI is different between the text (TKI) and figure (NIL) in Figure 7.
- "Engraftment" terminology is confusing.

Reviewer #3

(Remarks to the Author)
This manuscript follows up a previous paper by the authors that reported that the loss of miR-142 results in the chronic to blast crisis (BC) transition of CML. This manuscript investigates the immune escape mechanisms of CML in blast crisis. The authors found that miR-142 levels were reduced ~40% in T cells from CML BC patients compared to chronic phase patients or healthy individuals. This finding led to a series of studies, with many experiments, that led the authors to conclude that the reduction in miR-142, which affects T cell development and probably also T cell function, reduces a T-cell-based anti-leukemic immune response.

miR-142 has been known to control multiple lineages in the hematopoietic system, including a major reduction in T and B cells reported previously in the miR-142 KO mice. The function of miR-142 in driving CML BC transition has been reported by the same lab previously. The effects of anti-PD-L1/PD-1 on CML mouse model have been reported by multiple studies. Compared to the existing knowledge, the main novelty of the current manuscript includes (a) the proposal that the loss of miR-142 in T cells is an important driver in CML progression, and (b) there is a significant T-cell-based anti-CML immunity that is miR-142 dependent. If both are solidly proven, the manuscript could be interesting to the readership of Nature Communications. However, there are major concerns related to both and other aspects.

Major Concerns:

1. The authors showed in sup fig 1a that the reduction of miR-142 in patients is only about 40%. This raises major questions regarding the meaning and significance of using miR-142 homozygous KO to model the human condition. The authors could examine the effect of miR-142 heterozygous loss, especially in T cells, in controlling CML progression.
2. The genetic backgrounds of the mice are critical in interpreting the T-cell-based anti-leukemic effects. Are these due to immunity against leukemic cells or against the genetic background the leukemic cells are on, especially if there are any mixed background effects? For BCR-ABL model and miR-142 KO or fl/fl, the method says they are on the B6 background. Which B6 strain (J, N, or others?) and the history of creation and backcrossing?
3. There is no direct evidence for a T cell-response toward leukemia. Many of the data presented can be interpreted equally as an indirect effect (which can be many possibilities). It will be very helpful to examine whether T cell activation status is affected by the presence of BC-CML cells directly, such as via in vitro co-culture experiments.
4. Does miR-142 affect the output of NK, B, DC cells from LMPP?
5. For miR-142 inhibitor, can the authors clarify why both 5p and 3p inhibitors work? Does the 5p inhibitor affect the 3p miR-142? And does 3p inhibitor affect 5p level?
6. There are many descriptions of the changes in miR-142 null T cells or T cell differentiation, but the mechanisms are not carefully addressed. Is TGF-b pathway the key reason? The authors need to at least discuss this aspect. Introduction of known functional targets in immune cells could aid readers in gaining a more comprehensive understanding.

Minor Concerns:
1. The manuscript has a large amount of data, some of which are not obviously meaningful. The authors can consider reducing those areas. For example, much of the scRNAseq and metabolomic studies is descriptive, neither proves or disproves a key point, and is challenging for readers to read through. For scRNAseq, the assignment of clusters is questionable.
2. For scRNAseq, there are supposed to be three mice for each condition. The authors should show (if they want to continue to include those sections) the statistics with mean and std, e.g. for cluster size.
3. Fig 5c: the font for IFN-g and TNF-a is problematic?

Reviewer #4

(Remarks to the Author)
In this manuscript authors have studied the impact of miRNA 142 on T cell immunity in CML. They suggest that miRNA is downregulated in T cells in blast phase CML patients and that leads to impaired T cell mediated anti-leukemia immunity. They perform careful in vivo mouse studies with various models and propose that acquired miRNA deficit in T cells during blast transformation is due cytokine secretion (such as IL6) and that leads to impaired function and exhaustion. In addition, in mouse models their results show that replacement of miRNA142 improves survival. Overall, studies are carefully done utilizing a lot of various mouse models, but it is still a bit difficult to understand the role of miRNA142 as that does not seem to have effect alone on immune cell development and changes are only observed in the blast phase model.

More specific comments are below

1) Key starting point in results is that blast phase CML patients have lower levels of miRNA142 than controls or CP patients and this is based on the supplementary figure 1A. However, this figure does not prove that this is the case, as it could also be that levels of miRNA142 can be similar in specific T cell types and just some T cell subtype is decreased in BP CML patients. Authors should use other metrics to show that expression levels of miRNA are really decreased in BP-CML patients (single cell analysis?) or that there is no difference in their T cell subtypes compared to controls or CP patients

2) In the next sentence authors claim that "Accordingly, using genetically engineered mouse models (GEMMs), we also observed that the BC Mir142–/–BCR-ABL mouse presented with significant T lymphopenia…" But this is quite different what is observed in patients as they do not have T lymphopenia? Or do authors have some evidence that there is T cell lymphopenia in patients? Furthermore, the T cell data in mouse models in suppl fig 1 is shown as percentage of all cells and is there real difference among absolute numbers? Ie is it just that the total WBC count is increased and there are more other cell types?

3) In Figure 1C single cell data is shown from various mouse models which suggest that miRNA deficit alone does not change stem cell compartment but actually the formation of blast crisis changes it. Some comparison to patient single cell data would be needed here (data available from previous published studies)

4) Did authors also study NK cell development? If LMPP was myeloid biased, which cells were produced then?

5) In vivo anti-leukemic activity. If understood correctly, miRNA142 KO LSK cells were co-transplanted with Mir142+/+ T or Mir142-/- T cells (but not BCR-ABL pos)? How would authors interpret these results as their earlier results mostly showed that the T cell impairment was mostly seen in miRNA142-/- BCR-ABL mice LMPPs and not with WT miRNA142-/-?

6) Deficit of miRNA142 and PD1 expression. As authors show earlier that miRNA deficit is causing reduction of naïve cells could that be reason for increased PD1 expression as naïve cell are known to express lower levels of PD1? Ie no direct effect on PD1, but change in the subpopulations of T cells? Was that observed spontaneous apoptosis more pronounced in naïve cells? Similarly in patient samples, PD1 and other checkpoint expression should not only be shown in T cells, but in subpopulations as it has already been shown before even in CML that different T cell subpopulations have different

expression levels of checkpoint receptors

7) The discussion should take into account points above and also critical discussion how well this presents situation in blast phase patients. In addition, it is well known that blast phase can occur during multiple mechanisms and for example acquisition of new driver genes which could also have impact on immune cell function and immune evasion

Reviewer #5

(Remarks to the Author)
I co-reviewed this manuscript with one of the reviewers who provided the listed reports. This is part of the Nature Communications initiative to facilitate training in peer review and to provide appropriate recognition for Early Career Researchers who co-review manuscripts.

Version 1:

Reviewer comments:

Reviewer #1

(Remarks to the Author)
In this revised manuscript the authors have addressed this reviewer's concerns. They can be applauded for this work.
The only questions that remains to this reviewer is whether there is reduced 'homing' of KO T cells (Figure 4b), which could explain this finding.

Reviewer #2

(Remarks to the Author)
Major concerns:
• It appears that all the stats are t-tests according to the figure legends? Were corrections for multiple comparisons performed? It is not appropriate to present multiple groups in the same plot without correcting for multiple comparisons. The data most likely to be affected by this are the quantification of WBC, leukemic cells and T cells in Fig.7B, D, F, I and J.

• Fig1D, what is the expression level of key genes in each subset? A dot plot depicting gene expression and percent expressed was generated for the human dataset and it would be helpful if this was shown for the mouse sequencing as well.

• In Fig.3A a stacked bar graph (or something similar) would be helpful to visualize changes in T cell subset distribution. The UMAPS do not obviously depict the observed changes in frequency.

• In Fig.3B the T cell viability is reduced. As effector to target ratios decrease, the killing of tumor cells decreases. Killing assays usually show a curve with several E:T ratios and demonstrate reduced killing at lower ratios regardless of whether the effector T cells can capably kill tumor cells at higher ratios. This is not a readout of loss of T cell function and is rather related to the physical ratio of the T cells to the target. The changes in viability/apoptosis/cell growth in both the mouse and human KO T cells are much larger than the small decreases in cytokine production (many of which are not statistically significant) throughout the manuscript. These data do not demonstrate that Mir142 KO T cells have reduced killing capacity (are there changes in cytolytic proteins such as granzyme, CD107a, perforin?). These data rather support the conclusion that Mir142 KO cells have decreased survival/cell cycle arrest and thus have reduced anti-leukemic activity due to reduced numbers of T cells. This is an important distinction when describing the mechanism. Persistence of T cells is crucial for responses to immunotherapy and this mechanism likely contributes to the improved survival seen in Figs. 6-7.

• How are the T cell subsets quantified in Fig.S7B? The distribution of phenotypes within CD4 and CD8 T cells (the flow plots) or total cell numbers of each population as is shown in Fig.S1F would be the most informative way to quantify this. The increase in memory CD8 T cells is the most striking finding in S1F and S7B and this is not mentioned in the manuscript?

• Human data in Supp Fig. 5:
o There is no significant reduction in T-primed subsets, both statistically and in magnitude.
o What is the rationale for naming C3 and C4 T cell primed? Neither cluster appears to express DNTT and other clusters such as C11 and C8 have higher expression of other "T cell" features ie. KLRG1 and CNN3. C8 also expresses HSC markers, but C11 doesn't appear to? Are there defined expression thresholds for key genes that aide in the identification of these subsets?

• If these experimental arms were performed in the same experiment, comparison of the non-T hCD45+ cells (assuming that these cells are leukemia cells?) in the M-miR-142 group with and without T cells in Fig 6J would answer whether the effect is T cell dependent in the PDX model at least where no other cell type could contribute to the mechanism. Repeating a similar experiment, where T cells are depleted, in the immunocompetent model in Fig.6G ie. M-miR-142 vs M-miR-142 + anti-CD4 + anti-CD8 would provide the strongest evidence that the anti-leukemic effect is mediated by T cells in vivo with this therapeutic approach. Showing that miR142 deletion in T cells specifically increases leukemia growth does not

demonstrate that the efficacy of the therapeutic approach in Fig6/7 is mediated by 'restoring' T cell function. This is primarily due to the role of miR142 in T cell survival and cell cycle that the authors have demonstrated throughout the manuscript.

Minor Concerns:
• Sentence on lines 128-133 is too long and difficult to understand.
• Fig.S8a is CD4 or CD8 T cells?

Reviewer #3

(Remarks to the Author)
The authors addressed my concerns.

The only very minor thing is the font issue in Figure 5C. On my computer, the gamma and alpha in IFN-g and TNF-a look weird. But I cannot exclude that this is my adobe issue.

Reviewer #4

(Remarks to the Author)
Authors have revised the manuscript based on the comments by the reviewers and also performed significant amount of new experiments (both in vivo mouse data and scRNAseq analysis of human samples) and these new results strengthen the original data. Some minor changes could still be done to the manuscript:

C1) As authors reply that "it is possible that the difference of miR-142 levels in BC vs CP CML patients is due to difference in T cell subtypes" this should at least be commented in the discussion if not technically addressed

C2) T cell percentage comparison between CP and BP patients is not done in biologically meaningful way as Suppl Fig 1b shows % out of PB meaning probably percentage from the total cell number (figure legend should also be amended). This does not show that BP patients would have lower total T cell number compared to CP patients as most probably they have different WBC counts and also different differential counts. Authors should show a figure reporting T cells from lymphocyte cells and then also total T cell numbers and this could be compared for the reference values from healthy controls. Also reviewer 3 commented that the results should be shown in absolute cell numbers.

Reviewer #5

(Remarks to the Author)
I co-reviewed this manuscript with one of the reviewers who provided the listed reports. This is part of the Nature Communications initiative to facilitate training in peer review and to provide appropriate recognition for Early Career Researchers who co-review manuscripts.

Version 2:

Reviewer comments:

Reviewer #2

(Remarks to the Author)
The authors have adequately updated the statistical analyses in the figures and included additional discussion that recognizes the possibility that reduced T cell numbers may contribute to the loss of leukemic control in vivo.

My remaining concern is that the authors have not consistently updated the results text to align with the new statistical analyses. This is particularly evident in lines 277 - 288 where the old P values and statements of statistical significance between groups are included. Also lines 519-522. A thorough check that the results text p-values and conclusions of statistically significant differences matches the figures throughout would address any remaining concern.

This manuscript contains a very large body of work and all authors should be congratulated for their efforts.

Open Access This Peer Review File is licensed under a Creative Commons Attribution 4.0 International License, which permits use, sharing, adaptation, distribution and reproduction in any medium or format, as long as you give appropriate credit to the original author(s) and the source, provide a link to the Creative Commons license, and indicate if changes were made.
In cases where reviewers are anonymous, credit should be given to 'Anonymous Referee' and the source.
The images or other third party material in this Peer Review File are included in the article's Creative Commons license, unless indicated otherwise in a credit line to the material. If material is not included in the article's Creative Commons license and your intended use is not permitted by statutory regulation or exceeds the permitted use, you will need to obtain permission directly from the copyright holder.
To view a copy of this license, visit https://creativecommons.org/licenses/by/4.0/

Point-by-point reply to the Reviewers

Reviewers' comments:

Reviewer 1.

C: The manuscript is well written, comprehensive, and it includes several murine models and elegant experiments, all of which add to a careful description of this newly discovered and important pathway.

R: We appreciate the positive comments.

Major points:

C.1: Suppl. Fig 3b,c: Why are gamma delta T cells more affected than alpha beta T cells?
R.1: We apologize for the lack of clarity and want to thank the Reviewer for his/her insightful comment. Considering cell numbers, the alpha beta T cells were significantly reduced in *Mir142*$^{-/-}$*BCR-ABL* LMPP recipients compared with *Mir142*$^{+/+}$*BCR-ABL* LMPP recipients. We have now replaced the percentage of T cell subtypes with absolute cell numbers to convey this information (see Supplementary Fig. 6a, lower panel and page 8).

C.2: A quantification of all flow plots (for several mice) is needed.
R.2: Done.

C.3: Please describe the nature of the miR compound.
R.3: Done (see Methods).

C.4: Figure 6i: More human samples are needed for the PDX models. One sample is not sufficient.
R.4:, In our lab, we have tested several BC CML samples from our institutional Hematopoietic Tissue Bank for engraftment rate in immunodeficient mice. The PDX model we presented in the original manuscript was the one with the highest leukemic cell engraftment rates and the shortest survival (~8 weeks). Two additional samples showed excellent leukemic cell engraftment rates (30-60%), but the hosts survived significantly longer (>16 weeks). Thus, we used only the former PDX to test the impact of M-miR-142 treatment on survival, given the long elapsed time-to-death of the other two PDXs. In order to respond to the Reviewer's critique, we have now used one of the "high engraftment-long survival" PDXs and treated the mice with the autologous human T cells and either M-miR-142 or scramble RNA. Differences in the BM leukemic burden and T cell numbers were used as surrogate endpoints rather than survival to compare the impact of M-miR-142 treatment. This experiment confirmed that M-miR-142 treatment associated with decrease in leukemia burden and increase in T cells (see Page 19, Fig. 6j).

C.5: Please describe the GvHD in the mice treated with T+M-miR-142. Why did the T cells in these mice transplanted with autologous human T cells expand uncontrollably?
R.5: Human T cells engraft and grow well in immunodeficient NSG/NSGS mice and GVHD often develops in the host mice. Therefore, we usually perform T cell depletion before transplanting primary AML samples into NSG/NSGS mice. In the reported experiment, however, T cells were the treatment. In fact, PDX mice received autologous patient T cells alone or T cells +M-miR-142. Since M-miR-142 expanded T cell number and activity, it was not surprising that the engrafted mice, which survived longer, eventually developed and died of GVHD. In fact, these mice had

very low or no leukemia burden (<5% in PB and BM and spleen), high T cell percentage (>50%), and GVHD symptoms (i.e., diarrhea, weight loss, fur ruffling, hunching, and lethargy; see Page 20).

C.6: It would be informative to stratify human BC CML patients by expression of miR-142 and plot survival data.
R.5: Unfortunately, our sample size was too small to test miR-142 deficit as an independent prognostic factor and we were not able to find any publicly available databases with miRNA levels and survival data of BC CML patients. Of note, previous reports suggested that in CML patients, miR-142 downregulation activates antiapoptotic mechanisms (PMID: 34386429) and that lower miR-142 levels were associated with resistance to TKIs (PMID: 32999756). Furthermore, Ferreira et al., in a study of 75 CML patients and 58 healthy individuals, observed lower levels of miR-142 expression in patients with accelerated phase CML compared with those in chronic phase or healthy individuals (PMID: 24629639). Taken altogether, these results suggest that lower miR-142 levels may negatively impact on CML evolution and clinical outcome. We have added these considerations to the discussion (see Page 22).

C.7: Figure 2b and others: Why do the authors use B cell markers and negativity for CD19 rather than myeloid markers? Or why not use CD4 instead of CD25?
R.7: We used CD19 and CD11b for B and myeloid cell differentiation respectively and gated on CD19-CD11b- cells to further evaluate T cell differentiation. In the early stage of in vitro differentiation assay of LMPP (day 6), almost all cells are in a double negative (DN) stage (DN1 to DN4, based on the expression levels of CD25 and CD44). These cells do not express CD4 or CD8, thus these markers are not shown in the figures. At a later stage (Day 19), LMPP cells differentiated into double positive (DP) and eventually single positive (SP) cells, and therefore CD4 and CD8 are shown in the figures.

C.8: Figure 2d: How do you explain the increase in myeloid cells mechanistically in Mir-142 -/- mice?
C.8: Based on our data, decreased miR-142 levels seemingly redirect hematopoietic progenitors toward myeloid differentiation. Thus, we hypothesize that miR-142 deficit is part of mechanisms of “emergency myelopoiesis”, which are eventually amplified by BCR-ABL. The hijacking of these mechanisms has been reported in myeloid leukemogenesis (PMID: 28355185).

C.9: **Is the function of Treg cells affected by IL-6, or deficiency of MiR-142?**
R.9: We observed a reduction of Treg in miR-142 KO mice compared with the wt controls. However, we did not evaluate the function of Treg cells partly because selection of Treg cells required fixing the cells thereby preventing their availability for additional functional studies. Furthermore, the impact of miR-142 deficiency on Treg was already reported by Dr. Mark Boldin, collaborator and co-author on this manuscript (PMID: 35180231). Their study showed that mice with Treg cell–specific miR-142 KO (i.e., miR-142$^{fl/fl}$Foxp3Cre+ mice) developed a fatal systemic autoimmune disorder due to a breakdown in peripheral T-cell tolerance. Mir142$^{fl/fl}$Foxp3Cremice (miR-142 KO in Treg only) displayed a significant decrease in the abundance and suppressive capacity of Treg cells. Those findings implied that miR-142 is not a disposable regulator of Treg homeostasis. In our models, however, while reduction of Treg could have potentially increased Teff, it did not seem to confer enhanced antileukemic immune activity. We have added these considerations to the discussion (Page 23).

C.10: **Figure 6d: It seems as if the blasts have differentiated? How and why?**
R.10: Changes in blasts' metabolisms may provide some insights into the mechanistic bases of this observation. We refer the Reviewer to our recent work showing that M-miR-142 rescued the blastic phenotype by acting on OxPhos and mitochondrial dynamics (Nature Communications, 2023; PMID: 37658085).

C.11: **Figure 7c: A better image is necessary for the vehicle group, as the presented cells do not look like blasts, but more like mature neutrophils.**
R.11: The image of the vehicle group has been replaced.

C.12: Figure 7e: Did the combination treatment really keep all but one mouse alive? What did the bone marrow look like in the mice which survived, i.e. what cell types survived the treatment to cause death in 2 mice in the secondary transplantation experiment?
R.12: Yes, the combination treatment was very effective. When we submitted the manuscript, we had already monitored the treated mice for 100 days and at that time, only one mouse in the TKI+M-miR-142+PD-1 antibody combination group had died of leukemia. One additional mouse from this group died of leukemia on Day 106. We euthanized all remaining mice on Day 120 and found 0-10% leukemic cells in PB/BM/spleen after 4-month follow-up. Another cohort of mice (n=5 mice per group) were euthanized after completion of treatment and BM cells were pooled and transplanted into the 2nd congenic recipient mice. When we submitted the manuscript, we had already monitored the 2nd recipients for 100 days. To date, after 150 days of follow-up, only 2 recipients from donors treated with the combination had died of leukemia, suggesting LSC depletion in the treated donors. We have updated the survival data in the revised manuscript (Figs. 7e-f).

Minor points:

C. Suppl. Fig. 3C: Please fix the legend on the y-axis.
R. This figure has been removed from the revised manuscript in response to other Reviewers' comments (see Reviewer 3, Minor Concern 1).

Reviewer 2.

C: This manuscript is clear with logical, well thought out mechanistic experiments to address hypotheses. The data supporting the role of miR-142 in T cell persistence/expansion and apoptosis is strong and well supported. The data in Figure 7 are also really nice. However, the data relating to the role of miR-142 in T cell exhaustion and differentiation require additional evidence to support the current conclusions or the conclusions should be softened.

R: We appreciated the positive comments. We have softened our interpretation of the data.

Specific comments:

C.1: **Throughout the results section, the authors make comments that slightly overinterpret the data or are partially unsupported (line 329 for example). It detracts from the strength of the mechanistic experiments and the manuscript would be greatly enhanced by checking that supportive evidence is presented and subsequently editing these sentences as necessary**.

R.1: We have carefully reviewed the manuscript and modified it accordingly.

C.2: The authors focus on changes in T cell differentiation in figure 3. The overall reduction of T cell numbers makes the changes in frequency hard to interpret. Providing absolute counts for the flow cytometry data will be much clearer and more interpretable.

R.2: Done (absolute numbers were provided in Supplementary Fig. 1f in the revised manuscript).

C.3: Figure 3 human data: How were the human cells stimulated to measure cytokines? In (D) Was the phenotype of the control vs KD T cells similar prior to stimulation? Ie. did they have the same proportion of effector cells? In (E) was production of IFNg or TNF impacted?

R.3: Cytokine production was determined using flow cytometry after T cells were treated with cell stimulation cocktail (PMA plus ionomycin) and protein transport inhibitor (Brefeldin A and Monensin) for 5 hours. This has been added to the Methods (Page 27). Prior to miR-142 KD, human T cells were cultured with CD3/CD28 Dynabeads overnight to increase transduction rate. No significant difference in subpopulations was observed in the miR-142 KD T vs control T cells prior to stimulation by flow cytometry (both had ~ 70% T effector memory and ~ 20% T central memory cells; see the inserted **Figure 1** here). IFN-γ levels were also found to be significantly reduced in T cells from BC CML patients compared with those from CP CML patients. This result has been added to the revised manuscript (Page 9; Fig. 3d).

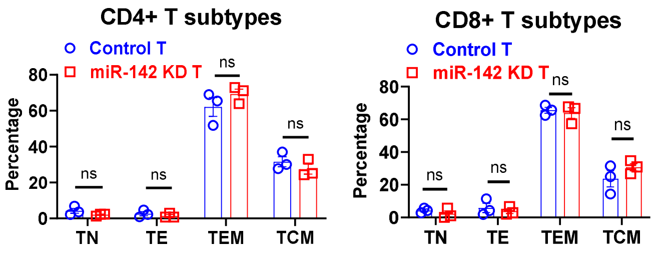

Figure 1. Cell subpopulations in miR-142 KD T vs control T cells prior to stimulation, analyzed by flow cytometry.

C.4: Figure 4 clearly demonstrates that Mir142 KO T cells do not engraft/expand in secondary recipients and it is evident that miR-142 is crucial for persistence of antileukemic T cells. However, the conclusion that these T cells have impaired function/killing activity is not supported.

R.4: If we consider only the in vivo results, we will agree with the Reviewer. However, our conclusion is based on both *in vitro* and *in vivo* results. *In vitro*, we showed an impaired function and killing activity of miR-142 KO T cells (Fig. 3b-c; Supplementary Figs. 8a-d and 9a-d). *In vivo*, we showed reduced T cells, increased leukemic burden and shorter survival of the mice receiving miR-142 KO T cells compared to controls receiving miR-142 wt T cells (Figure 4a-j). Of note, we also generated T-specific Mir142 KO BCR-ABL model ($Mir142^{flox/flox}$/Lck-cre/BCR-ABL) and found that BCR-ABL mice with T-specific Mir142 KO have more aggressive disease and shorter survival (new data, Fig. 4k in the revised manuscript). Thus, we postulated that miR-142 KO may confer

a reduced antileukemic activity of T cells possibly due to reduced number and decreased immune activity. We have added the new results and clarified/softened our conclusions in the revised manuscript (see page 11-12; Figs. 3 and 4; Supplementary Figs. 8 and 9).

<u>C.5</u>: Throughout the manuscript T cell numbers need to be enumerated and presented as total counts not frequencies. Frequencies in blood will always be lower in untreated mice due to high numbers of circulating blasts. If total numbers are not possible, % within CD45.1+ cells to exclude blasts could be presented instead.

<u>R.5</u>: Frequencies of T cells have been replaced with absolute counts in the revised manuscript (Figs. 6c, 6g, 6k, 7d; Supplementary Figs. 1f and 6a, lower panel), with no changes in the original results and conclusions.

<u>C.6</u>: Figure 6: T cell depletion experiments need to be performed in the immunocompetent model to prove that the effect of the M-miR-142 is T cell mediated. The therapy is interesting regardless of whether it is acting directly on T cells or by directly inhibiting leukemia cell growth. However, if the authors wish to conclude that the mechanism is T cell-dependent, this needs to be definitively demonstrated. The PDX model in (J) suggests that there are T cell independent mechanisms and this needs to be discussed.

<u>R.6</u>: We will be happy to address this critique, but we may require some clarifications since Figure 6f-h already depicted results from immunocompetent mice. Figure 6i-k (Figure 6i-l in the revised manuscript) used immunodeficient mice since human BC CML cells do not otherwise engraft in immunocompetent mice. We also generated T-specific Mir142 KO BCR-ABL model (Mir142$^{flox/flox}$/Lck-cre/BCR-ABL) and found that BCR-ABL mice with T-specific Mir142 KO have more aggressive disease and shorter survival than BCR-ABL mice with T-Mir142 wt (new data, Fig. 4k in the revised manuscript). Taken altogether, our *in vivo*, *ex vivo* and *in vitro* results suggest an impaired anti-leukemic activity of miR-142 KO T cells. We agree and are not surprised that the results from the PDX model suggest also other T cell independent mechanisms, as M-miR-142 also impacts on LSCs (Nature Communications, 2023; PMID: 37658085). We have added these considerations to the discussion in the revised manuscript (see Page 22; see also C.4. and R.4).

<u>C.7</u>: There is no clear evidence to support the conclusions related to T cell exhaustion and hypofunctionality. If the authors wish to focus on this, supporting evidence should be moved into main figures and sustainably strengthen with in vivo killing assays using purified TEFF populations with and without an miR-142 deficit.

<u>R.7</u>: Regarding to T cell hypofunctionality, we showed *in vitro*, an impaired function/killing activity of both murine and human miR-142 KO T cells (Figure 3b-c; Supplementary Figs. 8a-d and 9a-d) and *in vivo,* reduced T cells, increased leukemic burden and shorter survival of the leukemic mice that received miR-142 KO T cells compared with those that received miR-142 wt T cells (Figure 4a-j). T-specific Mir142 KO BCR-ABL (Mir142$^{flox/flox}$/Lck-cre/BCR-ABL) mice also had a more aggressive leukemic phenotype and shorter survival than T-Mir142 wt BCR-ABL mice (**New data**; Fig. 4k in the revised manuscript). Of note, since miR-142 KO T cells have higher frequencies of effector T than wt T cells, when we co-transplanted LSCs and the same numbers of miR-142 KO or wt T cells, mice transplanted with miR-142 KO T cells actually received more

effector T with miR-142 deficit. Despite this, mice receiving miR-142 KO T cells had higher disease burden and shorter survival than those receiving wt T cells. When we consider all these observations collectively, our conclusion is that the anti-leukemic activity of miR-142 KO T effectors was likely impaired. Nevertheless, we have softened our conclusions in the revised manuscript (Page 23).

Minor concerns:

C.1: The colors for the miR-142 non-tumor controls in Figure 1 cannot both be black.

R.1: Corrected.

C.2: Line 188, how were cells stimulated to measure cytokines? This should ideally be included in the text.

R.2: For both mouse and human T cells, cytokine production was determined using flow cytometry after T cells were treated with cell stimulation cocktail (PMA plus ionomycin) and protein transport inhibitor (Brefeldin A and Monensin) for 5 hours. This information has been added to Methods (Page 27).

C.3: Line 329. Is there data to support the conclusion that the IL-6 is being produced by blasts? If not, the "blast-produced" part of this sentence could be removed.

C.3: We have removed “blast-produced” in the revised manuscript.

C.4: Line 334: PD-1 is an activation marker and not an exhaustion marker.

R.4: Corrected.

C.5: Lines 352 - 356: it should be made clearer that there is an association between reduced miR-142 levels and increased expression of inhibitory T cells as the evidence does not support causation here.

R.5: Done.

C.6: The terminology for TKI is different between the text (TKI) and figure (NIL) in Figure 7.

R.6: Corrected.

C.7: "Engraftment" terminology is confusing.

R.7: We now use donor-cell derived leukemic burden instead of engraftment in the revised manuscript.

Reviewer 3.

Major Concerns:

C.1: The authors showed in sup fig 1a that the reduction of miR-142 in patients is only about 40%. This raises major questions regarding the meaning and significance of using miR-142 homozygous KO to model the human condition. The authors could examine the effect of miR-142 heterozygous loss, especially in T cells, in controlling CML progression.
R.1: We appreciated the insightful Reviewer's comment and have then performed the suggested, additional experiments to investigate a dose-dependent effect of miR-142 deficit using Mir142+/- T cells (i.e., heterozygous for miR-142 KO). We cocultured wt or Mir142+/- (heterozygous loss) T cells with BC CML LSCs *in vitro* for 3 days. Like Mir142-/- T cells (homozygous for miR-142 KO), Mir142+/- T cells (heterozygous for miR-142 KO) displayed reduced antileukemic activity (i.e., reduced cytokine production, increased spontaneous apoptosis, and reduced anti-LSC activity) compared to wt T cells (see Fig. 3b, right panel; Supplementary Figs. 8d). To characterize *in vivo* *Mir142*$^{+/–}$ T cells, we co-transplanted BC CML LSCs and *Mir142*$^{–/–}$, *Mir142*$^{+/–}$, or *Mir142*$^{+/+}$ T cells into NSG mice which lack T cells (Fig. 4c-d). We also transplanted BC CML LSCs into *Mir142*$^{–/–}$, *Mir142*$^{+/–}$, or *Mir142*$^{+/+}$ recipient mice (Fig. 4e-f). In both experiments, we observed a significantly shorter survival in recipients of *Mir142*$^{+/–}$ T cells compared with recipients of *Mir142*$^{+/+}$ T cells. These results indicate that even a partial miR-142 deficiency may affect T cells' numbers and activity.

C.2: The genetic backgrounds of the mice are critical in interpreting the T-cell-based anti-leukemic effects. Are these due to immunity against leukemic cells or against the genetic background the leukemic cells are on, especially if there are any mixed background effects? For BCR-ABL model and miR-142 KO or fl/fl, the method says they are on the B6 background. Which B6 strain (J, N, or others?) and the history of creation and backcrossing?

R.2: We have published these models, and they are all in C57Bl/6j background (Nature Medicine; 2018, PMID: 29505034; Nature Communications, 2023, PMID: 37658085). We have clarified the background of these models in the Method section of the revised manuscript (Page 26-27).

C.3: There is no direct evidence for a T cell-response toward leukemia. Many of the data presented can be interpreted equally as an indirect effect (which can be many possibilities). It will be very helpful to examine whether T cell activation status is affected by the presence of BC-CML cells directly, such as via in vitro co-culture experiments.

R.3: We have already provided these data in the original manuscript. T cell activation toward leukemia was shown with in vitro co-culture of T cells with BC CML cells (Supplementary Figure 12d-e) and in vivo transplant of BC CML cells into congenic normal wt recipients (Figure 5a-e). In vitro experiment showed that T cells co-cultured with BC CML blasts have lower miR-142 levels and cytokine production than T cells co-cultured with CP CML mononuclear cells (MNCs) or normal MNCs (Supplementary Figure 12d-e). In vivo experiment showed that host T cells from the recipients of BC CML blasts have lower miR-142 levels and cytokine production and higher spontaneous apoptosis rate and cell cycling arrest at 2 weeks and lower numbers in PB, BM and spleen at 4 weeks post transplantation than host T cells from the recipients of CP CML MNCs or normal MNCs (Figure 5a-e). These results provide direct evidence for a T cell-response toward leukemia.

<u>C.4</u>: Does miR-142 affect the output of NK, B, DC cells from LMPP?

<u>R.4</u>: In our co-culture system, we used a OP9-DL1, which is a BM-derived stromal cell line that ectopically expresses the Notch ligand, Delta-like 1 and promotes in vitro T-lymphocyte differentiation. Thus, no NK, B or DC cell outputs were identified. The role of miR-142 in other immune cells besides T cells was also reported. MiR-142 reportedly regulates differentiation and activation of B, dendritic cells (DC), T, NK, and myeloid cells and loss of the mir142 gene in the mouse results in decreased hematopoietic output, and reduction of T, B and NK cells (PMID: 25931583; PMID: 30958798; PMID: 26098216; PMID: 31402259). While our study showed that miR-142 deficit affects the output of T cells from LMPP and the activity of mature T cells, changes in other immune cell subpopulations may also occur and contribute to the immune escape and disease growth. We have added this consideration into the discussion (Page 25). However, the studies of immune cells other than T cells are beyond the scope of the present manuscript and will be pursued in the future.

<u>C.5</u>: For miR-142 inhibitor, can the authors clarify why both 5p and 3p inhibitors work? Does the 5p inhibitor affect the 3p miR-142? And does 3p inhibitor affect 5p level?

<u>R.5</u>: Our data supports that both miR-142-3p and -5p play a role in mediating PD-1 expression on T cells and PD-L1 expression on LSK cells, and in turn in promoting LSK immune escape of T cell immune surveillance. The 3p inhibitor inhibits only miR-142-3p and the 5p inhibitor inhibits only miR-142-5p (see the inserted **Figure 2** here).

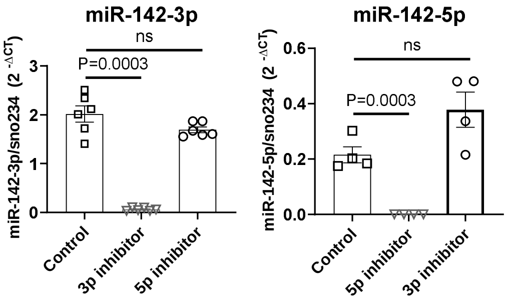

Figure 2. miR-142-3p and -5p levels in T cells treated with miR-142-3p inhibitor or -5p inhibitor (1μM) for 24 hours, measured by Q-RT-PCR.

<u>C.6</u>: There are many descriptions of the changes in miR-142 null T cells or T cell differentiation, but the mechanisms are not carefully addressed. Is TGF-b pathway the key reason? The authors need to at least discuss this aspect. Introduction of known functional targets in immune cells could aid readers in gaining a more comprehensive understanding.

<u>R.6</u>: The mechanisms through which miR-142 deficit impacts on T cell differentiation and activity is likely multifactorial. It is well known that microRNAs regulate concurrently the expression of several target genes involved in distinct cellular processes, thereby controlling these processes at multiple levels. Herein, we point out that one of the potential mechanisms through which miR-142 deficit impacts on T cells is blockage of the "metabolic shift" necessary for T cell activation, proliferation and survival. TGF-β is also a known regulator of T cell differentiation and activity (TGF-β: A Master of All T Cell Trades. Li, Ming O. et al. Cell, 134 (3), 2008; TGF-β Regulation of T Cells, 2023, https://doi.org/10.1146/annurev-immunol-101921-045939). Our data showed that miR-142 deficit enhanced TGF-β signaling in T cells. While we focus on how the enhanced TGF-β signaling leads to PD-1 upregulation (PMID: 28302134; PMID: 24558198; PMID: 28823564), it is very likely that it may also affect other aspects of T cell homeostasis and activity as previously reported (PMID: 28302134; PMID: 24558198; PMID: 28823564; PMID: 31402259). As suggested by the Reviewer, we discussed these aspects in the revised manuscript (see page 23-24).

Minor Concerns:

C.1: The manuscript has a large amount of data, some of which are not obviously meaningful. The authors can consider reducing those areas. For example, much of the scRNAseq and metabolomic studies is descriptive, neither proves or disproves a key point, and is challenging for readers to read through. For scRNAseq, the assignment of clusters is questionable.

R.1: We have removed some of the results as suggested by the Reviewer to attenuate some unnecessary redundancy.

C.2: For scRNAseq, there are supposed to be three mice for each condition. The authors should show (if they want to continue to include those sections) the statistics with mean and std, e.g. for cluster size.

R.2: We pooled cells from three mice and did scRNA-seq, so there is one pool per mouse strain.

C.3: Fig 5c: the font for IFN-g and TNF-a is problematic?

R.3: Corrected.

Reviewer 4.

C: Overall, studies are carefully done utilizing a lot of various mouse models, but it is still a bit difficult to understand the role of miRNA142 as that does not seem to have effect alone on immune cell development and changes are only observed in the blast phase model.

R: miR-142 deficit has impact both on LSCs and T cells (and its impact probably extends also on NK and DC). We previously reported on how miR-142 as a single microRNA can affect LSC homeostasis thereby contributing to disease development and growth (Nature Communications, 2023, PMID: 37658085). Herein, we focus on the effect of miR-142 deficit on T cells and leukemia immune escape and in turn leukemia growth. To this end, we used in vitro co-culture system (LSC+WT T vs LSC+KO T), in vivo transplant (LSC to wt or hom-KO or het-KO recipients; LSC to wt or global KO or T-specific KO recipients), and Mir142flox/flox/Lck-cre/BCR-ABL model (T-specific Mir142 KO, new data) to sort out the effect of miR-142 deficit on T cells (Figs. 3b and 4a-k). Once these two papers (the current one and the PMID: 37658085) are considered together, we hope that a concept emerges describing how leukemogenesis may be driven by a mechanism (i.e., miR-142 deficit) that while disrupts similar process/pathways in distinct cellular populations of the BM niche (e.g., increase in mitochondrial oxidative metabolism), it causes opposite effects in these distinct cell populations (e.g., expansion of LSCs and contraction of T cells). We added some of these considerations in the discussion of the revised manuscript (see Page 24).

More specific comments

<u>C.1</u>: Key starting point in results is that blast phase CML patients have lower levels of miRNA142 than controls or CP patients and this is based on the supplementary figure 1A. However, this figure does not prove that this is the case, as it could also be that levels of miRNA142 can be similar in specific T cell types and just some T cell subtype is decreased in BP CML patients. Authors should use other metrics to show that expression levels of miRNA are really decreased in BP-CML patients (single cell analysis?) or that there is no difference in their T cell subtypes compared to controls or CP patients

<u>R.1</u>: Herein, we reported reduced levels in the total T cells from BC CML patients vs those from CP CML patients using Q-RT-PCR. Using flow cytometry, we also showed that like in the mouse, frequencies of Tnaïve in both CD4+ and CD8+ T cells were reduced, and frequency of effector T cells increased in BC vs CP CML patients. Thus, it is possible that the difference of miR-142 levels in BC vs CP CML patients is due to difference in T cell subtypes. Unfortunately, scRNA-seq would not allow us to detect microRNA levels in single cell level; sc-miRNA-seq allows to detect microRNA levels in single cells but would not be useful to identify cell subtypes. Also, we could not obtain sufficient cells to assess miR-142 levels in each T cell subpopulation.

<u>C.2</u>: In the next sentence authors claim that "Accordingly, using genetically engineered mouse models (GEMMs), we also observed that the BC Mir142−/−BCR-ABL mouse presented with significant T lymphopenia…" But this is quite different what is observed in patients as they do not have T lymphopenia? Or do authors have some evidence that there is T cell lymphopenia in patients? Furthermore, the T cell data in mouse models in suppl fig 1 is shown as percentage of all cells and is there real difference among absolute numbers? Ie is it just that the total WBC count is increased and there are more other cell types?

<u>R.2</u>: We appreciate the Reviewer's comment. We found that the T cell percentages in BC CML patients was significantly reduced compared to that of CP CML patients (see Supplementary Fig. 1b). In addition to percentages of T cell subtypes in mouse models, we also included absolute numbers of T cell subtypes in the revised manuscript (Supplementary Fig. 1f). Also, we have evidence that even a partial miR-142 deficit may decrease T cells' numbers and activity (see Reviewer 3 C1-R1; Fig. 3b and 4c-f).

<u>C.3</u>: In Figure 1C single cell data is shown from various mouse models which suggest that miRNA deficit alone does not change stem cell compartment but actually the formation of blast crisis changes it. Some comparison to patient single cell data would be needed here (data available from previous published studies)

<u>R.3</u>: If the Reviewer refers to changes in human LMPP, unfortunately, we were not able to find publicly available single cell data of LMPP (Lin−CD34+CD38−CD45RA+CD90−) from BC CML vs CP CML patients. Thus, we performed scRNA-seq of LMPP ($Lin^{-}CD34^{+}CD38^{-}CD45RA^{+}CD90^{-}$) from BC CML vs CP CML patients. Like in the mouse, we found that LMPP cells from BC CML patients also have reduced T-primed subpopulation compared to LMPPs from CP CML patients (Page 6; Supplementary Figs. 4a-b and 5a-b). Thus, it is possible that the presence of BCR-ABL or the formation of blast crisis is required to change LMPP compartment, reducing their potential for T cell differentiation.

C.4: Did authors also study NK cell development? If LMPP was myeloid biased, which cells were produced then?

R.4: Berrien-Elliott et al. (PMID: 31402259) studied the role of miR-142 in NK cells and found miR-142 is critical for the homeostasis and function of type 1 innate lymphoid cells. Global miR-142-deficient mice exhibited an expansion of ILC1-like cells concurrent with increased TGF-β signaling. Further, miR-142-deficient mice had reduced NK-cell-dependent function and increased susceptibility to murine cytomegalovirus (MCMV) infection. Thus, miR-142 critically integrates environmental cues for proper type 1 ILC homeostasis and defense against viral infection (PMID: 31402259). We also compared subpopulations and activity of NK cells in BC CML vs CP CML mice and observed similar changes (unpublished data). Further, we transplanted BC CML LSCs into the congenic recipients with NK-specific KO of miR-142 ($Mir142^{flox/flox}$Ncr-cre+) or wt ($Mir142^{flox/flox}$Ncr-cre-) recipients and observed no significant changes in leukemic burden and survival (unpublished data), suggesting that NK-specific KO of miR-142 does not affect their in vivo anti-LSC activity. While these experiments are an extension of the present work, they are beyond the scope of the current manuscript and will not be presented, given also the amount of data presented in the revised manuscript.

In the co-culture of LMPP with OP9-DL1, LMPP with miR-142 KO was myeloid primed. Since this culture system favors T cell differentiation, mainly T lymphocytes were produced, with delayed and much fewer T mature cells produced by miR-142 KO LMPP. We expect that LMPP from miR-142KO/BCR-ABl mice will preferentially differentiate into GMP and myeloid cells, since one of our hypotheses is that miR-142 deficit may be part of the mechanisms of emergency myelopoiesis that are hijacked during leukemogenesis (PMID: 28355185).

C.5: In vivo anti-leukemic activity. If understood correctly, miRNA142 KO LSK cells were co-transplanted with Mir142+/+ T or Mir142-/- T cells (but not BCR-ABL pos)? How would authors interpret these results as their earlier results mostly showed that the T cell impairment was mostly seen in miRNA142-/- BCR-ABL mice LMPPs and not with WT miRNA142-/-?

R.5: Although using scRNA-seq, we showed that $Mir142^{-/-}$ BCR-ABL LMPPs, but not $Mir142^{-/-}$ LMPPs or BCR-ABL LMPPs, had reduced T-primed subpopulation; when we performed functional assays, we found that miR-142 deficit in LMPPs actually also impaired T cell differentiation regardless of the BCR-ABL expression. In fact, when we transplanted LMPP into congenic normal wt recipient mice, both Mir142-/- BCR-ABL LMPPs and Mir142-/- LMPPs showed similarly impaired T cell differentiation in thymus at 2 weeks and in PB, BM and spleen at 4 weeks compared with Mir142+/+ BCR-ABL LMPPs and Mir142+/+ LMPPs respectively (Figure 2e-h; Supplementary Figure 6a-b).

We also observed impaired activity of T cells from Mir142-/- BCR-ABL mice vs those from Mir142+/+ BCR-ABL mice and of T cells from Mir142-/- mice vs those from Mir142+/+ mice. We co-transplanted Mir142 KO LSK cells with Mir142+/+ T or Mir142-/- T cells, but not BCR-ABL positive Mir142+/+ T or Mir142-/- T cells, to test the effect of miR-142 deficit on T cells in the absence of BCR-ABL expression in T cells. Of note, T cells from the BCR-ABL mice have very low BCR-ABL expression since its expression in this GEMM, was driven by the SCL gene promoter.

Our results suggest that Mir142-/- LMPPs, regardless of the presence or absence of BCR-ABL, presented with impaired T cell differentiation capacity and the miR-142 KO T cells, regardless of deriving from Mir142-/- BCR-ABL LMPPs or Mir142-/- LMPPs, had reduced activity. See also our reply to **C.4.,** stating that while miR-142 deficit impairs T homeostasis and function, the presence of BCR-ABL may preferentially redirect miR-142-/- LMPPs differentiating toward the myeloid lineage through mechanisms like those involved in emergency myelopoiesis that are potentially hijacked during myeloid leukemogenesis (PMID: 28355185).

C.6: Deficit of miRNA142 and PD1 expression. As authors show earlier that miRNA deficit is causing reduction of naïve cells could that be reason for increased PD1 expression as naïve cell are known to express lower levels of PD1? Ie no direct effect on PD1, but change in the subpopulations of T cells? Was that observed spontaneous apoptosis more pronounced in naïve cells? Similarly in patient samples, PD1 and other checkpoint expression should not only be shown in T cells, but in subpopulations as it has already been shown before even in CML that different T cell subpopulations have different expression levels of checkpoint receptors

R.6: First, to address the question if increased PD1 expression is due to changes in the T cell subpopulations, we showed that while both in CP CML and BC CML mice, T effector/effector memory cells have higher PD-1 levels than naïve T cells. CD4+ T cell subtypes (i.e., naïve and effector/effector memory) and CD8+ subtypes (i.e., naïve, effector/effector memory and central memory) from Mir142-/- BCR-ABL mice had higher PD-1 levels than their counterparts from Mir142+/+ BCR-ABL mice (Supplementary Fig. 14d). Likewise, while in BC CML patients, T effector and effector memory cells also have higher PD-1 levels than naïve T cells, both CD4+ and CD8+ T effector and T effector memory cells in these patients had higher levels of PD-1 than their counterparts from CP CML patients (Supplementary Fig. 15j). Therefore, we believe that increased PD-1 expression was not solely caused by the change in the subpopulations of T cells, i.e., reduction of naïve cells and increase of effectors; and that miR-142 likely has a direct effect on PD-1.

Second, CD4+ and CD8+ T cell subtypes (i.e., naïve, effector/effector memory and central memory) from Mir142-/- BCR-ABL mice have higher apoptotic rates than those from Mir142+/+ BCR-ABL mice (Supplementary Fig. 8c). Both CD4+ and CD8+ T effector/effector memory cells from BC CML patients had higher apoptotic rates than those from CP CML patients (Supplementary Fig. 9e). These results suggest that the increased spontaneous apoptosis was not more pronounced in naïve cells, but rather it was observed across all murine T cell subpopulations and in human effector/effector memory T subpopulations in BC CML. These results have been added to the revised manuscript (Page 9 and 16; Supplementary Figs. 8c, 9e, 14d and 15j).

C.7: The discussion should take into account points above and also critical discussion how well this presents situation in blast phase patients. In addition, it is well known that blast phase can occur during multiple mechanisms and for example acquisition of new driver genes which could also have impact on immune cell function and immune evasion

R.7: We have modified the discussion to reflect the above points (Page 22-24).

Reviewer 5.

C: I co-reviewed this manuscript with one of the reviewers who provided the listed reports. This is part of the Nature Communications initiative to facilitate training in peer review and to provide appropriate recognition for Early Career Researchers who co-review manuscripts.

R: We are grateful to the Reviewer for supporting this Nature Communications initiative.

Point-by-point reply to the Reviewers

Reviewers' comments:

Reviewer #1:

C.1. The only questions that remains to this reviewer is whether there is reduced 'homing' of KO T cells (Figure 4b), which could explain this finding.
R.1. We thank the Reviewer for the suggestion. However, we believe that homing assays for already differentiated T cells may not be as informative as those for LMPPs. While LMPPs are known to home into thymus, mature T cells can home into different tissues and organs, which makes it particularly challenging to estimate the true "homing rates" correctly.

Reviewer #2:

C.1. It appears that all the stats are t-tests according to the figure legends? Were corrections for multiple comparisons performed? It is not appropriate to present multiple groups in the same plot without correcting for multiple comparisons. The data most likely to be affected by this are the quantification of WBC, leukemic cells and T cells in Fig.7B, D, F, I and J.
R.1. The statistical analyses were redone by Dr. Jianying Zhang, our Leukemia Center statistician and now a co-author in the paper. In the revised manuscript and figures, all comparisons among multiple independent groups were assessed by one-way ANOVA or log-rank test (for survival data), with P values corrected for multiple comparisons using Holm-Šídák method.

C.2. Fig1D, what is the expression level of key genes in each subset? A dot plot depicting gene expression and percent expressed was generated for the human dataset and it would be helpful if this was shown for the mouse sequencing as well.
R.2. We provide these data in Fig 1d of the revised manuscript.

C.3. In Fig.3A a stacked bar graph (or something similar) would be helpful to visualize changes in T cell subset distribution. The UMAPS do not obviously depict the observed changes in frequency.
R.3. We provide these data in Supplementary Fig. 7a of the revised manuscript.

C.4. In Fig.3B the T cell viability is reduced. As effector to target ratios decrease, the killing of tumor cells decreases. Killing assays usually show a curve with several E:T ratios and demonstrate reduced killing at lower ratios regardless of whether the effector T cells can capably kill tumor cells at higher ratios. This is not a readout of loss of T cell function and is rather related to the physical ratio of the T cells to the target. The changes in viability/apoptosis/cell growth in both the mouse and human KO T cells are much larger than the small decreases in cytokine production (many of which are not statistically significant) throughout the manuscript. These data do not demonstrate that Mir142 KO T cells have reduced killing capacity (are there changes in cytolytic proteins such as granzyme, CD107a, perforin?). These data rather support the conclusion that Mir142 KO cells have decreased survival/cell cycle arrest and thus have reduced anti-leukemic

activity due to reduced numbers of T cells. This is an important distinction when describing the mechanism. Persistence of T cells is crucial for responses to immunotherapy and this mechanism likely contributes to the improved survival seen in Figs. 6-7.

R.4. We appreciate the reviewer's insightful comment. We agree that the data support the view that Mir142 KO T cells have reduced anti-leukemic activity mainly due to the increased apoptosis/cell cycle arrest and in turn reduced numbers. However, it is interesting that compared to Mir142wt T cells, Mir142 KO T cells have also reduced production of cytotoxicity-associated proteins (i.e., granzyme B and CD107a; Supplementary Figure 8a in the revised manuscript), suggesting that they may also have reduced killing activity. Nevertheless, we have modified our discussion in the revised manuscript to reflect the above statement and the reviewer's view.

C.5. How are the T cell subsets quantified in Fig.S7B? The distribution of phenotypes within CD4 and CD8 T cells (the flow plots) or total cell numbers of each population as is shown in Fig.S1F would be the most informative way to quantify this. The increase in memory CD8 T cells is the most striking finding in S1F and S7B and this is not mentioned in the manuscript?

R.5. T cell subset quantifications in Fig, S7b and Fig. S1e-f are done using the same flow cytometry data from BC CML vs CP CML mice.

Fig. S1e-f showed the unbiased distribution of immunophenotypically-defined T cell subsets using total mononuclear cells (MNC) from PB, BM and spleen as denominators (Fig. S1e) and the absolute cell number of each T cell subset (Fig.S1f) in BC CML vs CP CML mice.

Fig. S7b (now Fig. S7d in the revised manuscript) showed the distribution of immunophenotypically-defined T cell subsets using total T cells from PB, BM and spleen of BC CML vs CP CML mice as denominators. This figure shows the relative changes of each T cell subset in Mir142KO T cell population from BC CML mice compared with Mir142wt T cell population from CP CML mice. This figure also supported, at the protein level, the results of the scRNA-seq clustering analysis conducted on the same samples (Fig. S7a). We agree with the Reviewer that the enrichment in CD8 memory T cells in the Mir142 KO CML mouse is striking and we highlighted this finding in the revised manuscript (Page 8 and 23).

C.6. Human data in Supp Fig. 5:
o There is no significant reduction in T-primed subsets, both statistically and in magnitude.
o What is the rationale for naming C3 and C4 T cell primed? Neither cluster appears to express DNTT and other clusters such as C11 and C8 have higher expression of other "T cell" features ie. KLRG1 and CNN3. C8 also expresses HSC markers, but C11 doesn't appear to? Are there defined expression thresholds for key genes that aide in the identification of these subsets?

R.6. While it is relatively simpler to identify LMPP and their gene biases in mouse, the same analysis is more difficult in human because of the relatively lower number of cells obtained from the banked patient samples and the much reduced expression of specific lineage marker genes. Nevertheless, we attempt to parallel mouse and human findings. We named C3 and C4 as T cell primed subsets because these two clusters expressed relatively higher levels of T cell gene markers KLRG1 and CNN3, even though these cells expressed lower DNTT levels. We believe that C11 (expressing T cell gene markers KLRG1 and CNN3) is also close to other T-primed subsets, while C8 (expressing high levels of HSC markers HOPX, CRHBP, HLF and PCDH9) may represent a more undifferentiated subset. The analysis was done on the limited number of samples (n=3 samples per disease) available for scRNA-seq analysis; when we combined all T-primed clusters (C3, C4 and C11), we observed a significant reduction of this combination in BC

LMPP compared with CP LMPP cells (Fig. S5b, right panel), very close to what we observed in mice. There are no defined expression thresholds for key genes that aid in the identification of these subsets and several key lineage gene markers are not expressed in LMPPs, thus we have softened the interpretation of these results (see page 6).

C.7. If these experimental arms were performed in the same experiment, comparison of the non-T hCD45+ cells (assuming that these cells are leukemia cells?) in the M-miR-142 group with and without T cells in Fig 6J would answer whether the effect is T cell dependent in the PDX model at least where no other cell type could contribute to the mechanism. Repeating a similar experiment, where T cells are depleted, in the immunocompetent model in Fig.6G ie. M-miR-142 vs M-miR-142 + anti-CD4 + anti-CD8 would provide the strongest evidence that the anti-leukemic effect is mediated by T cells in vivo with this therapeutic approach. Showing that miR142 deletion in T cells specifically increases leukemia growth does not demonstrate that the efficacy of the therapeutic approach in Fig6/7 is mediated by 'restoring' T cell function. This is primarily due to the role of miR142 in T cell survival and cell cycle that the authors have demonstrated throughout the manuscript.

R.7. In Fig. 6J, the experimental arms were performed in the same experiment and comparison of the hCD3-hCD45+ cells (i.e., leukemia cells) in the M-miR-142 group with and without T cells showed a significant difference (P=0.0008), suggesting the effect is T cell-dependent. This information has been added in Fig. 6J of the revised manuscript. In Fig. 6k and 6i, the survival of the M-miR-142 group with and without T cells showed a significant difference (P=0.008 in Fig. 6k and P<0.001 in Fig. 6i), suggesting the differences in survival are T cell-dependent. This information has been added in Fig. 6k and 6i of the revised manuscript.

While we agree with the Reviewer that repeating a similar experiment, with T cells depletion in the immunocompetent models as in Fig.6G, ie. M-miR-142 vs M-miR-142 plus anti-CD4 and/or anti-CD8 would provide even stronger evidence that the anti-leukemic effect is T cell-mediated, these experiments are laborious and time-consuming (i.e., 3 months for the treatment and survival of the primary mice and 6 months for both primary and 2nd transplant experiments). This time far exceeds the 4-week period allowed by the Editor to amend the current version of the manuscript. Furthermore, we feel that our current *in vivo* experiments (Figs 4a-k) already provided sufficient evidence that Mir142 KO T cells have defected antileukemic activity, which, as the Reviewer suggested, is mainly driven by reduced T cell number. Accordingly, we have modified the discussion and conclusion in the revised manuscript to reflect this Reviewer's point of view.

C.8. Sentence on lines 128-133 is too long and difficult to understand.

R.8. Corrected.

C.9. Fig.S8a is CD4 or CD8 T cells?

R.9. Both CD4 and CD8 T cells were included in the revised manuscript.

Reviewer #3.

C.1. The only very minor thing is the font issue in Figure 5C. On my computer, the

gamma and alpha in IFN-g and TNF-a look weird. But I cannot exclude that this is my adobe issue.

R.1. We adjusted the gamma and alpha font in IFN-γ and TNF-α.

Reviewer #4.

C.1. As authors reply that "it is possible that the difference of miR-142 levels in BC vs CP CML patients is due to difference in T cell subtypes" this should at least be commented in the discussion if not technically addressed

R.1. Done (see page 23).

C.2. T cell percentage comparison between CP and BP patients is not done in biologically meaningful way as Suppl Fig 1b shows % out of PB meaning probably percentage from the total cell number (figure legend should also be amended). ……Authors should show a figure reporting T cells from lymphocyte cells and then also total T cell numbers and this could be compared for the reference values from healthy controls.

R.2. The percentages of CD3+ T cells presented in Suppl Fig 1b were directly calculated by the flow cytometer from the total PB mononuclear cells extracted from the patient samples that we received directly from our institution biospecimen bank. Thus, it reflects the differences in T cell content in BC vs CP samples. Unfortunately, diagnostic complete blood counts (i.e., CBC) from medical records are not available for all the included patients, and even for those available, the lymphocyte counts do not differentiate between B vs T cells. Thus, we cannot provide the requested information.

Point-by-point reply to the Reviewers

Reviewers' comments:

Reviewer #2 (Remarks to the Author):

Comments: The authors have adequately updated the statistical analyses in the figures and included additional discussion that recognizes the possibility that reduced T cell numbers may contribute to the loss of leukemic control in vivo.

My remaining concern is that the authors have not consistently updated the results text to align with the new statistical analyses. This is particularly evident in lines 277 - 288 where the old P values and statements of statistical significance between groups are included. Also lines 519-522. A thorough check that the results text p-values and conclusions of statistically significant differences matches the figures throughout would address any remaining concern.

Reply: We thank the Reviewer for your comment. We have updated the results text to align it with the new statistical analyses, including lines 277 - 288 and lines 519-522. We have thoroughly checked and confirmed that the p-values and conclusions of statistically significant differences in the results text matches the figures throughout**.**
